# Molecular Evolution of Glycoside Hydrolase Genes in the Western Corn Rootworm (*Diabrotica virgifera virgifera*)

**DOI:** 10.1371/journal.pone.0094052

**Published:** 2014-04-09

**Authors:** Seong-il Eyun, Haichuan Wang, Yannick Pauchet, Richard H. ffrench-Constant, Andrew K. Benson, Arnubio Valencia-Jiménez, Etsuko N. Moriyama, Blair D. Siegfried

**Affiliations:** 1 School of Biological Sciences, University of Nebraska-Lincoln, Lincoln, Nebraska, United States of America; 2 Department of Entomology, University of Nebraska-Lincoln, Lincoln, Nebraska, United States of America; 3 Center for Plant Science Innovation, University of Nebraska-Lincoln, Lincoln, Nebraska, United States of America; 4 Food Science and Technology, University of Nebraska-Lincoln, Lincoln, Nebraska, United States of America; 5 Department of Entomology, Max Planck Institute for Chemical Ecology, Jena, Germany; 6 Biosciences, University of Exeter, Penryn, United Kingdom; 7 Departamento de Producción Agropecuaria, Facultad de Ciencias Agropecuarias, Universidad de Caldas, Manizales, Colombia; The Ohio State University/OARDC, United States of America

## Abstract

Cellulose is an important nutritional resource for a number of insect herbivores. Digestion of cellulose and other polysaccharides in plant-based diets requires several types of enzymes including a number of glycoside hydrolase (GH) families. In a previous study, we showed that a single GH45 gene is present in the midgut tissue of the western corn rootworm, *Diabrotica virgifera virgifera* (Coleoptera: Chrysomelidae). However, the presence of multiple enzymes was also suggested by the lack of a significant biological response when the expression of the gene was silenced by RNA interference. In order to clarify the repertoire of cellulose-degrading enzymes and related GH family proteins in *D. v. virgifera*, we performed next-generation sequencing and assembled transcriptomes from the tissue of three different developmental stages (eggs, neonates, and third instar larvae). Results of this study revealed the presence of seventy-eight genes that potentially encode GH enzymes belonging to eight families (GH45, GH48, GH28, GH16, GH31, GH27, GH5, and GH1). The numbers of GH45 and GH28 genes identified in *D. v. virgifera* are among the largest in insects where these genes have been identified. Three GH family genes (GH45, GH48, and GH28) are found almost exclusively in two coleopteran superfamilies (Chrysomeloidea and Curculionoidea) among insects, indicating the possibility of their acquisitions by horizontal gene transfer rather than simple vertical transmission from ancestral lineages of insects. Acquisition of GH genes by horizontal gene transfers and subsequent lineage-specific GH gene expansion appear to have played important roles for phytophagous beetles in specializing on particular groups of host plants and in the case of *D. v. virgifera*, its close association with maize.

## Introduction

Cellulose, which is mostly synthesized by terrestrial plants and marine algae, is the most abundant organic compound on Earth. It is a simple carbohydrate polymer, consisting of repeating glucose units linked by β-1,4-glycosidic bonds. It is comprised of nanometer-thick crystalline microfibrils and highly resistant to enzymatic hydrolysis [1]. Cellulolytic fungi and bacteria have developed complex cellulase systems that efficiently hydrolyze cellulose [2]. These cellulase systems play important roles in a wide range of processes ranging from biosphere maintenance (carbon recycling) to the generation of potentially sustainable energy sources such as glucose, ethanol, hydrogen, and methane [1], [3]–[5].

For many herbivorous, detritivorous, as well as omnivorous insects, cellulose comprises a major nutritional resource. However, endogenous cellulases were long thought to be absent in metazoans including insects. It had been wildly accepted that cellulose digestion in insects was mediated by gut-associated microbes such as mixtures of bacteria and protozoa under anaerobic conditions [6]–[8]. However, since the first endogenous cellulase gene was identified in the termite *Reticulitermes speratus* [9], many studies now account for the endogenous origin of cellulases in nematodes, insects, and some other invertebrates [10]–[12]. In the termite systems, where the metazoan cellulose digestion is most extensively studied, a dual (independent) or synergistic collaboration system among host and symbiont-mediated cellulases has been proposed [13]–[18]. However, understanding of the exact roles of the host and symbiotic microbiota in the complex cellulose degradation process is still emerging.

Cellulase is a general term for cellulytic enzymes including three classes of hydrolytic enzymes: endoglucanases (EC 3.2.1.4), exoglucanases (cellobiohydrolases: EC 3.2.1.74 and 3.2.1.91), and β-glucosidases (cellobiases: EC 3.2.1.21). Plant cell wall digestion also requires other enzymes including pectinases and hemicellulases. All these enzymes are grouped into glycoside hydrolase (GH; EC 3.2.1.-) (also known as glycosidase or glycosyl hydrolase) families according to their amino-acid sequence similarities and their folding patterns based on the Carbohydrate-Active enZymes Database (CAZy, http://www.cazy.org) [19]. β-glucosidases (GH family 1) are found universally in all domains of organisms [20]. While insects lack endogenous exoglucanases ([11] but see, *e.g.*, [21]), genes encoding endoglucanases and other GH family enzymes have been identified from a number of phytophagous coleopterans belonging to the superfamilies Chrysomeloidea, which includes long-horned beetles and leaf beetles, and Curculionoidea (weevils) [11], [22]–[24]. A β-1,4-endoglucanase gene belonging to the GH family 9 was also isolated and characterized from the red flour beetle *Tribolium castaneum* (Coleoptera: Tenebrionidae) [25].

Recently, we have cloned and characterized a novel β-1,4-endoglucanase gene (*DvvENGaseI*, JQ755253) belonging to the GH family 45 from the western corn rootworm *Diabrotica virgifera virgifera* (Coleoptera: Chrysomelidae), an important insect pest of maize (*Zea mays* L.) in the United States [26], [27]. We showed that suppression of *DvvENGaseI* expression by RNA interference (RNAi) resulted in only slight developmental delays suggesting that this gene might be a part of the larger system of cellulose degrading enzymes [26]. The goal of this study is focused on the exploration of genetic diversity among GH family genes in *D*. *v*. *virgifera*, especially focusing on its larval stages. In order to identify the diversity of GH family genes encoding plant cell wall degrading and related enzymes expressed in *D. v. virgifera* larvae, we sequenced the transcriptomes covering three different developmental stages (eggs, neonates, and midgut from third instar larvae) using next-generation technologies. We identified eight types of GH family genes that encode β-1,4-endoglucanases (GH45, GH48, and GH5) as well as a pectinase (GH28), an endo-1,3-β-glucanase (GH16), an α-galactosidase (GH27), an α-glucosidase (GH31), and a β-glucosidase (GH1). We found large numbers of GH45 and GH28 genes from the *D*. *v*. *virgifera* transcriptomes, among the largest so far known from coleopteran species studied. Our analyses also suggested multiple horizontal transfer events during the evolution of GH45, GH48, and GH28 genes from bacteria or fungi to the common ancestor of chrysomelid and curculionid beetles as well as to other herbivorous insects. Acquisition and subsequent expansion of GH gene copies in phytophagous beetle lineages may have been adaptive and have played important roles for their specialization in feeding on particular host plants.

## Results and Discussion

### Sequencing and *de novo* Assembly of *D. v. virgifera* Transcriptomes

Using Illumina paired-end as well as 454 Titanium sequencing technologies, in total ∼700 gigabases were sequenced from cDNA prepared from eggs (15,162,017 Illumina paired-end reads after filtering), neonates (721,697,288 Illumina paired-end reads after filtering), and midguts of third instar larvae (44,852,488 Illumina paired-end reads and 415,742 Roche 454 reads, both after filtering) (see Table S1 for details). *De novo* transcriptome assembly was performed using Trinity [28] for each of three samples as well as for the pooled dataset (see Materials and Methods and Tables S1, S2, and S3 for the comparative analysis of assembly programs and other details). The *D. v. virgifera* transcriptome assembled from the pooled dataset included 163,871 contigs (the average length: 914 bp) (Table 1).

**Table 1:** Summary of the *D*. *v*. *virgifera* transcriptome assembly using the pooled dataset.

Samples	Egg, neonate, and third-instar larval midgut
Number of paired-end reads before filtering	1,462.2×10^6^ (144,690×10^6^ bp)
Number of paired-end reads after filtering	781.7×10^6^ (77,393×10^6^ bp)
Assembly program used	Trinity (2013-02-25)
Total number of contigs	163,871
Average contig length (range)	914 bp (201–31,064 bp)
N50 length	1,396 bp

### Identification of GH Family Genes from *D*. *v*. *virgifera* Transcriptomes

**Figure 1:**
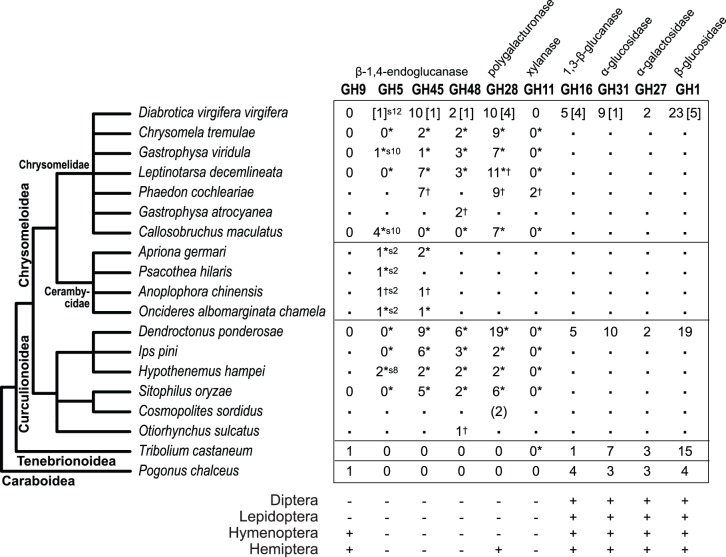
Distribution of glycoside hydrolase family genes among polyphagan coleopterans. Numbers for GH5, GH45, GH48, GH28, and GH11 genes are taken from [22] (marked with *). Exceptions are for *D. v. virgifera* (this study; numbers in square brackets are for partial sequences), *P. cochleariae* GH45, GH28 [83], and GH11 [24], *G. atrocyanea* GH48 [39], *D. ponderosae* [45], *C. sordidus* (preliminary results from transcriptomes are shown in parentheses; A. Valencia-Jiménez, personal communication), *O. sulcatus* GH48 (CAH25542.1), *T. castaneum* [25], [70], and *P. chalceus* (this study, searched from the transcriptome [69]). Numbers with † indicate that they are based on the search results from the NCBI NR database or from literatures. Since neither genomes nor transcriptomes are available for these species, the actual numbers of their GH family genes are not known. For GH5 genes, their subfamilies are indicated with ‘s’ followed by the number (*e.g.*, s2 for subfamily 2). Accession numbers for all coleopteran GH genes included in this study are found in Table S5. The taxonomical relationship is based on [68]. ‘.’: not determined. For other insect groups, only existence (+) or absence (−) is shown.

A total of seventy eight potential genes belonging to eight GH families were identified from our *D*. *v*. *virgifera* transcriptome. In Figure 1, numbers of the genes for these GH families found in *D*. *v*. *virgifera* are compared with those found in other coleopteran species. While the enzymes encoded by GH45, GH48, and GH5 family genes are known to have β-1,4-endoglucanase (EC. 3.2.1.4) activity, GH28 genes encode a pectolytic enzyme, polygalacturonase (EC 3.2.1.15) [19]. GH16 family genes encode a laminarinase, β-1,3-glucanase (EC 3.2.1.39). We also found genes encoding GH27 (α-galactosidase, EC 3.2.1.22), GH31 (α-glucosidase, EC 3.2.1.20), and GH1 (β-glucosidase, EC 3.2.1.21) families.

### GH45 Family

Eleven GH45 family gene candidates were identified from the *D*. *v*. *virgifera* transcriptome with ten of them covering the entire coding regions (615–741 bp or 204–246 amino acids (AA); Figure S1A). The partial sequence (GH45-6) was also confirmed in the draft *D*. *v*. *virgifera* genome. Four of them (GH45-1, GH45-4, GH45-7, and GH45-10) were highly expressed (>100 reads per kilobase of per million mapped reads or RPKM in the neonate and third-instar larval midgut samples but not expressed in the egg samples (Table S4). We have previously identified GH45-7 as *DvvENGaseI* (JQ755253) [26]. This gene exhibits the highest expression among the eleven GH45 family genes and also the highest among all GH genes identified in the present study (Table S4). Note that its gene and protein expressions in *D. v. virgifera* larvae were also confirmed in our previous study [26].

GH45 family genes have been described from a number of coleopteran species belonging to the suborder Polyphaga (*e.g.*, [23], [29]–[31]). Similarity searches against the NCBI (National Center for Biotechnology and Information) non-redundant (NR) protein database as well as ten complete insect genomes confirmed that within insects, GH45 family genes are found only in two polyphagan coleopteran superfamilies, Chrysomeloidea and Curculionoidea. As shown in Figure 1, multiple GH45 genes have been identified in some species. *D*. *v*. *virgifera* has the largest known number of GH45 genes (11 genes) among coleopteran species, and probably among any known invertebrates where this gene exists.

**Figure 2:**
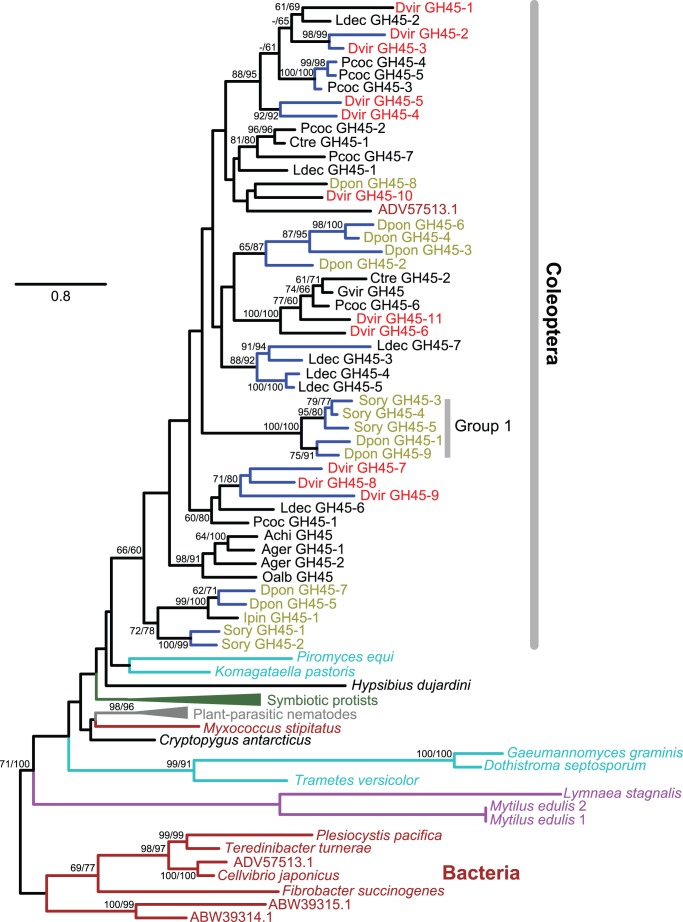
The maximum-likelihood phylogeny of GH45 proteins. Forty seven GH45 protein sequences from eleven coleopteran species are included. Their species name abbreviations are found in Table S5. Labels for the coleopteran species belonging to the superfamily Curculionoidea are olive-colored and all other coleopteran sequences colored in black belong to the superfamily Chrysomeloidea. *D*. *v*. *virgifera* sequences are shown in red. Other sequences include: two mollusks (purple), *Cryptopygus antarcticus* (Collembola, black), *Hypsibius dujardini* (Tardigrada, black), 24 termite-symbiotic protists (dark green), 10 plant-parasitic nematodes (all are from *Bursaphelenchus xylophilus,* grey), representative fungi (chosen from 138 sequences, cyan), and representative bacteria (chosen from 18 sequences, brown). Bacterial sequences were used as outgroups. The numbers at internal branches show the bootstrap support values (%) for the maximum-likelihood and neighbor-joining phylogenies in this order. Supporting values are shown only when higher than 60%. Blue-colored branches indicate the species-specific gene duplications (based on currently available sequences) within a cluster supported by higher than 70% of bootstrap values. The scale bar represents the number of amino acid substitutions per site. See Figure S2 for more details.

In addition to these coleopteran sequences, a sequence similar to the GH45 family has been identified from the Antarctic springtail *Cryptopygus antarcticus* [12], which belongs to one of the basal hexapodan orders, Collembola [32]. Another sequence similar to the GH45 family was reported from the water bear *Hypsibius dujardini* (phylum Tardigrada, a sister group of arthropods) [33]. GH45 family genes have also been reported among various metazoans, from protists symbiotic to wood-feeding termites and a cockroach [34], [35] to plant-parasitic nematodes and mollusks [10], [36]–[38]. In order to understand the evolutionary process that has led to the diversity of coleopteran GH45 family genes, a maximum-likelihood phylogeny was reconstructed including GH45 family proteins from eleven coleopteran species as well as other metazoans mentioned above, fungi, and bacteria (Figure 2). Our phylogenetic analysis suggests that all coleopteran GH45 family genes are monophyletic although the support was weak (≤66% bootstrap supports). Based on currently available sequences, several species-specific gene duplications were found in coleopteran species (shown with blue branches in Figure 2). While all bacterial GH45 family proteins, except for sequences from *Myxococcus stipitatus* and uncultured bacterium (ADV57513.1), formed a monophyletic group, relationships among fungal and metazoan sequences were unresolved. Although the exact timings are not clear, multiple horizontal gene transfer (HGT) events are likely to have involved in the evolution of metazoan GH45 family genes.

As shown in [22], except for a clade of curculionid proteins (Group 1 in Figure 2), the putative catalytic nucleophile and proton donor positions of GH45 proteins are highly conserved with Asp (Gln is found in Group 1 proteins). This is also the case for all but one *D*. *v*. *virgifera* GH45 proteins (Val is found in GH45-9; Figure S1A). Other exceptional cases include: Asn in *H*. *dujardini* (Tardigrada), Thr in *Leptosphaeria maculans* (fungus), Ser in *Alternaria alternate* (fungus), and Glu in *Myxococcus stipitatus* (bacterium). In addition to possessing the conserved catalytic residues, the majority of GH45 genes identified in *D. v. virgifera* showed significant expression levels in neonate and third-larval midgut transciptomes (Table S4). These proteins probably share similar functions and help explain our previous results with RNAi-suppression of single GH45 gene expression not drastically affecting the *D. v. virgifera* larval development.

### GH48 Family

We identified three GH48 family gene candidates from *D*. *v*. *virgifera*: two complete (1,926 bp, 641 aa) and one partial (374 bp, 124 aa) (Figure S1B). The partial sequence (GH48-2) was confirmed in the draft *D*. *v*. *virgifera* genome. Similar to GH45 family genes, GH48 family genes have been identified from many polyphagan coleopterans especially from the two superfamilies (Chrysomeloidea and Curculionoidea) [22], [39], [40] (Figure 1). Consistent with the results shown in [22], the number of GH48 genes found in coleopterans was in general smaller than those of GH45 and GH28 family genes.

**Figure 3:**
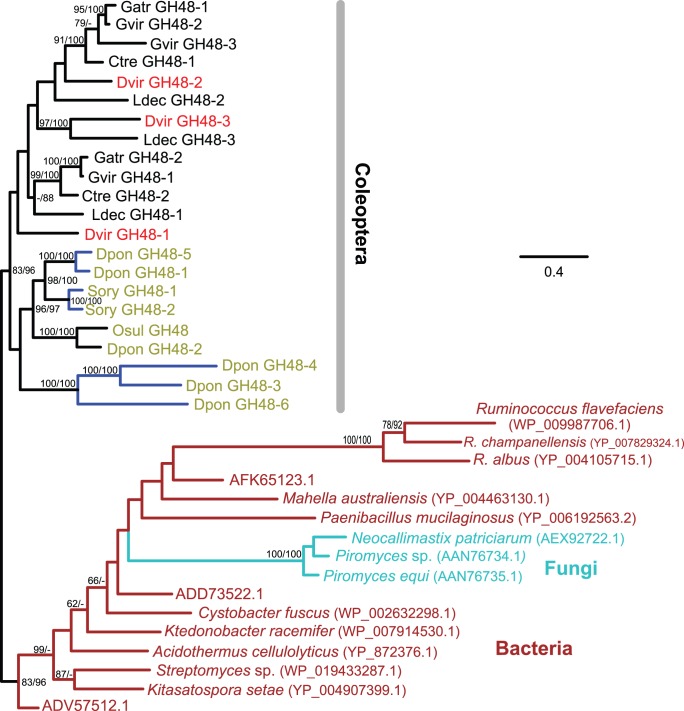
The maximum-likelihood phylogeny of GH48 proteins. Twenty two GH48 protein sequences from seven coleopteran species are included. Their species name abbreviations are found in Table S5. Labels for the coleopteran species belonging to the superfamily Curculionoidea are olive-colored and all other coleopteran sequences colored in black belong to the superfamily Chrysomeloidea. *D*. *v*. *virgifera* sequences are shown in red. Other sequences include: representative bacteria (chosen from 653 sequences, brown) and 3 fungi (shown in cyan). Bacterial sequences were used as outgroups. The numbers at internal branches show the bootstrap support values (%) for the maximum-likelihood and neighbor-joining phylogenies in this order. Supporting values are shown only when higher than 60%. Blue-colored branches indicate the species-specific gene duplications (based on currently available sequences) within a cluster supported by higher than 70% of bootstrap values. The scale bar represents the number of amino acid substitutions per site.

Two GH48 family genes (active phase-associated proteins, APAP I and II; shown as Gatr GH48-1 and −2 in Figure 3) were isolated from a leaf beetle *Gastrophysa atrocyanea* [39]. While neither glucanase nor cellobiohydrolase activity was detected with *G*. *atrocyanea* GH48-1, it exhibited chitinase activity. *G*. *atrocyanea* GH48-1 was shown to be necessary for diapause termination in adults [39]. Based on our phylogenetic analysis, *G*. *atrocyanea* GH48-1 was found to be closer to *D*. *v*. *virgifera* GH48-2 (Figure 3). However, only a fragment has been identified from the *D*. *v*. *virgifera* GH48-2 and its expression was not confirmed from our egg and larval samples (Table S4). While *D. v. virgifera* GH48-1 also had very low expression, GH48-3 was found to be expressed more in larvae than in eggs.

GH48 is one of the most common GH family genes in bacteria [41]. Apart from their presence in bacteria and in coleopterans, this family has been reported from three fungal species (*Neocallimastix patriciarum*, *Piromyces equi*, and *Piromyces* sp.). None of the ten insect genomes we examined had GH48 family genes. This disparate and limited distribution of GH48 family genes in two related coleopteran superfamilies and in three fungal species but not in any other eukaryotes, clearly indicates at least two independent HGT events: one from bacteria to the ancestral coleopteran lineage before the divergence of the two coleopteran superfamilies and the other from bacteria to the ancestral lineage before the divergence of the three fungal species. The three fungal GH48 sequences belong to the family Neocallimastigaceae (phylum Neocallimastigomycota). These fungi are isolated in the digestive tracts of ruminant and non-ruminant mammals and herbivorous reptiles [42]. Although our similarity search and phylogenetic analysis did not show a clear relationship with any known bacterial species, rumen fungi have been reported to obtain catalytic enzymes from bacterial sources by HGT events. For example, GH5 (endoglucanase, EC 3.2.1.4) and GH11 (xylanase, EC 3.2.1.8) family genes found in *Orpinomyces joyonii* and *Orpinomyces* sp. (phylum Neocallimastigomycota) are considered to be bacterial origin [43]. GH5 family genes in a rumen fungus, *Neocallimastix patriciarum*, have also been suggested to have originated from bacteria (*Streptococcus equinus* and *Ruminococcus albus*) [44].

### GH28 Family

**Figure 4:**
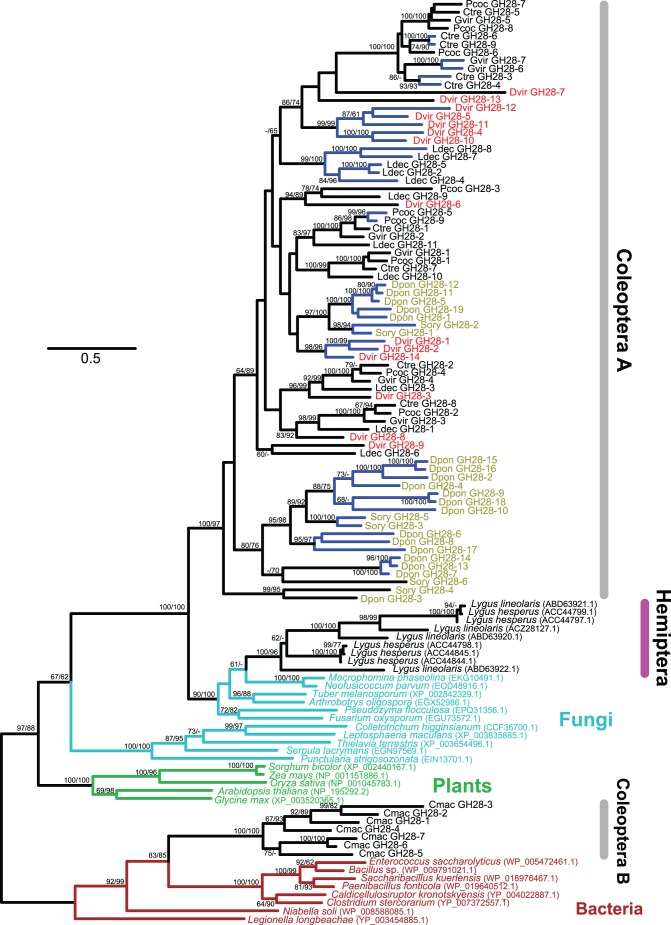
The maximum-likelihood phylogeny of GH28 proteins. Eighty four GH28 protein sequences from eight coleopteran species are included. Their species abbreviations are found in Table S5. Labels for the coleopteran species belonging to the superfamily Curculionoidea are olive-colored and all other coleopteran sequences colored in black belong to the superfamily Chrysomeloidea. *D*. *v*. *virgifera* sequences are shown in red. Other sequences include: plant bugs (*Lygus hesperus* and *Lygus lineolaris*), representative fungi (chosen from 651 sequences, cyan), representative bacteria (chosen from 42 sequences, brown), and representative plants (chosen from 491 sequences, green). Bacterial sequences were used as outgroups. The numbers at internal branches show the bootstrap support values (%) for the maximum-likelihood and neighbor-joining phylogenies in this order. Supporting values are shown only when higher than 60%. Blue-colored branches indicate the species-specific gene duplications (based on currently available sequences) within a cluster supported by higher than 70% of bootstrap values. The scale bar represents the number of amino acid substitutions per site.

GH28 family genes encode polygalacturonase (pectinase, EC 3.2.1.15). Ten intact (average 1087 bp, 361 aa) and four partial GH28 candidate sequences were identified in the *D*. *v*. *virgifera* transcriptome (Figure S1C). Gene expression, especially in larvae, was confirmed from the majority of the eleven intact candidates (Table S4). Although the expression of the three partial sequences (GH28-8, 10, and 14) was either very low or confirmed neither in eggs nor in larvae, their partial sequences were found in the draft genome. Multiple copies of GH28 family genes have been found in a number of coleopteran species belonging to its two superfamilies (Chrysomeloidea and Curculionoidea) [22], [29]. The largest number (19 functional genes) was found in the mountain pine beetle (*Dendroctonus ponderosae*) [40], [45]. *D*. *v*. *virgifera* has the second largest number, 10 (and 4 partial sequences), of GH28 family genes (Figure 1). Our phylogenetic analysis based on currently available sequences confirmed many species-specific duplications of GH28 family genes in coleopterans (Figure 4, blue branches).

Consistent with what was indicated by Pauchet *et al.* [22], our phylogenetic analysis showed that GH28 family genes can be divided into two clades. GH28 enzymes from *Callosobruchus maculatus* (bean beetle) form a subgroup (B, Figure 4) and are more closely related to bacterial GH28 enzymes (all Gram-negative bacteria) (>83% bootstrap supports), while all other beetle GH28 enzymes are more closely related to fungal and plant bug (Hemiptera) enzymes. Although two plant bug species (*Lygus hesperus* and *Lygus lineolaris*, Hemiptera) were reported to have multiple GH28 family genes [46], [47], we failed to identify GH28 candidate sequences in the ten insect genomes including two from hemipterans *Rhodnius prolixus* (a blood-sucking bug) and *Acyrthosiphon pisum* (pea aphid). Among insects, except for the two plant bug species, GH28 family genes were found only in two coleopteran superfamilies (Chrysomeloidea and Curculionoidea). These insect GH28 family genes except for those of *C. maculatus* are phylogenetically nested within a fungal GH28 cluster (Figure 4). Therefore, GH28 genes currently found in coleopterans and plant bugs were most likely acquired by three independent HGT events: from Gram-negative bacteria to *C. maculatus*, from a fungus to a hemiptera, and from a fungus to an ancestral coleopteran before the divergence of the two superfamilies.

### GH16 Family

We identified nine GH16 family genes, which encode β-1,3-glucanases, in the *D*. *v*. *virgifera* transcriptome: five full-length (average 1124 bp, 374 aa) and four partial coding sequences (Figure S3A). Their expressions were identified both in larval and egg samples (Table S4). The most significantly highly expressed gene (GH16-1) showed larval specific expression. GH16 family genes are widely found in insects (*e.g.*, [48]–[50]). Similarity searches further confirmed a wide distribution of GH16 family genes within metazoa including insects, mollusks (*e.g.*, [51]), sea urchins (*e.g.*, *Strongylocentrotus purpuratus*), as well as basal chordates (*e.g.*, *Ciona intestinalis*) but not in vertebrates. It was also found widely in fungi and bacteria. An ortholog identified in the Antarctic springtail *C*. *antarcticus* (CaLam) is believed to have originated from bacteria by HGT [52].

**Figure 5:**
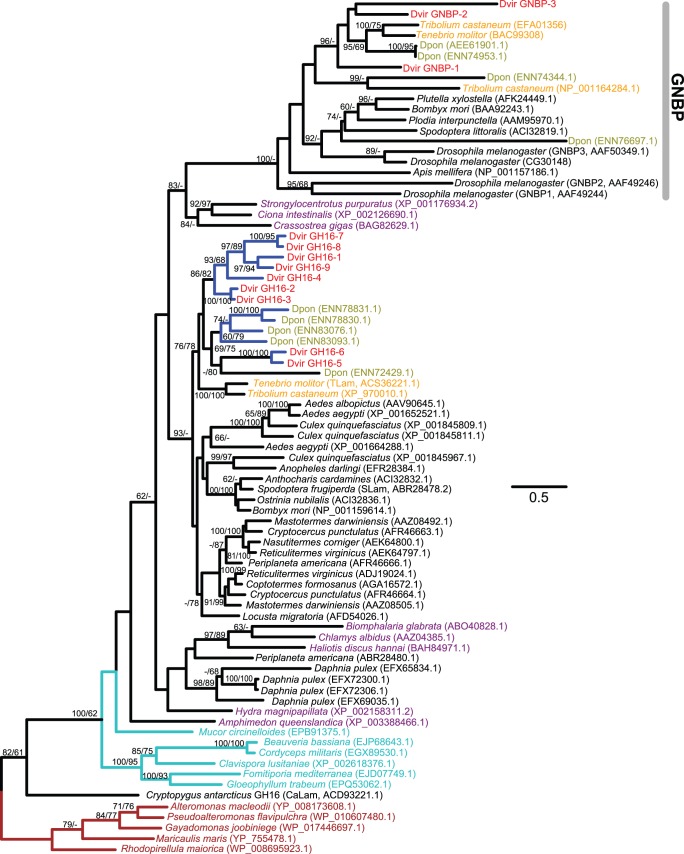
The maximum-likelihood phylogeny of GH16 proteins. Sixteen GH16 protein sequences from four coleopteran species are included. Labels for the coleopteran species belonging to the superfamily Curculionoidea, *D*. *v*. *virgifera*, and other beetle sequences are shown in olive, red, and orange, respectively. Their species abbreviations are found in Table S5. Arthropod, other metazoan, fungal (6 chosen from 222 sequences), and bacterial (5 chosen from 977 sequences) sequences are indicated by black, purple, cyan, and brown, respectively. Bacterial sequences were used as outgroups. The numbers at internal branches show the bootstrap support values (%) for the maximum-likelihood and neighbor-joining phylogenies in this order. Supporting values are shown only when higher than 60%. Blue-colored branches indicate the species-specific gene duplications (based on currently available sequences) within a cluster supported by higher than 70% of bootstrap values. The scale bar represents the number of amino acid substitutions per site.

Figure 5 shows the phylogeny of GH16 family protein sequences from four coleopteran species (*T. castaneum*, *Tenebrio molitor*, *D*. *ponderosae*, and *D*. *v*. *virgifera*) and other insects as well as some other metazoans, fungi, and bacteria. As reported previously, insects have a group of pattern recognition proteins that are originated from a duplicated copy of GH16 family genes [48]–[50]. They are called Gram-negative bacteria-binding proteins (GNBPs) or β-1,3-glucan recognition proteins (βGRPs) and are involved in innate immune recognition [53]–[57]. These proteins (indicated as “GNBP” in Figure 5) have lost their original GH16 enzymatic activity [56], [58] and their active sites are not conserved (Figure S3B; also see [48], [49]). These proteins, however, contain a unique conserved β-1,3-glucan binding domain in their N-terminal regions [57], [59], [60]. Both types of genes, GH16 family genes with conserved active sites as well as GNBP genes that contain the N-terminal domain but no conserved active sites, have been identified from the coleopteran species examined so far (*T. castaneum, T. molitor, D. ponderosae*). Both types of genes were also identified in our *D. v. virgifera* transcriptome. For the nine GH16 family gene candidates, the active site regions show highly conserved patterns including two Glu residues (Figure S3A). We also identified three potential GNBP genes from *D. v. virgifera*. Consistent with GNBPs found in other insects, their active sites are not conserved with Glu’s (Figure S3B). All but one GNBP gene candidates were weakly expressed both in eggs and in larvae (neonates and third-instar larval midguts) (Table S4), which is consistent with the pattern found with *Drosophila* GNBP genes [53]. One gene (GNBP-3) does not have the conserved N-terminal domain (Figure S3C); this gene may not function as a GNBP. No expression was detected from this gene in larval samples.

The β-1,3-glucanase activity has been confirmed with GH16 enzymes identified from midguts of several insects including *T*. *molitor* (TLam) [61], *Spodoptera frugiperda* (Lepidoptera) (SLam) [49], *Helicoverpa armigera* (Lepidoptera) [48], *Abracris flavolineata* (Orthoptera) [62], *Periplaneta americana* (Blattodea) [63], as well as termites (Isoptera) [58]. These insect, except for lepidopteran, enzymes can lyse *Saccharomyces cerevisea* cells. For detritivorous insects such as *T. molitor*, *P. americana*, and termites, their abilities of digesting fungal cell walls may play roles in antifungal protection as well as in nutrient acquisition. *T. molitor*’s midgut content is found to be almost free from fungi [61]. Blocking one of these proteins in termites accelerated and increased fungal infection [58]. β-1,3-glucanase can also hydrolyze callose (β-1,3-glucan). Callose exists in the cell walls of higher plants and plays important roles during plant development. Callose deposition also is induced by a variety of biotic and abiotic stresses such as wounding, pathogen infection, aluminum, abscisic acid, and raised or lowered temperatures [64]. Therefore, it is plausible that *D. v. virgifera* larvae use this enzyme (*e.g.*, encoded by GH16-1, which has significantly high larval expression) to digest callose.

### GH5 Family

A short sequence similar to part of GH5 family genes was identified in the *D*. *v*. *virgifera* transcriptome (317 bp, 105 aa) (Figure S4A). Among the 51 GH5 subfamilies [65], coleopteran GH5 genes known so far belong to three subfamilies (2, 8, and 10) (Figure S4B). The subfamily 8 gene found in the coffee berry borer *Hypothenemus hampei* (Curculionoidea), however, was shown to be bacterial origin [66]. The short *D*. *v*. *virgifera* GH5 sequence is phylogenetically closer to fungal GH5 sequences belonging to the subfamily 12 (Figure S4B). We should, however, note that we failed to confirm the corresponding sequence in the draft *D*. *v*. *virgifera* genome. Furthermore, the expression of this sequence was not confirmed with confidence (Table S4). Therefore, we consider the existence of a GH5 gene in *D*. *v*. *virgifera* to be inconclusive.

### Absence of GH9 Family in *D. v. virgifera*

GH9 family genes have been identified in insect orders Orthoptera, Blattaria, Phthiraptera, Hemiptera, Coleoptera, and Hymenoptera [11], [67]. However, no GH9 candidate sequence was identified in the *D*. *v*. *virgifera* transcriptome (Figure 1). GH9 family genes appear to be absent among chrysomelids and curculionids. [11], [67]. Among beetle species, a GH9 family gene is present in *T*. *castaneum* (Tenebrionoidea) [25]. We also found a GH9 family gene sequence from the transcriptome of the salt marsh beetle *Pogonus chalceus* (Caraboidea, Adephaga). Because *P*. *chalceus* is placed as the most basal species in Coleoptera [68] (Figure 1), GH9 family genes were likely maintained in the common ancestor of Coleoptera and the lineage leading to the superfamily Tenebrionoidea. GH9 family genes must have been subsequently lost in the common ancestor of Chrysomeloidea and Curculionoidea.

We confirmed that three GH families (GH45, GH48, and GH28) are absent from the transcriptomes of *P*. *chalceus* [69] and the genome of *T*. *castaneum* [70] (Figure 1). The loss of GH9 and gain of GH45, GH48, and GH28 families, therefore, can be traced back at least to the common ancestor of chrysomelids and curculionids. Although GH9 and three enzymes (GH48, GH45, and GH28) do not share sequence similarities and have different 3D structural features (CAZy classifies GH48 in GH-M and GH28 in GH-N clans; GH9 and GH45 are not classified), Watanabe and Tokuda [11] suggested, for example, a possible convergent evolution in terms of enzymatic function based on the same substrate specificities (*e*.*g*., β-1,4 linkages) with GH9 and GH45 enzymes. GH9 and GH28 enzymes utilize the inverting glycosidase mechanism, which only allows polysaccharide hydrolysis [71]. Thus, their functional similarities may have allowed the laterally acquired genes to replace the role of the lost GH9 enzymes.

### GH1 Family

The hydrolysis of cellulose is completed by β-glucosidases, which hydrolyze cellobiose and oligosaccharides to glucose. The GH1 family, the largest of GH families that encode β-glucosidase activities, has been identified widely in insects (*e.g.*, [72]–[74]). From the *D*. *v*. *virgifera* transcriptome, we identified twenty-eight GH1 gene candidates (23 intact and 5 partial; Figure S5A). Multiple copies of GH1 gene candidates were found also in other coleopteran species (*e.g.*, 19 in *D. ponderosae* and 15 in *T. castaneum*) (Figure 1). While multiple GH1 family genes are regularly found in eukaryotic organisms, this family is particularly expanded in coleopteran species. Phylogenetic analysis shows that all insect GH1 family proteins are monophyletic (although bootstrap support is marginal; 76% only by the maximum-likelihood phylogeny) (Figure S5B). Consistent with previous studies, GH1 family proteins form clusters according to each domain of life [75], [76]. GH1 family expansion appears to have happened independently in many different lineages. For example, four β-glucosidases have been identified in the midgut of the yellow mealworm *T. molitor*, and their enzyme specificities and efficiencies differ slightly [72], [77]. The majority of GH1 gene candidates we identified from *D. v. virgifera* have high expression in larvae, especially in the third-instar larval midgut, but not in eggs (Table S4). Therefore, these GH1 gene products likely participate in digesting various plant materials.

### GH31 and GH27 Families

In addition to cellulases and other plant cell wall digesting enzymes, we also searched genes encoding GH31 (α-glucosidase) and GH27 (α-galactosidase) families. These enzymes share a common (β/α)_8_ (TIM) barrel catalytic domain and belong to the same GH-D clan [78]. In mosquitoes, an α-glucosidase has been shown to function as a receptor of Bin toxin from *Bacillus sphaericus* as well as Cry11Ba toxin from *B. thuringiensis* subsp. *jegathesan* [79], [80]. Ten GH31 and two GH27 family gene candidates were identified from the *D*. *v*. *virgifera* transcriptome (Figures S6A and S7A). All of these genes are highly expressed especially in the third-instar larval midgut (Table S4). Both families are found in a wide range of organisms from bacteria to eukaryotes (Figures S6B and S7B). Wheeler *et al.* [81] showed two lepidopteran GH31-related sequences as HGT origin from bacteria (BGIBMGA013995 and Px016165 in Figure S6B). We found no such evidence in search of GH31 family genes in *D. v. virgifera* as well as in *D. ponderosae* transcriptomes.

### GH Family Gene Expression

We compared the expression levels of GH family gene candidates we identified from the *D*. *v*. *virgifera* transcriptomes between egg and larval samples. Almost all were expressed significantly more in larval stages. We found that the majority of GH45, GH28, GH1, GH31, and GH27 family genes are expressed more in the third-instar larval midgut samples compared to egg and neonate samples, with some genes particularly standing out (GH45-4, GH45-7, GH45-10, GH28-6, GH1-18, GH27-1, GH31-7) (Table S4). Gene expression and enzyme activity of polygalacturonase have been reported from the gut of another corn rootworm species, *Diabrotica undecimpunctata howardi* (spotted cucumber beetle) [82]. GH28 and GH45 family genes are expressed more in the guts of *P*. *cochleariae* larvae and adults [83]. Polygalacturonases are known to loosen the primary cell wall and make cellulose-hemicellulose network more accessible to enzymatic digestion [84]. With its high number of GH45, GH28, and GH1 genes and their high expression in larval midgut tissue, *D*. *v*. *virgifera* may utilize β-1,4-endoglucanase, polygalacturonase, as well as β-glucosidase activities in larval midgut to assist in the digestion of corn root cell walls and in releasing dietary monosaccharides such as glucose.

### Horizontal Gene Transfer of GH Family Genes

Our current study indicates that the three GH gene families (GH45, GH48, and GH28) are unique to the two coleopteran superfamilies (Chrysomeloidea and Curculionoidea) and generally absent from other insects except in plant bugs (GH28) and in a springtail (GH45). These results imply that these genes are likely not vertically inherited from the ancestral species but acquired by HGT events from bacteria or fungi to the common ancestor of chrysomelid and curculionid beetles.

As mentioned before, the GH5 family gene (*HhMAN1*) identified from the coffee berry borer *H. hampei* is thought to be bacterial origin [66]. This gene was not found in two other related species, *H*. *obscurus* (topical nut borer) and *Araecerus fasciculatus* (coffee bean weevil, Anthribidae, Coleoptera). *H*. *obscurus* is a pest of macadamia nuts but not coffee [85]. *A. fasciculatus* is polyphagous, a common pest of stored food products including coffee [86]. In contrast, *H. hampei* is mainly a coffee pest, although it may not be strictly monophagous [87], [88]. Therefore, acquisition of *HhMAN1* from bacteria may have made a rapid adaptation possible for *H. hampei* by enabling hydrolysis of galactomannan, the major nutrient source for this species [66]. Other examples of possible HGTs of GH family genes include: GH5 and GH11 family genes in rumen fungi from rumen bacteria *Fibrobacter succinogenes* [43], a GH16 family gene in *C*. *antarcticus* from bacteria [52], lepidopteran GH31-like genes from *Enterococcus* bacteria [81], and GH11 family genes in *P*. *cochleariae* from γ-proteobacteria [24]. We also found evidence of several independent HGT events such as fungal GH48 family genes and plant bug GH28 family genes. Although HGT events are often detected in prokaryotes [89], GH families seem to be characterized by frequent HGT events in various animals especially in insects. Such acquisitions followed by frequent duplications of these GH genes must have contributed to these organisms’ ability to adapt to novel niches.

## Conclusion

We have identified eight GH family genes from the transcriptomes of *D*. *v*. *virgifera*. Three GH families (GH45, GH48, and GH28) were likely to have been obtained by HGT events before the divergence of chrysomelid and curculionid beetles. Rapid birth-and-death processes have been also observed among these coleopteran GH family genes. A large number of GH family enzymes owing to their lineage-specific duplications in *D*. *v*. *virgifera* could have contributed to the successful adaptation to its niche by providing more efficient hydrolyzation of corn cell walls.

## Materials and Methods

### Sample Collection and Preparation

#### Eggs

Two thousands freshly hatched non-diapause *D. v. virgifera* eggs (ten Petri dishes, ∼200 eggs/Petri dish) were purchased from Crop Characteristics, Inc. (Farmington, Minnesota, USA). All ten Petri dishes were wrapped with aluminum foil and placed in a growth chamber for incubation at 27°C. One Petri dish was removed from incubator on each day and the eggs were isolated with a 60-mesh sieve. Briefly, the soil with eggs were rinsed with tap water until soil was removed completely, and the isolated eggs were washed with double distilled water before being transferred into a 1.7 ml centrifuge tube. The water was removed with pipette and eggs were snap-frozen in liquid nitrogen and stored in −80°C freezer. All other Petri dishes were processed in the same way until the day 10.

#### Neonates

A Petri dish containing 10,000 eggs was purchased from Crop Characteristics, Inc. (Farmington, Minnesota, USA) and placed in a growth chamber at 27°C with LD 16∶8 photoperiod until hatching. The eggs were isolated from soil with methods described above. The clean eggs were rinsed with double distilled water three times before transferring to a new egg Petri dish (60×15 mm) with moistened filter paper (42.5 mm). Finally, the Petri dish was placed back to the same growth chamber for more neonates to hatch. The freshly hatched (less than 24 hrs old) neonates were collected, snap-frozen in liquid nitrogen, and stored at −80°C freezer.

#### Preparation of midgut from third instar larvae

Fifty third-instar larvae purchased from Crop Characteristics, Inc. (Farmington, Minnesota, USA) were dissected for midgut tissue under dissection microscope. Briefly, the head, thorax, and last two segments of abdomen were removed with a scalpel and the midgut was pulled from the carcass with forceps. The fat body and other liquids were carefully removed by pulling the midgut on a clean Kimtech Science Precision Wipes Tissue Wipers (Fisher). The midgut was then opened longitudinally and gut contents were removed by rinsing 3 times with 1X PBS buffer (pH 7.4). The clean midgut tissue was snap-frozen in liquid nitrogen and saved at −80°C until RNA extraction. Sixteen midgut tissues were pooled as one replicate with 3 replicates in total.

#### RNA extraction

Approximately 35 mg of pooled midgut tissue was used directly in a single RNA preparation while ∼32 pooled neonates were used for single RNA extraction. For egg RNA preparation, ∼35 mg of pooled eggs from day 1 to day 10 were used in single RNA preparation. Three replicates for eggs and midgut dissection and six replicates for neonates were prepared. The total RNAs were extracted with RNeasy mini kit (Qiagen, Cat. 74104) and treated with RNase-free DNase set (Qiagen, Cat. 79254) for potential genomic DNA contamination by following the manufactures’ instructions. The quality and quantity of RNA were evaluated on 1% agarose gel and NanoDrop 1000 (Thermo) for further analysis.

### Next Generation Sequencing and Assembly of *D. v. virgifera* Transcriptomes

#### Next generation sequencing

The 454 pyrosequencing experiments of larval midgut samples were completed using Roche GS-FLX titanium sequencer at the Core for Applied Genomics and Ecology, University of Nebraska-Lincoln. The transcriptome sequencing for the egg and larval midgut samples with an insert size of 300 bp was done on Illumina Genome Analyzer II platform at the Center for Biotechnology, University of Nebraska-Lincoln. The neonate samples were sequenced with an insert size of 500 bp on Illumina HiSeq2000 system at the Durham Research Center, University of Nebraska Medical Center. In total, 16.6 gigabases (Gb) (read length 75 bp) of egg RNA, 33 Gb (read length 75 bp) of larval midgut RNA, and 662 Gb (read length 101 bp) of neonate RNA were sequenced. All 454 and Illumina reads were deposited into the NCBI Sequence Read Archive (SRA) under the accession number SRP037561.

#### Filtering of low quality reads

Because sequencing errors can cause difficulties for the assembly algorithm, we applied a stringent quality filter process. For 454 reads, the adapter and poly(A/T) sequences were trimmed using PRINSEQ [90]. 454 reads that have abnormal read length (<50 bp or >1000 bp) or where the average quality was less than 20 were removed. The Illumina paired-end reads that did not have the minimum quality score (20 per base for egg and midgut samples or 30 per base for neonate samples) across the whole read were removed using PRINSEQ [90] and Sickle (ver. 1.2) [91]. Note that the quality scores of 20 (Q20) and 30 (Q30) correspond to 1% and 0.1% expected error rates, respectively. We removed all Illumina reads that have any unknown nucleotide ‘N’.

#### *de novo* transcriptome assembly

After the filtering process, we performed *de novo* transcriptome assembly for each of three samples. We used four different short read assemblers: Newbler (ver. 2.5) (Roche, 454 Life Sciences; used only for 454 read assembly), Mira (ver. 3.4.0) [92], Velvet/Oasis (ver. 1.2.03) [93], and Trinity (release 2013-02-25) [28]. The k-mer size of 25 was used for all programs. Mira could be used only for 454 read assembly from the third instar larval samples and for the Illumina read assembly from the egg samples due to the large memory requirement. The results of these assemblies are summarized in Table S1. The number of assembled transcripts varied among the different assemblers, ranging from 37,181 by Trinity to 165,361 for Velvet/Oasis for 454 reads (larval midgut sample) and from 56,135 by Velvet/Oasis to 72,638 by Trinity for Illumina reads (egg sample). The average length and N50 of contigs were generally longer with the Trinity assembly (Tables S1 and S2). Results of NCBI BLAST similarity search (blastx, ver. 2.2.26+) [94], [95] against the UniProt protein database (http://www.uniprot.org) [96] showed that fractions of contigs that had highly significant hits (E-value ≤10^−100^) were larger with the Trinity (18.9%) and Velvet/Oasis assemblies (∼19.4%) than the Mira assembly (11%) although the difference was not significant (*P*>0.5 by *t*-test between Trinity and Mira) (Figure S8). Note that Zhao *et al.* [97] showed the highest accuracy with Trinity among methods specialized in *de novo* transcriptome assemblies such as SOAPdenovo [98], ABySS [99], Velvet/Oasis, and Trinity (they did not include Mira in their comparison). We also attempted the hybrid assemblies using two different sequencing platforms (454 and Illumina Genome Analyzer II) for the third instar larval midgut sample as well as for the pooled egg and third instar larval midgut samples (Table S3). Furthermore, we performed assembly using the dataset pooled from egg (produced by Illumina Genome Analyzer II), third instar larval midgut (produced by Illumina Genome Analyzer II), and neonate samples (produced by Illumina HiSeq2000). Among all of these assemblies, the Trinity assembly using the pooled Illumina dataset had the longest average length of contigs and N50, even longer than the hybrid assemblies including 454 reads (Table 1). With this assembly, more GH family gene candidates were also identified. Therefore, we used this Trinity assembly using the pooled dataset as the most inclusive “combined *D. v. virgifera* transcriptome” for all our further studies.

### Sequence Analysis

#### Gene expression analysis

To compare the gene expression levels, the paired-end reads were mapped onto our combined *D*. *v*. *virgifera* transcriptome using bowtie (ver. 1.0.0) [100] with 0 mismatch. The numerical values of gene expression were measured by RPKM (reads per kilobase per million mapped reads) to normalize for the number of sequencing reads and total read length [101]. RPKM values above 0.3 [102] as well as having more than 10 reads was used as the threshold for gene expression.

#### Identification of GH family genes from the *D. v. virgifera* transcriptome

Previously reported insect, especially coleopteran, GH family gene sequences were obtained for GH45 [22], [23], [31], for GH48 [22], [39], for GH28 [22], for GH9 [25], for GH5 [66], for GH11 [24], for GH16 [61], for GH1 [72], [73], for GH27 (XP_973339.2; this entry is incorrectly shown to be a GH31 family enzyme), and for GH31 [81] (see Table S5). Using these sequences as initial queries, we searched GH family gene candidates against our combined *D*. *v*. *virgifera* transcriptome using NCBI BLAST (tblastn, ver. 2.2.26+) [94], [95]. The initial E-value threshold (1×10^−6^) used was rather lenient and chosen to be inclusive of all true positives even though some false positives from non-target genes may have been included. With recurrent phylogenetic analyses and BLAST (blastp) similarity searches, we identified protein sequences for each GH family. Reciprocal similarity search (using blastp) was further performed against the NCBI NR protein database to confirm GH family associations. All *D*. *v*. *virgifera* GH gene candidate sequences were also confirmed by BLAST (tblastn, E- value ≤10^−30^) similarity search against the draft *D*. *v*. *virgifera* genome sequence (Hugh M. Robertson, personal communication). The criterion we used to identify alternative spliced isoforms was to have a more than 60 bp of 100% identical region among the candidate sequences. We did not find any potential alternative spliced isoforms for the GH gene sequences we identified.

#### GH family gene search

In order to identify GH family sequences from a wide range of insects and other organisms, we performed BLAST similarity searches (blastp, E- value ≤10^−30^) using *D*. *v*. *virgifera* and other representative GH gene sequences as queries against the NCBI NR protein database (http://www.ncbi.nlm.nih.gov) as well as against ten insect genomes (*Drosophila melanogaster*, *Anopheles gambiae*, *Aedes aegypti*, *Bombyx mori*, *Apis mellifera*, *Nasonia vitripennis*, *Solenopsis invicta*, *Ixodes scapularis*, *Rhodnius prolixus*, and *Acyrthosiphon pisum*). For all BLAST similarity searches, in order to obtain comparable E-values, the database size was set to 1.1×10^10^ (using the ‘-dbsize’ option), which is based on the database size equivalent to the NCBI NR database.

#### Multiple sequence alignments and phylogenetic analysis

Multiple alignments of GH family protein sequences were generated using MAFFT (ver. 7.050b) with the L-INS-i algorithm, which uses a consistency-based objective function and local pairwise alignment with affine gap costs [103]. Phylogenetic relationships were reconstructed by the maximum-likelihood method using RAxML (ver. 7.0.4) [104] with the PROTGAMMAJTT substitution model (JTT matrix with gamma-distributed rate variation). The neighbor-joining phylogenies [105] were reconstructed by using neighbor of the Phylip package (ver. 3.69) [106]. The protein distances were estimated using protdist of the Phylip package with the JTT model. Non-parametric bootstrapping with 1000 pseudoreplicates [107] was used to estimate the confidence of branching patterns. FigTree (http://tree.bio.ed.ac.uk/software/figtree) was used to display the phylogenetic trees.

The nucleotide sequences of all GH family genes identified and the alignments used for the phylogenetic analysis in this study are available from our website: http://bioinfolab.unl.edu/emlab/GH/.

## Supporting Information

Figure S1 **GH family gene sequences identified from the** ***D. v. virgifera*** **transcriptome.** Amino acid sequences of GH45 (A), GH48 (B), and GH28 (C) are shown in alignments. The labels for partial sequences are shown in italics. Potential residues for the catalytic nucleophile and the proton donor are highlighted with magenta and green, respectively (based on Sakamoto and Toyohara, 2009, *Comp Biochem Physiol B* **152**∶ 390; Parsiegla *et al.*, 2008, *J Mol Biol* **375**∶ 499; van Santen *et al.,* 1999, J Biol Chem **274**∶ 30474).
(PDF)

Figure S2 **The maximum-likelihood phylogeny of GH45 family proteins.** Labels for the coleopteran species belonging to the superfamily Curculionoidea are olive-colored and all other coleopteran sequences colored in black belong to the superfamily Chrysomeloidea. *D. v. virgifera* sequences are shown in red. Their species name abbreviations are found in Table S5. Other sequences are colored as follows: mollusks (purple), *Cryptopygus antarcticus* (Collembola, black), *Hypsibius dujardini* (Tardigrada, black), protists (dark green), plant-parasitic nematodes (grey), fungi (cyan), and bacteria (brown). The scale bar represents the number of amino acid substitutions per site.
(PDF)

Figure S3 **Multiple alignments of** ***D. v. virgifera*** **GH16 family protein sequences.** A. GH16 family proteins identified from the D. v. virgifera transcriptome. B. The active site region sequences. GNBP sequences are boxed. The catalytic nucleophile and proton donor residues are highlighted with magenta and green, respectively (based on Viladot *et al.* 1998, *Biochemistry* **34**∶ 11332). C. The N-terminal conserved domain sequences of GNBPs. Residue shown to be within hydrogen-binding distances and involved in hydrophilic interaction with lamitrihexaoses from *Plodia interpunctella* and *Bombyx mori* proteins are highlighted with yellow and Arg’s involved in binding of triplex β-glucan are highlighted in light blue (based on Kanagawa *et al.*, 2011, *J Biol Chem* **286**∶ 19158).
(PDF)

Figure S4 **Multiple alignment of the potential GH5 family protein sequence identified from** ***D. v. virgifera*** **with four fungal GH5 proteins (A) and the maximum-likelihood phylogeny including other known GH5 family proteins (B).** The potential amino acid residues for the catalytic nucleophile and catalytic proton donor are highlighted with magenta and green in the alignment, respectively (based on Larsson et al. 2006, J Mol Biol 357∶ 1500). Coleopteran proteins included in the phylogeny are found in Table S5. The D. v. virgifera sequence is shown in red. The GH5 protein sequences are classified into subfamilies according to Aspeborg et al. (2012, *BMC Evol Biol* **12**∶ 186). Bacterial, plant, fungal, and nematode sequences are indicated by brown, green, cyan, and grey. The numbers at internal branches show the bootstrap support values (%) for the maximum-likelihood and neighbor-joining phylogenies in this order. Only bootstrap values higher than 70% are shown.
(PDF)

Figure S5 **GH1 family gene sequences identified from the** ***D. v. virgifera*** **transcriptome (A) and the maximum-likelihood phylogeny of GH1 family proteins (B).** In the alignment, the labels for partial sequences are shown in italics. Potential residues for the catalytic nucleophile and the proton are highlighted with magenta and green, respectively (based on Marana et al., 2001, *Biochim Biophys Acta* **1545**∶ 41; Scharf et al. 2010, *Insect Biochem Mol Biol* **40**∶ 611). In the phylogeny, labels for the coleopteran species belonging to the superfamily Curculionoidea, *D. v. virgifera*, and other beetle sequences are shown in olive, red, and orange, respectively. Their species abbreviations are found in Table S5. Arthropod, other metazoan, nematode, fungal, plant, and bacterial sequences are indicated by black, purple, grey, cyan, green, and brown, respectively. The numbers at internal branches show the bootstrap support values (%) for the maximumlikelihood and neighbor-joining phylogenies in this order. Supporting values are shown only when higher than 60%. The scale bar represents the number of amino acid substitutions per site.
(PDF)

Figure S6 **GH31 family gene sequences identified from the** ***D. v. virgifera*** **transcriptome (A) and the maximum-likelihood phylogeny of GH31 family proteins (B).** In the alignment, the labels for partial sequences are shown in italics. In the phylogeny, labels for the coleopteran species belonging to the superfamily Curculionoidea, *D. v. virgifera*, and other beetle sequences are shown in olive, red, and orange, respectively. Their species abbreviations are found in Table S5. Arthropod, other metazoan, nematode, fungal, plant, and bacterial sequences are indicated by black, purple, grey, cyan, green, and brown, respectively. The accession numbers shown in parenthesis are from NCBI except for: BGIBMGA012077-PA and BGIBMGA013995 from SilkDB (http://www.silkdb.org), DPOGS202361 from MonarchBase (http://monarchbase.umassmed.edu), and Px016165 from Diamondback moth Genome Database (http://59.79.254.1/DBM/). The numbers at internal branches show the bootstrap support values (%) for the maximum-likelihood and neighbor-joining phylogenies in this order. Supporting values are shown only when higher than 60%. The scale bar represents the number of amino acid substitutions per site.
(PDF)

Figure S7 **GH27 family gene sequences identified from the** ***D. v. virgifera*** **transcriptome (A) and the maximum-likelihood phylogeny including representative GH27 family proteins (B).** Labels for the coleopteran species belonging to the superfamily Curculionoidea, *D. v. virgifera*, and other beetle sequences are shown in olive, red, and orange, respectively. Their species abbreviations are found in Table S5. Arthropod, other metazoan, nematode, fungal, plant, and bacterial sequences are indicated by black, purple, grey, cyan, green, and brown, respectively. Bacterial sequences were used as outgroups. The numbers at internal branches show the bootstrap support values (%) for the maximum-likelihood and neighbor-joining phylogenies in this order. Supporting values are shown only when higher than 60%. The scale bar represents the number of amino acid substitutions per site.
(PDF)

Figure S8 **The distribution of E-values obtained from blastx similarity search against the UniProt protein database using the assemblies generated by three programs using the** ***D. v. virgifera*** **egg samples.** The numbers of contigs are 18,173 in Mira (blue), 11,035 in Trinity (red), and 9843 in Velvet/Oasis (green). E-values are shown as −log_10_ (E-value) except for E-value = 0. Note that there is no significant difference between Trinity and Mira (*t-*test *P*>0.5 for both Evalue ≤10^−100^ and for all E-values).
(PDF)

Table S1 **Summary statistics for** ***D. v. virgifera*** **transcriptome sequencing and assembly.**
(PDF)

Table S2 **Summary of** ***D*** **.** ***v*** **.** ***virgifera*** **transcriptome sequencing and assemblies.**
(PDF)

Table S3 **Summary statistics for hybrid and pooled-data assembly of** ***D. v. virgifera*** **transcriptome.**
(PDF)

Table S4 **Expression analysis of the** ***D*** **.** ***v*** **.** ***virgifera*** **GH family genes identified in this study.**
(PDF)

Table S5 **Coleopteran GH family gene sequences used in this study.**
(PDF)
