# Correction: Molecular Evolution of Glycoside Hydrolase Genes in the Western Corn Rootworm (*Diabrotica virgifera virgifera*)

**DOI:** 10.1371/journal.pone.0102818

**Published:** 2014-07-10

**Authors:** The PLOS ONE Staff

There is an error in the affiliation for co-authors Richard ffrench-constant and Arnubio Valencia. The correct affiliations for the co-authors are:

Richard ffrench-constant - Biosciences, University of Exeter, Penryn, United Kingdom^6^

Arnubio Valencia **-** Department of Entomology, University of Nebraska-Lincoln, Nebraska, United States of America^2^

Departamento de Producción Agropecuaria, Facultad de Ciencias Agropecuarias, Universidad de Caldas, Manizales, Colombia^7^
