# Supplementary material for: Molecular Evolution of Glycoside Hydrolase Genes in the Western Corn Rootworm (Diabrotica virgifera virgifera)
Source: PLoS One. 2014 Apr 9;9(4):e94052. doi: 10.1371/journal.pone.0094052 (PMC3981738; doi:10.1371/journal.pone.0094052)
Supplement: Figure S1 — GH family gene sequences identified from the D. v. virgifera transcriptome. Amino acid sequences of GH45 (A), GH48 (B), and GH28 (C) are shown in alignments. The labels for partial sequences are shown in italics. Potential residues for the catalytic nucleophile and the proton donor are highlighted with magenta and green, respectively (based on Sakamoto and Toyohara, 2009, Comp Biochem Physiol B 152∶ 390; Parsiegla et al., 2008, J Mol Biol 375∶ 499; van Santen et al., 1999, J Biol Chem 274∶ 30474). (PDF) [file pone.0094052.s001.pdf]

A

```
GH45-1   MLSL-----KIAVAILSLAG--VTIAQDLTPIPGGKSGDGVTTRYWDCCAPSCAWYPRIHTQNGVPIQTCKADGVTPSDKDLNA-QSGC-
GH45-2   MKLLVA---IAFLGYVAAGSFGRCPGPDIVPIPGGLSGDGITTTYWDCCAQTCAHRQNVKTDNGIPVQTCAIDGTTNITIDQNGIVSGC-
GH45-3   MKYLVV---ITFLGYVAAAS--SDRSPEIVPIPGGISGDGITTRYWDCCAPSCAYYGFIKTKNGIPDQTCQIDGVTNSTKDNNA-QSGC-
GH45-4   MYTGIVNIFLVSIAIVTASS--KESSPDIVAIPGGLRGDAITTRYWDCCVVSCSWDANVHTKNRQPVKSCQKNGATYSTRENNG-NSVCY
GH45-5   MKTFTV---FASLIVFGASL--KEPSPEIIPVPGGLSGDAVTTRYWDCCGVSCSWDGIVHTKNGIPVRSCEKDGKTYSTKENNA-QSTCW
GH45-6   -------------------------------------------RYWDCCKPTCSWPGNVNYKT--PVKSCQHDGVTAI--DPET-QSGC-
GH45-7   MKIAILV--SALVALAVATP--LEQSPEIKFIEKGISGEGTTTRYWDCCKPSCSWRGNVHTPSGVPVASCDRSGVNRV--DANA-KSGC-
GH45-8   M---IFN--CFIFSVVLAVT--LAYSPEIKKIVGGKSGYGTTTRYWDCCKPSCAWKENIKTPDMEPIATCATDGVTVV--NASV-QSGC-
GH45-9   MIFII----FSLLAFVGLAP--SIDALELTPVEGGLSGNGSTSRYWDCCKPACAWPSNV-PHSPRPVTSCKADGITPI--NPDA-MSGC-
GH45-10  MIPLPI---LLVLAVATSIK--AEVSPDIIAVPNGLSGKGITTRYWDCCKPSCAWADNVNTPDKQPLKSCRVDGEAVA--PPND-PSGC-
GH45-11  MKYTITS--LLLLAAYVAATSLNNQNIVIKKIPGGLSGVGTTTRYWDCCKATCSWPGNVEYKK--PVKACQADGENAN--DPEN-ESGC-

GH45-1   -EVGGVAYTCTNQSPKIINETLAYTFVAASFAGGLDY-ADCCICLVMDFKG-KLAGKRLLAQVTNTGEA---LGQNHFDIQMPGGGVGIY
GH45-2   -RVGGQAFACSNQQPYVVSDTLALGWSAASFTGGIDN-SKCCSCFLLSFKD-QLAGKQMLVQLVNSGTD---LASNHFDLQIPGGGVGIW
GH45-3   -EQGGVAYTCSNQQPSVINDTLAFGWAAASFQGGIDT-SKCCHCILLSFKD-QLAGKQMLVQIVNTGSD---LNENQFDLQIPGGGVGIF
GH45-4   PDHPGNAYVCNNNSPFVVNSTLAYGFAGVSFQGGADV-EHCCHCYLLSFKG-KLQGKQMLVQTINTGAD---AVAHHFDLQIPGGGVGYN
GH45-5   -NENGPAFTCSNQVPFVINSTLSYGFAAVSFVGSTDT-GHCCQCYLLKFQG-QLKDRELLVQAINTGSD---LTTNQFDLQIPGGGVGLY
GH45-6   --VGGGAYVCTNQAQRSVNDSIALGFVAAKFIHS-NR-NMCCSCIVFRFKPAELAGKQMVLQVTNTGDDDPHATHNEFDIAMPGSGVGYY
GH45-7   -EGGGSAYMCNSQQPWAVNSTLAYGFGAASFSNGVDV-SLCCACFLLSFKD-QISNKKMIVQVTNTGSD---LSHNHFDIALPGGGVGIF
GH45-8   --IGGTSYMCNNQQPFVVNETLGYGFAAVSFSGGVDN-DLCCSCYLLTFQN-QINNKKLVLQFTNTGGD---LGSNQFDIALPGGGVGAF
GH45-9   --ENGTAYTCTNQQPFIVNQTYGYGFAAAYLIGGPSTNNFCCACFLLNFTD-QIKYKHMVVQVTNSGTN---FDKNEFVIALPGSGVGDH
GH45-10  -DINGSSFVCNNNQPYVVNSTLSYGFASASFSGGIDT-SMCCSCMLLNFEG-QLKGKQFLVQLTNSGEE---YQTNQFDLGIPGGGVGLF
GH45-11  --IGGQSYICTKQSGFAINSTLAYGYVAARFHGT-TR-NMCCSCVLFSFQPQELANKKMLVQVTNTGNA-PETNTNLFDIAMPGSGVGYY

GH45-1   NLGCKTQWNAPDDGWGERYGGVTDIKGC-KQLPEQLQEGCRFRFTWMKGVPNPPVSFYQIKCPEYFVGVSKCGDL---
GH45-2   NHGCDAQWGAGENGWGRRYDGVSSLEEC-CLLPEVLQPGCRFRFQFMEGVYRPNVTFQEVQCPAELIAVTACGNLNY-
GH45-3   NLGCMTQWGTGEDGWGRRYGGVSSIEEC-SILPEVLQPGCRFRFQFMEGVDNPKVSFQEVKCPAELVAVSACGDLD--
GH45-4   TQGCRIQWNAPENGWGDRYGGVHSEQEC-NQLPWQLQAGCKFRFQFMQGVSNPDVSFQEVKCPSQLVSITGCGDL---
GH45-5   N-GCVKQWNAPVDGWGERYGRVTSVEGC-DQLPVQLQDGCKWRFEYLEGVSNPSATFYEVKCPSELIAITNCGDRD--
GH45-6   TQGCSSQWNADVSKWGDQYGGVHSIEEC-HNLPAHLQPGCEFRFTWMKGYSNPDIEFDEVVCPKRLTDISGCYPASHP
GH45-7   TQGCHDQWNAPWNGWGDQYGGVHNRGEC-ATLPQALQSGCYFRFDFYQNANNPRMHFDQVQCPAEIVARSGCSL----
GH45-8   NQGCHDQWNAPWTGWGQQYGGISSREECLSLLPKELQSGCLFRFDFMQNANNPQMYFEQVECPAELVKISGCSLPL--
GH45-9   PEGCHDQWNAPWTGWGDQYGGVHMRSECVTLLPEELQEGCKFRFDFMETAANPLVSFQQVVCPDELVKISGCRIPE--
GH45-10  PKGCTAQWNAPSTGWGDLYGGVHTEEEC-NELPEVLQPGCKWRFTFMEGVSNPEVTFYQVQCPRELVERSGCVL----
GH45-11  TQGCTSQWHTDVSSWGDQYGGVNSLQEC-YNLPQPLWEGCAFRFNWMLGYSNPDVSFEEVECPQELLSISGCDPISHP
```

B

```
GH48-1   ---MRLGLFVLFCVTSTALAGTYTDRFLTQYRKIHDSNNGYFSKEGIPYHSVETLIVEAPDHGHETTSEAYSYYVWLEAVYGKVTGDFSS
GH48-2   ------------------------------------------------------------------------------------------
GH48-3   MTPLHLLVLAVIIMNHASCESVYKQRFLEQYNKMHDPNNGYFSSKGIPYHAVETLVVESSDYGHETTSEAHSYYIWLEAMYGGITNNFSR

GH48-1   FNNAWNNLETYIIPVYSSQPTNSFYTPGHPATFIPEQDDPSQYP-SQIDSSVPVGQDPLHQELVNAYGSHEVYGMHWLLDVDNIYGFGNT
GH48-2   ------------------------------------------------------------------------------------------
GH48-3   FNEAWEIMEKYIIPVHESQPNTNLYNPSHPAGYGPEQEYPEDYPVGPVDPPAPVGIDPLYQELVDTYGTSDIYAMHWLTDVDNVYGFGNS

GH48-1   PGNCNLGPSAGGPSYINSYQRGSMESVWRTIPQPTCDNFRFGGNHGFLDLFTKDNSYAQQWKFTNAPDADARAIQAAYWAGQWAQQSGQL
GH48-2   ------------------------------------------------------------------------------------------
GH48-3   PGNCELGPNEPGPSFINTYQRGPRENAWKTIPQPTCDSHKYGGPEGFGPLFSTGD-HAPNWKYSVAPDADARAIAAAFWASRWATKSGHL

GH48-1   GTIQGTLAKAAKMGDYLRYALFDKYFKQVGNCDNRWSCPGGYGKSSAHYLLGWYYAWGGSVDTNGGWAWRIGDSAAHFGYQNPLAAYALA
GH48-2   ------------------------------------------------------------------------------------------
GH48-3   SEITDTLQKAGKLGDYLRYCFFDQNFKRIGNCIDPYKCPGGTGKDSAHYLLGWYFGWGGSISSEYGYSWRIGDGVAHFGYQNPMAAYALI

GH48-1   NDPNLRPKGATAVSDWQTSLERQLEFYEWLQSAEGAFAGGATNSINGHYDSPSSDLTANTFHGMYYDWEPVYHNPPSNRWYGMQSWSVDR
GH48-2   ----------------------------------GAFGGGATNTWNGRYDTPPQELTTNTFHGMFYDWEPVYHDPPSNRWYGMQSWSTDR
GH48-3   NEPNMTPKGATAVEDWQISLDRQLELYDYLQSVEGAFAGGVSNSWNGRYEQPPEELMDNTFHGMFYNWEPVAYDPPSNQWFGMQPWSTDR

GH48-1   LAQYYYVTGDSKAKSVLDKWVNWILKETTIEAGKSFKLPSQLSWSGNPPNVHCTINAYTTDVGSASGTARTLAYYAAKANHAQAKEVAKE
GH48-2   LAQYYYVTGDATAKTLLDKWVKWVISEIKFE-GTGYTHPDHLEWSGQPPNVHQVTSYSDDVGTASSTA----------------------
GH48-3   LAQYYYITGDDKAKKILDKWVSWIIANTYFE-GDDYRIPSTLDWVGVPPNVHCKVVYYGNGVGPAAATARTLSYYAARANHAEAKNLAKK

GH48-1   ILDIMWNNFQTSKGVSSPEVADTYTQFNEPVFVPNGWYGTYPKGDVIQSGATFLSLRSWYKSDPDWNKVQTYLNGGSAPTFTYHRFWAQA
GH48-2   ------------------------------------------------------------------------------------------
GH48-3   ILDSLWNLHRTPLGIAVEEQPEIH--FNQSVYVPKDFHGVYPNGDVIDSDSTFISMRSFYKNDPQWNKIESYMNGGPAPKFTYHRFWDQT

GH48-1   DIAISNGVYGILFNE
GH48-2   ---------------
GH48-3   DVALGFGVYGLLFDE
```

C

```
GH28_1  M--TNLTLLIVFSVIVATIAIPFNST-KNIGDGCTISNIWEVENVVKNCKNIVVNNLYVPGGQKLELKLHSGTVLKFQGTTTFQHSNW-E
GH28_2  M--ATLTLFLVLCAAVATSAISLNST--NVGAGCTISKIGEVDNVVKNCKNIVINNLSVPGGKTLKLDLHPGTTLKFQGTTTFQHTNW-E
GH28_3  MYYTIMCYLFLFLLFNAALVICKCSP-----TNCEITNFDQVSDTVHRCSDIIIRNLDVPAGQTLELDLKQGASLTFEGITTFDYTNW-S
GH28_4  M-NLFIIENFIVVVLLNSLLFISCVD-----QPCTITNFSQVSEVLQSCKNITISNLNVPAGQQLYLELLNDSSVTFEGVITFGVAQW-K
GH28_5  M--------SYTKFLIVAFISTVSAN-----NNCTITEFAQVAEIVKECSNIVINDLVVPAYSTLLLNLKNGSRVTFTGNVLFEVGYW-E
GH28_6  M--RTIQLFEYFFLCSIAYASNLT-------ASCTISRFDHVDTVVSQCKSITVESFAVPAGQTLKLHLQYGTTLTFNGNIAFGYSEW-D
GH28_7  MIKTGMSLVLFFLGVVLAQE-----------YDCEINSIDQVLPVIEKCSVITVKNLWVPSGQTLELSLKDNSHLIFDGNVTVGVKYQDE
GH28_8  -------------------------------------------------------------------------------------------
GH28_9  MSSNKLIYSLLFVVISAAAKSLNE-------DCCTITEYSQVPDVVETCKNIVISNLRVPANKTLNLNLQDGSELTFEGRTYFDYFEW-K
GH28_10 ------------------------------------------LNDC-----------------------------------------
GH28_11 M-------LFIYKILVLLIVVSIAAS-----DICTISNYDLVDEALSSCIDIVISNLTVPSGKTLNLNLKERSTVTFDGVITFEVSFR-T
GH28_12 M-----CYFNKFSLLLLLYSPLLSKS-----DPCTVTQFSQVAQAVNDCTNLIISNLVVPGGQTLELHLKYGATVTFEGTTVFEVAHW-E
GH28_13 MGFSVLLFLSLLALISGTSVLQATNNTEAVGDSCTITQYSQVDGVLKSCTNIILSNVEVPSGKSLNLYLRDGSTLTVRGTISFDVGYN-N
GH28_14 -------------------------------------------------------------------------------------------

GH28_1  GPLVEITGSNLHVSGA-GAILDGLGAQYWDGY-GDKGAVKPKFLKIRTT-GSTFDNIHLLNCPRQCVSILSSKQTTLTNFNIDVSAGDIT
GH28_2  GPLISISGSNLHVSGS-GAVLDGLGSKYWDGK-GDKGAKKPKFFKIRETTGSTFDSIHLLNCPHQCVSIQNSKKTTLNNWNIDVAAGDIN
GH28_3  GPLIRINGSGFTIKGAPGSLLNGQGDLYWDHL-GDKGPKKPQFIKIEAFDGSIIENINLLNCPHHCVYVGKSDGLTIRGWVIDNSYGDQN
GH28_4  GHLIVVKGHNVIIQGAPGSILNGQGQKYWDGQGGGGGTTKPKFFYIETTGGSIFKNIYLYQCANWCVGIG-SKDVIITGWTIDNTAGDKD
GH28_5  GPLLEISGDGVEVQGNAGHIINAQGEKYWDGQGGSGGVTKPRFVVISTTGGSVLRNIYLLNCVYFCVGIH-ASDLTLSGWTIDAVAGNTR
GH28_6  GPLMWIKGDGITIQGTESHLLNGRGELWWDGHGDHSNKKKPQFMLIQATGNSLLKDIKVKNCPHTCIGISDSHDITLQHWTIDCQDGDTK
GH28_7  VPLIRISGANLFIEGRKDAVINGQGEKYWDGKGIEGKNRKPVLLEISAQ-ESLLKNINIRNCPQKCVNILKSANSSFTGWNIDITDGFKD
GH28_8  GPLVRFRGSQIVVQGAKGSFLDGQGALYWDGMGGNGGVTKPYFFQIETTGGSIFRNIHLLNCPHHCVIIS-STDLTITGWNIDVSAGDKG
GH28_9  GPLVNITGDDLIVRGAPGHVLDGQGELYWDHL-GGKGIKKPKFIRLQGN-NSRYENIYLKNCPVHCASVAVS-NSIIDGWLIDVSEGDKN
GH28_10 ------------------SILDAQGEKYWDGQGGAGGVTKPKFFYVQTTGGSILKNIYLLNCAHFCVGVG-AKDTTITGWTIDSVAGNKD
GH28_11 GFLVSVAGKNVLVQGAPGSILNGQGEKYWDGF-GDNGVVKPKFFRVATSGGSIFRNIYLLNCPHFCVGVY-ATDVTLTGWTIDVLAGNTR
GH28_12 GPRIEKKEENVEVQGASRSILNAQGEKYWDGHGGSGGVTKPRFVQISTTGGSVFKNIHLKNCALFCVGIR-ASDLTISGWNIDSHEGRKK
GH28_13 IWLVTISGNNIKVIGEKGSLFHGHGEKYWDGHGGSGGVTKPKLLQILNVNNAHFSNINLKNCPMFCTGITKAKDLTIDGWNADCAEGDKL
GH28_14 ----------------------------------DKGNKKPKFFKIQATGGSVFKNINLLNCPHQCVSIQNSKQLTISNWNIDVSAGDKN

GH28_1  HL-ATNTDGFDLSD-SDGITIENSVVRNQDDCVAVNSGKNYHFNKLNCNGGHGLSLSVGMSKNDSP--------RNHVEDVTFSNCIVSN
GH28_2  SL-GHNTDGFDLCE-NEEITIQNSIVHNQDDCVAVNSGKHYHFNKLTCVGGHGLSLSVGTSTTDPS--------KNYAEDINFSDCSVSN
GH28_3  NFTGHNTDGFDVSA-ASNLIIEDSTVINQDDCIAIRHGYNILVRNMYCAGGHGLSLSAGFSYTTFQ--------ENTITNVVIKDSVIAR
GH28_4  MI-ALNTDGFSLID-SENVLIENSTIMNQDDCIVVRRGNNMTFRNIKCFGSHGLSFATGFHETDGPFGHKEDDAEDIATDITFEDCLVAN
GH28_5  G--GLNTDGFGIGN-GQNILIENSVIMNQDDCVVVNSGSDMVFRNLECYGSHGLSFSIGDSHNDDA-------AANTIKNITFSDCLVAN
GH28_6  G--GANTDGFDIAK-SYKVTIKDTTVRNQDDCICVNQGQHLVFQNMHCIGGHGLSLASGL-WDTYE--------LNTIYNVTFQNSIVEN
GH28_7  NV-GVDTHGFAVAN-SSDIIIKESNIINQGDCIVVNQGSDLHFEQIVCRGSQGITVRPEWEY------------ENYIRDVIFDDCTVIE
GH28_8  NL-GHNTDGFDVIY-GENIVIENSIVQNQDDCVAINRGKNMLISNLRCYGGHGISLSVGFSHRSYK--------HNTVHNVTFIDCVVAR
GH28_9  NFTGHNTDGFDLS--STNLILQNSIVKNQDDCVVVNVGANILVRNMACYGGHGLSISAGFSKDDFA--------KNSVYNVIFEDSLVHR
GH28_10 LI-ALNTDGFGVSSHSDNILIENSVIMNQDDCVVVNQGTNMVFRNLHCYGSHGLSFAVGFGGRDKP------EDDSVASNITFENCWVAN
GH28_11 G--GLNTDGFGIHS-GRNIVVQDSVVMNQDDCVVVNSGTDMIFRNLQCYGSHGLSFSVGSKTEENA-------EAGIVQNITFLDSLVAN
GH28_12 ---GKNTDGFGIAA-GNNIHIENSSVDNQDDGIVVNGGTNMVFNGIKCTGSHGLSFSAGSNTNDHA-------KYATINNITFSNCELKD
GH28_13 ---GRNTDGIGISW-SQHVYINNAYIHNQDQCLYVNQGSDMVFTGIHCVGSNGICATAGFSKTSYE--------ENTTKNITFHNCVLEG
GH28_14 KL-GHNTDGFDISG-SDGVNFEYCTVQNQDDCVAVNSGKNLHFNHMTCSGGHGLSLSIGMSKTDSS--------KN--------------

GH28_1  SLNGIHIKT-HSDAGKGYINGVEYRNIILKDITNYGINVQQDYQGGHSTGYPTSNIPINGLKLEGVTGSLRS-----GQPVYIFCGN-NA
GH28_2  SRNGIHIKT-HTDGANGYIRGVTYKNIKLSGITHYGINVQQDYNGGGSSGYATSNIQINGLHLQSVTGSLKS-----GKAVYILCGN-KA
GH28_3  SANGIHVKT-HADAYNGRIQNVTYENIFMSGLINYGINVQQDYVNGSATGVANNNIPIYNLNLINIRGTVRDSDSEKSMPVYINCGK-SA
GH28_4  GLYGIHIKT-APNGKRGRIENVLFKNIKLSGIQEDGIYIQQDYGD---IGKQDSNVTIKNLTLKNVYGSLQGIL---TRPIHIFCGNQGT
GH28_5  GLYGIHVKT-KK--GTGVLTDVTYENIRLSGITEDGIYINQDYGD---IGNYSREIEITNLKMSNIYGSVQGVL---TRPVHIVCSN-DK
GH28_6  SRNAIHIKTIPVNNKKGEITSITYDNIKLIGISYYAINVQEDYTNDGPTGHPLGNIPVKDLKIHNVYGTMTGSN---SVKAYILCGS-GG
GH28_7  GQTGIRVVT-SPHQPEGYISNVIYRKIHLTGILFRGIDIRQDLDD---EGRPSGNVKITELDISDVKGNMTDKY---VRSVYIWCGP-DG
GH28_8  SENGIHVKT-HNDGYLGEIKNVTYKNIEFVDILNYGVNVQQDYANGTSTGNPTNNIPITNLSLINVHGTVKGSH---ATGVYILCGS-AG
GH28_9  SPNGIHVKT-HADSGPGIIQNIIYRNIRFEDINNFALNIQQDYVNGEATGIPGTNIPIVGLSLDNISGWMKSFNESPTLEALILCGD-GA
GH28_10 GLYGIHVKT-GAVGNKGRIENVVFRNIKLSGIQEDGIYIQQDYGN---IGNLESEVKIHNLTVENVTGSVQGVL---TRPIHIFCGNRST
GH28_11 GLYGIHIKT-KK--GSGTIRDVIYENIQLSGITEDGIYINQDYED---IGNYSREFEIHNLKISNVYGSIQGLL---TRPVHVVCNE-NK
GH28_12 GAIGIHVKT-KR--GTGLITNVTYDHITMTGMQKDGIYINQDYGD---VGNTTRDFQITNLKVSNVEGSIHGKG---ARAVHIVCND-KK
GH28_13 GLTGVQVIA-MADGGPGEITDIHFQSIILKGVRQQGVYVQMDYGN---DGHPNNNIAVTGLKLSHVTGTVSGNS---ARPYYIKCG--AK
GH28_14 -------------------------------------------------------------------------------------------

GH28_1  CFNFNWSGVSITGGNQQSSCNYHPNGYYC-
GH28_2  CSNFNWSGISIYGGNEKNGCNYHPNGFSC-
GH28_3  CHEWSWSNINIAGGSNSSICNYTPDGYQC-
GH28_4  CSEWIFSNINILGGN-RSYCNYQ-------
GH28_5  CQNWTWSNINILGGG-KNYCNFQPTEFIC-
GH28_6  CTNWNWSEINVSGAAKPNSCNFTPNGFSC-
GH28_7  CANWNWSDIDIENAEVENACNFLPNNWSCW
GH28_8  CIDWNWSEISITGAKRENSCNYVPSGYHC-
GH28_9  CDKWEFHNIDITGAQNNSICTFQPEGYSC-
GH28_10 CDDWKFSGINILGGG-QSYCNYVPDEFHC-
GH28_11 CSNWTWSNINILGTG-KSYCNYIPDGFRC-
GH28_12 CANWQWSNIDISGGA-KDYCNFHPTGFDC-
GH28_13 CSNWIFNDVQVTGGGVKSSCNYKPSGFNC-
GH28_14 ------------------------------
```

Figure S1. GH family gene sequences identified from the *D*. *v*. *virgifera* transcriptome. Amino acid sequences of GH45 (A), GH48 (B), and GH28 (C) are shown in alignments. The labels for partial sequences are shown in italics. Potential residues for the catalytic nucleophile and the proton donor are highlighted with magenta and green, respectively (based on Sakamoto and Toyohara, 2009, *Comp Biochem Physiol B* **152**: 390; Parsiegla *et al.*, 2008, *J Mol Biol* **375**: 499; van Santen *et al.* 1999, *J Biol Chem* **274**: 30474).
