# Supplementary material for: Molecular Evolution of Glycoside Hydrolase Genes in the Western Corn Rootworm (Diabrotica virgifera virgifera)
Source: PLoS One. 2014 Apr 9;9(4):e94052. doi: 10.1371/journal.pone.0094052 (PMC3981738; doi:10.1371/journal.pone.0094052)
Supplement: Figure S3 — Multiple alignments of D. v. virgifera GH16 family protein sequences. A. GH16 family proteins identified from the D. v. virgifera transcriptome. B. The active site region sequences. GNBP sequences are boxed. The catalytic nucleophile and proton donor residues are highlighted with magenta and green, respectively (based on Viladot et al. 1998, Biochemistry 34∶ 11332). C. The N-terminal conserved domain sequences of GNBPs. Residue shown to be within hydrogen-binding distances and involved in hydrophilic interaction with lamitrihexaoses from Plodia interpunctella and Bombyx mori proteins are highlighted with yellow and Arg’s involved in binding of triplex β-glucan are highlighted in light blue (based on Kanagawa et al., 2011, J Biol Chem 286∶ 19158). (PDF) [file pone.0094052.s003.pdf]

A

```
GH16-1  M--ILILFAFSAILFNGVHS---VCLKNSITTASGLHAPKNKSLCSGQLIFEDNFNFLDTSK-WHHEQTLSGG-GNNEFEWYTDDRRNSYTSNGQLHIKP
GH16-2  M----NLMKFMALIFHNLRG-----CEKSVTTVSGTHAPL--LPCKGDLIFKDEFNNLDLKK-WQHEVSLSGN-GNWEFQWYTNNRSNSYVENGVLHIRP
GH16-3  M---IVICLIVALLVQNIHG-----CGIPLTTVSGSHAPK--AVCSGDLIFKDDFNTLDMKT-WQHENTLGGG-GNWEFQWYTNNRSNSYVENGVLHIRP
GH16-4  MDRRVLVVLTVIVLLQNVHT-----CKKSITTVSGNYAPK--EVCSGKIIFEDNFDELDLNK-WNHEQTLGGG-GNWEFQWYTNDRNNSYVKDGELHIRP
GH16-5  MFKLIVITVLISIFYPTVYSQSRICQKPSITTASGTAAPTG-TICPGDLIFEENFNGPPFTKRWEHERTLGGGASNNQFQLYHNNRSNSFVRDGNLHICP
GH16-6  MFKLILHTILISIFYPTVYCQLGTCQRPSVTTASGIAAPTG-TIFPGDLIFDENFDGPTFTKWWTHESSLSGGANNNQFQWYQNNRSNSFVRDGNLHIRP
GH16-7  ----------------------------------------------------------------------------------------------------
GH16-8  --------------------------------------------------------------------------GNNEFEWYTNSRNNSYTSNGILHIKP
GH16-9  --------------------------------------------------------------------TLTGG-GNFEFEWYTDDKRNSYAENGKLHIKP

GH16-1  TFVADEY-GEKFLYSGTIDL-----GAKCTGSDNEGCRRTGSANAIVNPIKSARMRTLDSFSFKYGRVEVSAKVPSGDWLWPAIWMLPSKWV-YGSWPAS
GH16-2  TLVADDY-GNEFLYSGTIDVNGGSPADECTNPQFYGCSRTGSATNIVNPIKSARIRSLNSFSFKYGKVEVRAKVPAGDWLWPAIWLLPRYNQ-YSTWPAS
GH16-3  TLVADDY-GNEFLYSGTIDVNGGSPADECTNPQFYGCSRTGSATNIVNPIKSARIRSLNSFSFKYGKVEVRAKVPAGDWLWPAIWLLPRYNQ-YSTWPAS
GH16-4  TLLADQY-GNDFLYSGTIDL-----GKECTNSEWFGCRRVGTKTQILNPIKSARLRTANSFSFKYGKVEIRAKIPAGDWLWPAIWLLPTYWK-YGGWPTS
GH16-5  TLLADDN-GEAFLYNGTISLNGPYPIEHCTISGYDGCLRTGNDSIILNPIKSARIHTAFSFSFKYGRVEARVKVPLGDWLWPALWMKPRNNE-YSSWPSS
GH16-6  TLLANDT-GEAFLYNGTIDLNGAFPIGYCSIPVYDGCLRTGNNSIILNPIKSARIHSAYSFSFKYGRVVARVKVPLGDWLWPALWMEPRDNE-YSSWPSS
GH16-7  ----------------------------------------------------------------------------------------------------
GH16-8  TFVADEL-GEQFLQSATVDL-----GNKCTNPHNSGCKRTGSPGEILNPVKSARLRTLDTFSFIYGKVEVRAKVPTGDWLWPAIWLLPNKWIENGDWISS
GH16-9  TFVADEHGGDGFLYSGTIDL-----GKKCTNNDNEGCKRTGSANAILNPVKSARIRTLNSFSFKYGKVEISAKNPSGDWLWPAIWLLPSKWV-YGGWPRS

GH16-1  GEIDIMESRGNRQYY-DPSGQNVGTQLEASTLHWGPNPKFNKFMKTHWEKKNWNGGWDNAYHRYQVEWTPEHIKFSCDDQELGTITPPSGGFWQYGDLGP
GH16-2  GEIDIMESRGNRQLT-NPSGVNIGVQQIGSTLHWGPNPNYNQYARTHFE-ENLETGYDKAFHNYQVEWTPDYIKFAIDDEEIGRVTPPGGGFWELGELSS
GH16-3  GEIDIMESRGNRQLT-NPSGVNIGVQQIGSTLHWGPNPNYNQYARTHFE-ENLETGYDKAFHNYQVEWTPDYIKFAIDDEEIGRVTPPGGGFWELGELSS
GH16-4  GEIDMMESRGNRQLI-SQSGRKIGVQTIGYTLNWGPNPDNNLFYKTHFD-STLDVGYNQEFHKYQLEWTPDYIKFSIDDKEVGTIVPPSGGFWQLADLTS
GH16-5  GEIDILESMGNKGYI-K-NGVNIGVQEVGSTLHWGPYSALNRYPLTHYT-LNNADGWNADFHIYEMIWNTTSITFLIDHVVLGSVSPPDGGFWELGNF-S
GH16-6  GEIDILETRGNKNYI-K-NGLNIGVQQVGSTLHWGPY---------------------------------------------------------------
GH16-7  -----------KELI-KPNGQNIGTPLLNTNLHWGANAQTNRYAQTHYE-TTIPEGFDQAYHKYQLEWTPDYIKFSLDDREIGKVTPPSGGFWELGNLEK
GH16-8  GEIDIMETRGNKELI-KPNGQNIGTPLLSTTLHWGANAQTNQYAQTHYE-TTIPEGFDQAYHKYQVEWTPDFIKFSLDDREIGRVTPPSGGFWELGNFEH
GH16-9  GEIDVMESRGNRELIAKGSGQNIGADLVASTLHWGPDAKNNKFMKTHWE-KTVTGGYDKAYHKYQVEWTPDHLKFSIDDQEIGTVTPPNGGFWEFGDLQS

GH16-1  SGLPNPWSRGSRMAPFDQEFHLLINLAVGG-AYFNDEFNNKAYKKPWTSTEPYRQGMTKFWQANQKWKPTWNLNSDDSHLKVDYVRVWAL
GH16-2  SGLDNPWKRNSKMAPFDQEFYIVINLAVGGVNYFPDNANNSPRGKPWSNTSPN--AATNFWEGREQWLPTWNIGTEDSHLQVDYVKVWAI
GH16-3  SGLDNPWKRNSKMAPFDQEFYIVINLAVGGVNYFPDNANNSPRGKPWSNTSPN--AATNFWEGREQWLPTWNIGTEDSHLQVDYVKVWAI
GH16-4  TGLQNPWKNGTKMAPFDQEFHLLINVAVGGLSYFPDNAVNTPGKKPWTNTSPY--APATFWNARNEWLPTWKMETDESHLKIDYVKVWAL
GH16-5  TNTANPWAAGTKMAPFDQEFYFIINLAVGSTSWFSDYGVNDGYPKPWKNSDG-RASMTKFWKAKDLWLPTWNMTTDDSHFQIDYIRVYAL
GH16-6  ------------------------------------------------------------------------------------------
GH16-7  TRLPNPWRGSSKMAPFDAEFYLILNLAVGG-YFFPDQADNRSGKKPWSRTSPYRIGMTDFWRN---------------------------
GH16-8  TGLPNPWRGKSKMAPFDAEFHLILNLAVGG-NFFPDEADNRSGRKPWSHNVPYRIGMTDFWRNRQQWIPTWNLGTDDSHLKVDYVRVWAI
GH16-9  SGQQNLWKDGSKMAPFDAEFHLLINLAVGG-NFFPDDVDNKSGKKPWTSTEPYRQGM---------------------------------
```

B

```
Dvir GNBP-1          KLPKGDWIYPEIYLTP-ANEK--YGL-KSQSGQIRIAFTPGNSDL-----------------NHVLHGGLTIGRSVA
Dvir GNBP-2          KLPKGDWLYPILTINP-VKDE--YGP-GYDSGQITIAFCPGNAVL-----------------SHNVYGGIVISGSPV
Dvir GNBP-3          KLPAGDWLYPILYLNP-SAET--YGK-YYASGQMRIAYIEGNTG-----------------ANHILKGGVILGDSEP
Dpon AEE61901.1      KLPKGDWLYPELYLNS-ESEE--YGS-GYESGQIRIAFAAGNEGE-----------------SRKLEGGVILGSIPA
Dpon ENN74344.1      KLPKGDYVYPEIYLQD-VANP---------ESLILIAFTRGNENY---------EKNGIDIGGKQLYGGPYNISIKD
Dpon ENN74953.1      KLPKGDWLYPELYLNS-ESEE--YGS-GYESGQIRIAFAAGNEGE-----------------NRKLEGGVILGSIPA
Tcas XP_972063.1    KLPKGDWIYPQLFLNP-VSEE--YGS-DYASGQIRVAFLPGNQAM-----------------AQQLYGGCVLGPTTA
Tcas NP_001164284.1 KLPAGDWIYPQIYLEK-VSDP---------KDKIWISYARGNNQLLLK--------NKQDLGGNLLFGGLVIDPEEP
Tmol Q76DI2.1       KLPKGDWIYPELYLNP-VNEE--YGP-GYASGQIRIAFSGGNEDL-----------------CRDLRGGCILGSRPA
Slit ACI32819.1     KLPYGSWLVPEINLEP-LEHT--YGL-NYESGLMRVAFVRGNSYF-----------------AKKLYGGPVLGHSEP
Pxyl AFK24449.1     RMPRGDWLIPDILLEP-KENL--YGVRNYASGLLSIASVRGNTAY-----------------SKTLKGGPILCDKEP
Amel NP_001157186.1 KLPRGDWLFPLITLES-TDMC--TKN-SSIYCDIIIVHSNGNSVLMSQ--------DGNDLSGHFLLGGAHATDINS
Dmel GNBP1          KLPKGDWIVPLLLLEP-LTEW--YGQSGYESGQLRVALARGNSVLRMP--------RGKLVDGRSLYGGPVLSTDAH
Dmel GNBP2          KLPKGDWLFPYLMLQP--VST--YAE-THYAKQLRIAYARGNANLRTK--------QGDDISGNHLYGGGVVWHHGN
Dmel GNBP3          KMPRAQWVTPQIWLQP-RRPI--YGVDDYRSGQLRIAYTRPNGG------------------NLDLYGAAVLFADEP
Spur XP_001176934.2 KLPTGDWLWPAIWLLP-KHNG--YGE-WPASGEIDLVESRGNAD-IKD-ADG--LSAGVDQMGSTMHWGPFWPLNGY
Cint XP_002126690.1 KMPTGDWIWPAMWLLP-KTNS--YGS-WPASGEIDICESRGNTD-LKD-DQG--VSHGNDAMGSTLHWGPYWPVNAY
Cgig BAG82629.1     KMPKGDWIWPAIWLLP-HRNA--YGG-WPASGEIDVVESRGNTD-YHD-ENG--RSQGVDSFGSTLHFGPVYGYDPY
Dvir GH16-1         KVPSGDWLWPAIWMLP-SKWV--YGS-WPASGEIDIMESRGNRQ-YYD-PSG--QNVGTQLEASTLHWGPNPKFNKF
Dvir GH16-2         KVPAGDWLWPAIWLLP-RYNQ--YST-WPASGEIDIMESRGNRQ-LTN-PSG--VNIGVQQIGSTLHWGPNPNYNQY
Dvir GH16-3         KVPAGDWLWPAIWLLP-RYNQ--YST-WPASGEIDIMESRGNRQ-LTN-PSG--VNIGVQQIGSTLHWGPNPNYNQY
Dvir GH16-4         KIPAGDWLWPAIWLLP-TYWK--YGG-WPTSGEIDMMESRGNRQ-LIS-QSG--RKIGVQTIGYTLNWGPNPDNNLF
Dvir GH16-5         KVPLGDWLWPALWMKP-RNNE--YSS-WPSSGEIDILESMGNKG-YIK--NG--VNIGVQEVGSTLHWGPYSALNRY
Dvir GH16-6         KVPLGDWLWPALWMEP-RDNE--YSS-WPSSGEIDILETRGNKN-YIK--NG--LNIGVQQVGSTLHWGPY------
Dvir GH16-8         KVPTGDWLWPAIWLLPNKWIE--NGD-WISSGEIDIMETRGNKE-LIK-PNG--QNIGTPLLSTTLHWGANAQTNQY
Dvir GH16-9         KNPSGDWLWPAIWLLP-SKWV--YGG-WPRSGEIDVMESRGNRE-LIAKGSG--QNIGADLVASTLHWGPDAKNNKF
Dpon ENN72429.1      KVPLGDWLFPALWLMP-KDSK--YGS-WPLSGEIDVMESRGNRN-LTD-SSG--NIVGVQSVSNTLHWGPSKALDTW
Dpon ENN78830.1      KVPAGDWLWPAIWLLP-SDYR--YGG-WPVAGEVDLVESRGNRN-LTD-STG--LNIGTQLAFSTLEWGPSLEQNQY
```

```
Dpon ENN78831.1      KLPGGDWLWPAIWLLP-TDWK--YGS-WPVSGEIDLVESRGNRE-LTD-GSG--FNFGTQLAFSTLHWGPSPSENQY
Dpon ENN83076.1      KMPAGDWLWPAIWLLP-RYNA--YGT-WPSSGEIDLLESRGNKD-YVN-PNG--MNVGTQQVGHTLHWAPSVGANQW
Dpon ENN83093.1      KMPSGDWLWPAIWMLP-RFNS--YGR-WPASGEIDIVESRGNKN-LFN-PQS--VNIGTQEAASTLHWGPFNQVNQW
Tmol ACS36221.1      KLPTGDWLWPAIWMLP-RWNQ--YSG-WPISGEIDIMESRGNAD-LVN-ASG--ANIGSKLVSSTLHWGPAWNINMY
Tcas XP_970010.1     KLPAGDWLWPAIWFLP-RWNL--YSG-WPVSGEIDLMESRGNRN-LVN-GSG--VNIGTQMVGSTLHWGPASNYNMY
Aaeg XP_001652521.1 KLPQGDWLWPALWLLP-KGDT--YGY-WPKSGEVDLMESRGNRN-LVQ--NN--EKIGIQKVSSCLHFGDNPNVRSS
Aaeg XP_001664288.1 KLPAGDWMWPAVTFLP-KYNQ--YGG-WPASGEIDLLESKGNRD-LIK--NG--INVGIEQVTSKLQFGPNEGYNAG
Aalb AAV90645.1      KLPRGDWLWPALWLLP-KGDT--YGS-WPKSGEIDLLESRGNRN-LML--NN--GNIGVGKVSSCLHFGENPNLRSS
Cqui XP_001845811.1 KLPAGDWLWPAMWMFP-QQDS--YGV-WPKSGEIDLVESRGNRE-LYQ--DG--KHIGVKWLSSCLHFGESWNQKSE
Cqui XP_001845967.1 KLPTGDWMWPALWLMP-RTNQ--YGT-WPASGEIDLMEARCNVD-YRD-EEG--THLGVEQVLSTLHFGPNAWTNAY
Adar EFR28384.1      KIPTGEWLWPALWLMP-KLNQ--YGT-WPASGEIDLMESRGNLD-YTA--GG--QQIGVEHVGTTMHFGPYPALNGY
Acar ACI32832.1      KMPAGDWLWPAIWMMP-AFNS--YGT-WPASGEIDIVESRGNRN-MLS--NG--VNIGTQEAGSTLHYGPFPGLNGW
Sfru ABR28478.2      KMPAGDWIWPAIWLMP-AYNT--YGT-WPASGEIDLVESRGNRN-MFH--NG--VHIGTQEAGSTLHYGPYPAMNGW
Onub ACI32836.1      KMPAGDWLWPAIWLMP-AFNV--YGT-WPASGEIDLVESRGNRN-MLF--NG--VHIGTQETGSTLHYGPYPDLNGW
Bmor NP_001159614.1 KMPSGDWLWPAIWLMP-AYNK--YGT-WPASGEIDLVESRGNKN-MFL--NG--LHIGTQEAGSTLHYGPFPGLSGW
Mdar AAZ08492.1      KMPAGDWLWPAIWLLP-RYNE--YGP-WPASGEIDLSESRGNRN-LTI--DG--LNIGTEICSSTLHFGPFSPFDGW
Mdar AAZ08505.1      KLPAGDWLWPAIWLLP-RFNY--YGS-WPSSGEIDLTESRGNRN-LIK--NG--INIGSELTSSTLHFGPFWPLNGY
Cpun AFR46664.1      KLPAGDWTWPAIWLLP-RYNQ--YGS-WPASGEIDLTEGRGNKN-LIN--NG--QNIGSELSSSTLHFGPFWPLNGY
Ncor AEK64800.1      KMPAGDWLWPAIWLLP-RYNE--YGP-WPASGEIDLSEGRGNAN-LTS--KG--LNIGKELSLSTLHFGPYWPLNGY
Rvir AEK64797.1      KLPAGDWLWPAIWLLP-RYNE--YGP-WPASGEIDLSEGRGNTN-LTL--NG--SSIGTELSSSTLHFGPYWPVNGF
Rvir ADJ19024.1      KLPSGDWIWPAIWLLP-RFNF--YGS-WPASGEIDRTESRGNKQ-LIK--NG--QNIGSELTSSTLHFGPFWPLNGY
Pame AFR46666.1      KLPAGDWLWPAIWLLP-RYNQ--YGG-WPASGEIDLSEGRGNLN-YIS-PSG--QNIGSELSSSTLHFGPFWPYNGY
Cfor AGA16572.1      KLPAGDWIWPAIWLLP-RFNF--YGS-WPSSGEIDLLESRGNRQ-LIK--NG--QNIGSELTSSTLHFGPFWPLNGY
Cpun AFR46663.1      KLPAGDWIWPAIWMLP-RYNE--YGS-WPASGEIDLSEGRGNRN-LTL--NG--LNIGSELSSSTLHFGPFWPLNGW
Lmig AFD54026.1      KLPAGDWLWPAIWLLP-RNNK--YGG-WPASGEIDLVESRGNLG-LTQ--GG--VNIGAERVASTLHFGPYYPVDAY
Bgla ABO40828.1      RISKGDWIWPAIWMLP-RDNV--YGG-WPRSGEIDIMESRGNE----------VGIGISQVSSTLHWGPSPNENRW
Calb AAZ04385.1      KIPKGDWLWPAIWMLS-RDRS--YGG-WPRSGEIDIMESRGNTK---AVLWG--QNSGVNYVASTLHWGPDFNNNRF
Hdis BAH84971.1      KIPKGDWIWPAIWLLP-PGWPWKYGA-WPASGEIDIMESRGNVH-LSE-ANG--ATQGVDRVLSTIHYGASPSQHRQ
Pame ABR28480.1      KLPRGDWIWPAIWMLP-KNWV--YGD-WPRSGEIDIMESKGNDN-YYD-SNG--VSQGDDRMGSTLHWGPDANHNNY
Dpul EFX72300.1      KMPRGDWIWPAIWMLP-TDSA--YGT-WPRSGEIDIVEIRGNAD-LTC--NDGQGKIGNSKMFSTLHWGPGFSQNMF
Dpul EFX72306.1      KMPRGDWIWPAIWMLP-TDSA--YGT-WPRSGEIDIVEIRGNAD-LTC--NDGQGKIGNSKMFSTLHWGPGFSQNMY
Dpul EFX65834.1      KMPRGDWLWPEISLMP-ANNV--YGE-WPRSGYIGLVSVRGNNA-FTC--NG--QSAGNNVMESSLEWGLNNDHIKS
Dpul EFX69035.1      KMPKGDWIWPAIWMLP-TDSV--YGE-WPRSGEIDIVEIKGNAD-FSC--NG--YPIGRQLAGCTLHWGPDPGQNRY
Hmag XP_002158311.2 KLPQGDWIWPAIWLMP-KYSV--YGD-WPASGEIDLMESRGNEN-LTV-SKKDLTQIGNKRVSQSLHWGPYFPQNGH
Aque XP_003388466.1 KLPIGDWLWPAIWMLP-RHNQ--YGV-WPSSGEIDIMESRGNAIGYSE--------GGYDSFGSTLHWGIDYMYNFF
Mcir EPB91375.1      RLPTGDWLWPAIWMLP-KYSA--YGS-WPASGEIDILESKGNPK-YAH--------GNTSTIMSTLHWGPNTYQNKY
Bbas EJP68643.1      RLPKGDWLWPAIWMMP-EGDV--YGA-WPRSGEIDIMESRGNGPEYSE--------GGRNWYYGTLHWGPTPRSDAY
Cmil EGX89530.1      RLPKGDWLWPAIWMMP-ESDA--YGA-WPRSGEIDIMESRGNGPDYAE--------GGRNWYYGTLHWGPTPKTDTY
Clus XP_002618376.1 KLPKGDWLWPAIWLLP-KNNT--YGP-WPASGEIDIAESRGNGVHYPM--------GGHDVISSSLHWGPDVAQDQY
Fmed EJD07749.1      KMPRGDWLWPAIWMLP-KDEK--YGA-WPRSGEIDIVEARGNSASYPR--------QGNNFVRGSLNWGPLDTVV--
Gtra EPQ53062.1      KNPVGDWLWPAVWMLP-VNNT--YGP-WPMSGEIDIMESRGNGLSYTA--------QGSNYVHGSLNWGPLTWLNEV
Cant ACD93221.1      RLPKGKHLWPAFWMMP-QNSE--YGG-WPRSGEIDITEYRGQRP---------------QQILGTLHFGAAPDNKGD
Amac YP_008173608.1 KMPEGQGSWPAFWMMP-TDEV--YGG-WPRSGEIDIVEAVNLKAADAE------G-NPESHIYGTLHYGQEWPNNDS
Pfla WP_010607480.1 KLPSGQGSWPAFWMLP-TDEV--YGG-WPKSGEIDIMEAVNLKVASED------G-SVESNVHGTLHYGRDWPNNVH
Gjoo WP_017446697.1 KLPSGQGSWPAFWMLP-TDNV--YGG-WPHSGEIDILEAVNLKVPLEG------SDDLESSVHGTLHYGKSWPNNAS
Mmar YP_755478.1     QLPEGQGSWPAIWMLP-TDEV--YGG-WAASGEIDILEAVNLGEPCRE-----CRGDVENRVFGTLHYGGEWPENTY
Rmai WP_008695923.1 KLPVGAGIWSAIWMLP-TNDA--YGG-WAASGEIDIMEMKG---------------QEPDEVLGTLHYGDVWPRNAY
```

C

```
Dvir GNBP-1         YEVPDAVVEVYEPKGFTVSIPDEEGIK-LFAFHGKINEDF-DG-LEAGT-----------------FARDILKPRNGSWT
Dvir GNBP-2         YEVPAATVEVYHPKGFSVSIPDEDGIK-LFSFHGNINQEM-DG-REAGT-----------------FSRDILRPVNGMWT
Dvir GNBP-3         YNGQRTNGQKFNGNPVTFFGPDREQAQGLFNQFKNSVSEY-QQ-KNGGNGEIKVLNPSNIADLANIFTKNLQKTQNPNNN
Dpon AEE61901.1      FEVPDATVEAFTPRGLRVSIPDQDGIK-LFALHAKINEEM-NG-REAGT-----------------FSRDITKAKDGRWT
Dpon ENN74344.1      YQVPTPTFEAFKPKGFRVSIPDDEKVE-LFAFHGKINKPI-IQ-IEPGD-----------------FSQDVLKAVNGRFS
Dpon ENN74953.1      FEVPDATVEAFTPRGLRVSIPDQDGIK-LFALHAKINEEM-NG-REAGT-----------------FSRDITKAKDGRWT
Dpon ENN76697.1      YAVPEPKIDIFDTKGFRVSIPDGFGLE-LFAFHGKINEPM-NG-LEAGQ-----------------FSKDILMKCGDLWV
Tcas EFA01356        FEVPDALVEVFQPQGLRVSIPDQEGIK-LFAFHAKVNEEM-NG-REGGT-----------------FSRDITKAKHGRWT
Tcas NP_001164284.1 FVIPDVTLEAYAPKGFRASIPALPGIQ-MFAFHMNVNKKI-SQ-VDPGD-----------------YRQDYTSPDGNVWS
Tmol BAC99308        FEVPDALVEVFRPRGLRVSIPDQEGIK-LFAFHGKINEEM-NG-REGGT-----------------FSRDILKAKNGRWT
Bmox BAA92243.1      YEAPPATLEAIHPKGLRVSVPD-EGFS-LFAFHGKLNEEM-EG-LEAGH-----------------WSRDITKPKNGRWI
Pxyl AFK24449.1      YEVPPAKLEAIYPAGLRVSIPD-DGFS-LFAFHGKLNEEM-EG-LEAGH-----------------WSRDITRPKNNRWV
Pint AAM95970.1      YVVPSAKLEAIYPKGLRVSIPD-DGFS-LFAFHGKLNEEM-DG-LEAGH-----------------WARDITKPKEGRWT
Slit ACI32819.1      FPIATPTVEAFQPKGFRVILKD-DGYS-FVGFRGNINVDFNNG-LNEGQ-----------------IHKDITHAQKGYWI
dmel AAF49244.1      YKIPTPTVELLE-TGFSVSIPDEEGVK-VVAFNVNRNRNF-TSFINEGQ-----------------YNVRLTEPQNGRWT
dmel AAF49246.3      -------------EGFEVSIPDEPGIQ-RVFYMFQIDDTC-PA-L---------------------MDYITEAVNGSWV
dmel AAF50349.1      YEVPKAKIDVFYPKGFEVSIPDEEGIT-LFAFHGKLNEEM-EG-LEAGT-----------------WARDIVKAKNGRWT
dmel CG30148        YEAPEAQVRVFYPRGFEVSIPDAEGIS-LFAFHGKLNEEF-DG-LEAGQ-----------------WSRDIPKAKRGRWT
Amel NP_001157186.1 YVPPTPSVEPLYPVGLRMSIADEAGIS-LVAYHVKFNDDF-YS-LEAGT-----------------IARDIIKPRNGYWV
Spur XP_001176934.2 YDMKNPEISLLTTGGIRFAYPDEPGIT-LVAFHYSINTPL-SG-VNVGQ-----------------YNYDVTTKTGAYFV
Cint XP_002126690.1 YSVQQPIISLMEPTGLMFMYPDDGNIN-LVSYHYSINTPL-PD-VQAGT-----------------YNQDVSESTNGYFT
Cgig BAG82629.1      LSIQPAIVEQYNGHGVKFTIPDDGNYD-FVAVHYSINQPI-AG-VGAGQ-----------------WAFDVHTKQGSSFV
```

```
Dvir GNBP-1          FRD--RETRLKKGD------------------------IIYYWLYVDYNNGRNTLGYRKTDQQYEVRE--FSN
Dvir GNBP-2          FSD--RSTKLRVGD------------------------KIYYWTYVEYGDEYEKRGYPKDDQVFTVTN--LIK
Dvir GNBP-3          FL---PIVKVRYEDGPEGPENIYQIIPNKRNSADLSKTLVDLWNNITEPNNTNTFSPTPPSESTGYDE---ST
Dpon AEE61901.1      FYD--SQAKLAVGD------------------------TLYFWTFVDYFDGERKLGFVRDDQFFTITE--LLP
Dpon ENN74344.1      YTD--PNIKLKEND------------------------VINYWIFVQ----HDQLGFRIDEQSWTVDE--LRP
Dpon ENN74953.1      FYD--SQAKLAVGD------------------------TLYFWTFVDYFDGERKLGFVRDDQFFTITE--LLP
Dpon ENN76697.1      FED--NTEKLVVGN------------------------TLYYWLFVI----RNRLGHRYDNGKFVVRD--ITS
Tcas EFA01356       FYD--PYAKLKIGD------------------------TIYYWTYVDYFDGKNKLGYTKDDQEFVVRQ--LLD
Tcas NP_001164284.1 YFN--SDLSLNIGD------------------------TVNYWIFVQ----HEKLGYRKDNVEWTVTE--LLQ
Tmol BAC99308       FYD--ANARLKEGD------------------------ILYYWTYVDYFDGKNKLGYPNDDQKFVVKQ--LLD
Bmox BAA92243.1      FRD--RNAALKIGD------------------------KIYFWTFVI----KDGLGYRQDNGEWTVEG--FVD
Pxyl AFK24449.1      FSD--KQARLKIGD------------------------KVYFWTFVI----KNGLGYRQDDGVWTVEG--FVD
Pint AAM95970.1      FRD--RNVKLKLGD------------------------KIYFWTYVI----KDGLGYRQDNGEWTVTE--FVN
Slit ACI32819.1      YRN--REVKLNIGD------------------------TLYYWLFVQ----KNGKGQFLTELAYTVTQ--FVN
dmel AAF49244.1      TNF--SSVPLRSQD------------------------VLYLWTSVQ----HQKAVYQDLAQPLPVCN--LGG
dmel AAF49246.3      SK---QKMSLQNND------------------------KLQISMLVQ----FNEEIFEKSETRVIINTRLLTT
dmel AAF50349.1      FRD--RITALKPGD------------------------TLYYWTYVI----YNGLGYREDDGSFVVNG--YSG
dmel CG30148        FRD--HKTKLNHGD------------------------TLYFWTYVI----YNGLGYRQDEGAHVVTS--Y--
Amel NP_001157186.1 YED--RSTRLKLGD------------------------IIYYWIHVV----YNGLGYNLLDQKHVVNE--FYN
Spur XP_001176934.2 HEN--TEVDVKKGD------------------------VVNYWVYVN----YYGPGYQLLEQSWTASE--APA
Cint XP_002126690.1 LQN--FNVAVVPGD------------------------EVNYWVNVI----TSTGGYLLTDQTWVAQG---PT
Cgig BAG82629.1      HTNDLPAIQVHKGD------------------------TVYYWLHAQ----KGGTPSELLGQSAVIGD--LTT
```

Figure S3. Multiple alignments of *D. v. virgifera* GH16 family protein sequences. A. GH16 family proteins identified from the *D. v. virgifera* transcriptome. B. The active site region sequences. GNBP sequences are boxed. The catalytic nucleophile and proton donor residues are highlighted with magenta and green, respectively (based on Viladot *et al.* 1998, *Biochemistry* **34**: 11332). C. The N-terminal conserved domain sequences of GNBPs. Residue shown to be within hydrogen-binding distances and involved in hydrophilic interaction with lamitrihexaoses on *Plodia interpunctella* and *Bombyx mori* proteins are highlighted with yellow, and Arg's involved in binding of triplex β-glucan are highlighted in light blue (based on Kanagawa *et al.*, 2011, *J Biol Chem* **286**: 19158).
