# Supplementary material for: Molecular Evolution of Glycoside Hydrolase Genes in the Western Corn Rootworm (Diabrotica virgifera virgifera)
Source: PLoS One. 2014 Apr 9;9(4):e94052. doi: 10.1371/journal.pone.0094052 (PMC3981738; doi:10.1371/journal.pone.0094052)
Supplement: Figure S4 — Multiple alignment of the potential GH5 family protein sequence identified from D. v. virgifera with four fungal GH5 proteins (A) and the maximum-likelihood phylogeny including other known GH5 family proteins (B). The potential amino acid residues for the catalytic nucleophile and catalytic proton donor are highlighted with magenta and green in the alignment, respectively (based on Larsson et al. 2006, J Mol Biol 357∶ 1500). Coleopteran proteins included in the phylogeny are found in Table S5. The D. v. virgifera sequence is shown in red. The GH5 protein sequences are classified into subfamilies according to Aspeborg et al. (2012, BMC Evol Biol 12∶ 186). Bacterial, plant, fungal, and nematode sequences are indicated by brown, green, cyan, and grey. The numbers at internal branches show the bootstrap support values (%) for the maximum-likelihood and neighbor-joining phylogenies in this order. Only bootstrap values higher than 70% are shown. (PDF) [file pone.0094052.s004.pdf]

A

```
Dvir GH5      ------------------------------------------------------------------------------------------------------------------------
Y. lipolytica MSLQLLIDETGNFTDPSGKAVILRGINVAADAKLPAKPFTPSQQKA-GDDFYD--TTVSFVGSPFPLEEADEHFARIKAWGFNTIRYIYTWEALEHEGPGVYDEEFIDYTIAVLRKIGE-
V. dahliae    -----------------------------------------------TD-----------------------------------------------------------------------
A. gossypii   MLGKIYISQQGEFTDYEGNVVQLRGVNLDPSVKFPQQPRIPTNMPV-DDEFWDGATNVSFVNERLDPKEIEEHMIRLKALGYNCIRYLFTWEALEHGGPGIYDEEYMKYTVMVLKKIKEA
S. cerevisiae MPAKIHISADGQFCDKDGNEIQLRGVNLDPSVKIPAKPFLSTHAPIENDTFFEDADKVSFINHPLVLDDIEQHIIRLKSLGYNTIRLPFTWESLEHAGPGQYDFDYMDYIVEVLTRINSV

Dvir GH5      ------------------------------------------------------------------------------------------------------------------------
Y. lipolytica -HGMFAFMDPHQDVWSRFTGGSGAPLWTLYAAGLDPRHCMTTHSALVQNLWDNP----------SKFPKMIWSTNYQKLACQVMFTLFFAGNHFAPKCIINGVNVQDYLQGSFLAAKRHL
V. dahliae    ------------------------------------------------------------------------------------------------------------------------
A. gossypii   -GGMYVYLDPHQDVWSRFSGGSGAPLWTLHCAGFQPKRFLATEAAILHNYYIDSETQAE----KAQYPEMIWSTNYYRLACQTMFTLFFSGKLFAPKCVINGRNIQDYLQGHFLKAVMTF
S. cerevisiae QQGMYIYLDPHQDVWSRFSGGSGAPLWTLYCAGFQPANFLATDAAILHNYYIDPKTGREVGKDEESYPKMVWPTNYFKLACQTMFTLFFGGKQYAPKCTINGENIQDYLQGRFNDAIMTL

Dvir GH5      ------------------------------------------------------------------------------------------------------------------------
Y. lipolytica AERIA--VDQHLVENVVIGWESVNEPNHGLIGYENIHAIPDSQKLRLGPTPTAFECMRMGMGETVEVDNYEFGPFGATKNGTVVIEPKGTLAWL--KDFSECDKIYGWTRGPEWLPGMCI
V. dahliae    ------------------------------------------------------------------------------------------------------------------------
A. gossypii   YKYIQDNAPELFEENCIIGLETMNEPNCGYLDHPNLRELPRDRQLMKGTTPTAYQSFILGEGFACNIDSYDISLIGARKIGKSFVDPKGKSAWLDATERLELDRAYGWTRPDDWAPG-CI
S. cerevisiae CARIKEKAPELFESNCIIGLESMNEPNCGYIGETNLDVIPKERNLKLGKTPTAFQSFMLGEGIECTIDQYKRTFFGFSKGKPCTINPKGKKAWLSAEERDAIDAKYNWERNPEWKPDTCI

Dvir GH5      ----------------------------------------------------------------------------DDDDVA-QFDITSRVVYAPHWYDGLTLLNKRWN-FFNIDYLGVK
Y. lipolytica WAQHGVWEPKT----GKLLKPTYFNDGHSFHGIGSKIDEEVWVNKYFLGYWLAFLATIRQVNKDWLVLMQAPVMQVPPDLVNHPEFNDKRIVYSPHYYDGLTLMNKKWNRLYNVDVVGIL
V. dahliae    ----------------TLLKKDYFKKNPK---TGLEYTFPNWTQTHFMDGYRRYRDAIRAIHTDCIMIMQYPTLELPPKIKG-TEDDDPKMAFAPHWYDGITLMTKKWNKLWNVDVVGVL
A. gossypii   WRLHGVWDIESKSSKPVLLLPGYFSKCPS---TGEETSMSYFTNKLFLDFYVRYRNQYRELDPDSLLFLEPPVLQEPPYLIG-SDIIDKRTVYACHFYDGMSLMFKSWNRRYNVDTFGFM
S. cerevisiae WKLHGVWEIQN-GKRPVLLKPNYFSQPDA---T-------VFINNHFVDYYTGIYNKFREFDQELFIIIQPPVMKPPPNLQN-SKILDNRTICACHFYDGMTLMYKTWNKRIGIDTYGLV

Dvir GH5      RGRYPNYAMAVKIGDKAIRECFRNQLAWIKEEGQGAIGQH-PTVIGEIGIPYDMNGGKSYRSNG--------------------------------------------------------
Y. lipolytica RGKYPSIVLGLRVGESAIRNCLRDQLRFLRKEGLAKIGNF-PCLISEIGIPYDMDDKYAYRTGDYSQQIRALDANQYALEGSKLH-YTLWVYTASNNHKWGDNWNGEDLSLYSKDDA---
V. dahliae    RGRYWTPALAVKVGETAIRNCFRDQHNYLYKEGKEHLGNH-PCIMTEFGIPYDMDDHYAYKTGDYTSQSAAMDANYFGVEGSGMEGHCLWLYT--NTHEYGDQWNGEDLSIFSHDDKLLP
A. gossypii   RGKYLSPIFGLVFGEANIKRCFRRQLRAMKLEGRRFLGDSVPIFFTEIGMPYDMEGKKAYRDHDYSSQIGANDALGFALEGSNMS-FSLWCYTYINNTTWGDNWNREDFSIWNKEYA-MK
S. cerevisiae NKKYSNPAFAVVLGENNIRKCIRKQLSEMQKDAKSMLGKKVPVFFTEIGIPFDMDDKKAYITNDYSSQTAALDALGFALEGSNLS-YTLWCYCSINSHIWGDNWNNEDFSIWSPDDKPLY

Dvir GH5      ------------------------------------------------------------------------------------------------------------------------
Y. lipolytica ----AKQLQKYGGATQTLTNGSADGSQSSEETPPPTYTSYASYYLDSSYLGKTSIGKSIKGRVSSIKGAIRRRNKTAAVPLSSHGDAFKPPPEYVLGARAGEAFIRPCPQVISGKLDSYG
V. dahliae    TSPAAAAPGPQGE-----------------------------------------------------------------------------------------------------------
A. gossypii   VPRDVVVKTGDAMPNSSINTIVGAESH----------------LTCESRLSDDA----LVLDYS--------------------------------GFRALDAILRPYPVKIHGSFSTAE
S. cerevisiae HDTRARTPTPEPSPASTVASVSTSTSKSGSSQPP-------SFIKPDNQLDLDSPSCTLKSDLS--------------------------------GFRALDAIMRPFPIKIHGRFEFAE

Dvir GH5      --------------------------------------------------------------------------------------------------------
Y. lipolytica FDLQKSVFTLKIKGAACGENDKCEGKLLPTTIYLPHYHFLQWATGVSTSSGKWEYDEN-TQILTWWHYEGPQQLQV---KGNIRFITDYIDTANNLSSSQCRSQ
V. dahliae    -------------------------------------------------------------------------------------------------------F
A. gossypii   FDLERKRYFLEIIARTETEGTT--------SIFLPYYHFPPESTVVSSSSGYYVREQDNNQLLKWCHGGGRQYISIEVTGMGSSYSVQSADSS-------CVIM
S. cerevisiae FNLCNKSYLLKLVGKTTPEQIT-----VPTYIFIPRHHFTPSRLSIRSSSGHYTYNTD-YQVLEWFHEPGHQFIEI-CAKSKSRPNTPGSDTSNDLPA-ECVIS
```

B

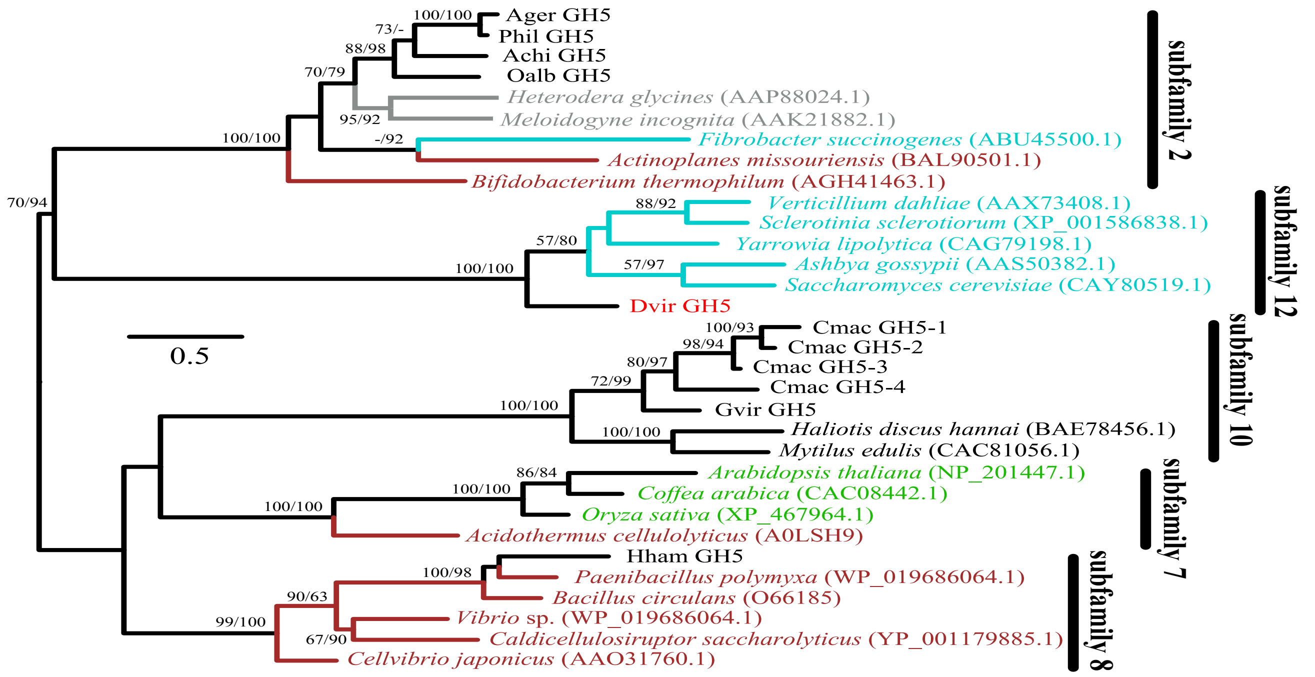

Figure S4. Multiple alignment of the potential GH5 family protein sequence identified from *D. v. virgifera* with four fungal GH5 proteins (A) and the maximum-likelihood phylogeny including other known GH5 family proteins (B). The potential amino acid residues for the catalytic nucleophile and catalytic proton donor are highlighted with magenta and green in the alignment, respectively (based on Larsson *et al.* 2006, *J Mol Biol* **357**: 1500). Coleopteran proteins included in the phylogeny are found in Table S5. The *D. v. virgifera* sequence is shown in red. The GH5 protein sequences are classified into subfamilies according to Aspeborg *et al.* (2012, *BMC Evol Biol* **12**: 186). Bacterial, plant, fungal, and nematode sequences are indicated by brown, green, cyan, and grey. The numbers at internal branches show the bootstrap support values (%) for the maximum-likelihood and neighbor-joining phylogenies in this order. Only bootstrap values higher than 70% are shown.
