# Supplementary material for: Molecular Evolution of Glycoside Hydrolase Genes in the Western Corn Rootworm (Diabrotica virgifera virgifera)
Source: PLoS One. 2014 Apr 9;9(4):e94052. doi: 10.1371/journal.pone.0094052 (PMC3981738; doi:10.1371/journal.pone.0094052)
Supplement: Figure S5 — GH1 family gene sequences identified from the D. v. virgifera transcriptome (A) and the maximum-likelihood phylogeny of GH1 family proteins (B). In the alignment, the labels for partial sequences are shown in italics. Potential residues for the catalytic nucleophile and the proton are highlighted with magenta and green, respectively (based on Marana et al., 2001, Biochim Biophys Acta 1545∶ 41; Scharf et al. 2010, Insect Biochem Mol Biol 40∶ 611). In the phylogeny, labels for the coleopteran species belonging to the superfamily Curculionoidea, D. v. virgifera, and other beetle sequences are shown in olive, red, and orange, respectively. Their species abbreviations are found in Table S5. Arthropod, other metazoan, nematode, fungal, plant, and bacterial sequences are indicated by black, purple, grey, cyan, green, and brown, respectively. The numbers at internal branches show the bootstrap support values (%) for the maximumlikelihood and neighbor-joining phylogenies in this order. Supporting values are shown only when higher than 60%. The scale bar represents the number of amino acid substitutions per site. (PDF) [file pone.0094052.s005.pdf]

A

```
GH1-1  M--------------------KTILI-----VSICCSVI-------------------------------------------------------------
GH1-2  M--------------------EYTFC-----LLFLWVGR-------------------------------------------------------------
GH1-3  M--------------------KLFIL-----ICLIALCF-------------------------------------------------------------
GH1-4  M--------------------KVFLF-----ALLLGVCL-------------------------------------------------------------
GH1-5  M--------------KLVGCFLVFLS-----VLALGAAD-------------------------------------------------------------
GH1-6  M--------------------DKVL------VIAVFSFF-------------------------------------------------------------
GH1-7  M--------------------------TANHFFYFLFLV-------------------------------------------------------------
GH1-8  M-------------AYQLLRKCEDTMCKVIYFLVLLNYL-------------------------------------------------------------
GH1-9  M-----------------MSPKHLTLLRYLIYICLTFNL-------------------------------------------------------------
GH1-10 M---------------------------------------------------------------------------------------------------
GH1-11 M---------------------VRALHILATLLALKLSYGNPQPGYPVDPHCPPEDCEPYPVHCPNPIPEPVCEIPVIPPPHYPHPIPDCDYPYPGYPHG
GH1-12 M-------------------TSPVLI-----FILVHLSN-------------------------------------------------------------
GH1-13 M----------------LLNFRVLIL-----TVSFSNIL-------------------------------------------------------------
GH1-14 M--------------------LCIKF---IAVIFFILNL-------------------------------------------------------------
GH1-15 M--------------------KVSLL-----LLLVVSSV-------------------------------------------------------------
GH1-16 M--------------------KVILI-----SLLLGLSL-------------------------------------------------------------
GH1-17 M---------RSDLQTMAPVPKIFFL-----IFVVVSSY-------------------------------------------------------------
GH1-18 M------------------FRHRVWV-----LCVLVISA-------------------------------------------------------------
GH1-19 M------------------ISKELW------LLCIVLST-------------------------------------------------------------
GH1-20 M---------------------KIYV-----LLLIVCTS-------------------------------------------------------------
GH1-21 M------------------TLKYMLI-----TFLLVGIT-------------------------------------------------------------
GH1-22 MIGSFIYKSFGFELKFEFKSFKMMWI-----YMVFGFLL-------------------------------------------------------------
GH1-23 ----------------------------------------------------------------------------------------------------
GH1-24 ----------------------------------------------------------------------------------------------------
GH1-25 ----------------------------------------------------------------------------------------------------
GH1-26 M---------------------KMKFTIFTSFLVVAVFA-------------------------------------------------------------
GH1-27 ----------------------------------------------------------------------------------------------------
GH1-28 ----------------------------------------------------------------------------------------------------

GH1-1  ----------------------------------------------------------------------------------------------------
GH1-2  ----------------------------------------------------------------------------------------------------
GH1-3  ----------------------------------------------------------------------------------------------------
GH1-4  ----------------------------------------------------------------------------------------------------
GH1-5  ----------------------------------------------------------------------------------------------------
GH1-6  ----------------------------------------------------------------------------------------------------
GH1-7  ----------------------------------------------------------------------------------------------------
GH1-8  ----------------------------------------------------------------------------------------------------
GH1-9  ----------------------------------------------------------------------------------------------------
GH1-10 ----------------------------------------------------------------------------------------------------
GH1-11 PINPGPLPPHLPLPPHLPIPKPCDGLPGAWPNEWPCYFPCGWPDDWPCDFPLGLPNNWPIDFPHGLPHDWPNNFPHGWPVPHGYPNEWPCEWPDNWQEYW
GH1-12 ----------------------------------------------------------------------------------------------------
GH1-13 ----------------------------------------------------------------------------------------------------
GH1-14 ----------------------------------------------------------------------------------------------------
GH1-15 ----------------------------------------------------------------------------------------------------
GH1-16 ----------------------------------------------------------------------------------------------------
GH1-17 ----------------------------------------------------------------------------------------------------
GH1-18 ----------------------------------------------------------------------------------------------------
GH1-19 ----------------------------------------------------------------------------------------------------
GH1-20 ----------------------------------------------------------------------------------------------------
GH1-21 ----------------------------------------------------------------------------------------------------
GH1-22 ----------------------------------------------------------------------------------------------------
GH1-23 ----------------------------------------------------------------------------------------------------
GH1-24 ----------------------------------------------------------------------------------------------------
GH1-25 ----------------------------------------------------------------------------------------------------
GH1-26 ----------------------------------------------------------------------------------------------------
GH1-27 ----------------------------------------------------------------------------------------------------
GH1-28 ----------------------------------------------------------------------------------------------------
```

```
GH1-1  ----------------VLR----TS--TAKEEFPKDFLFGIASSAYQIEGGYNADGKGESIYDYYTKTKHARFYNNSNGNIACDSYHKWRADVRLLKDLG
GH1-2  ----------------TTS----AK--LPMYDFPKKFSFGVATSAFQIEGGWNASGKGVSIWDHLVHDTNEIIVDGSNADVACDSYNKVDYDILLLKALG
GH1-3  ----------------SAN----AKDPINNRRFPKTFKVGVANAAPQIEGAWNTDGKGETIWDHFAHTQPEKFIDRSTPDVACNSYYQYKEDIAMVKEMG
GH1-4  ----------------AQN----AD--TNPRRFPASFKLGVANAAAQIEGAWEEDGKGEHIWDRWSKVRPEMISDRSTPAVAADSYHKYKEDVRLIKEMG
GH1-5  ----------------GDDEFANYT--ITNRKFPKGFTFGVATAAYQIEGAWNLDGKGEQVWDTFFHEHPDRVDDGTNGDVATDSYHLYKEDVRCMEEVG
GH1-6  ----------------TTC----FA--DNDRTFPETFSFGAATAAFQIEGAWNENGKGESIWDRFIHTNQSRVVDGSNADIACDSYHKYTEDVALAAKMG
GH1-7  ----------------TYA----QA--KNNGSFPKDFLFGVATSAYQIEGAWDEDGKGENIWDHMVHQVPSPIKNNDTGDIACDSYHKYKEDVDILEDLG
GH1-8  ----------------RFS----DA--KNNGTFPANFLFGVATSSFQIEGAWNEDGKGESMWDEYNHRVPSPIRNGDTADIACDSYHKYKEDVKLVADLG
GH1-9  ----------------VAA---ESQELHYNRKFPDDFLFGAATSAYQIEGAWNVDGKGESIWDTYVHRVPSPIKNGDTGDIACDSYYKYKEDVKLAADLG
GH1-10 ------------------------------------------------------ENRGENIWDHAIKANPKIIEDGSNASSADESYTHYEDDFKALAQMG
GH1-11 PDCWPNYWDGCDYPYPAGSVPAIPPPVESCDVFPKEFSFGVASGAYHIEGAWDQDCRSESIWDRWLHTCPSVVLDGSNGDISADAYNHIKGDIELLNNLT
GH1-12 ----------------NVA----TS--INYNRFPSDFLWGASTSAFQFEGAWNEDGKAPSVWDYFLHNQPYFVPDGSNADVSANAYHMVETDVELAKSLN
GH1-13 ----------------AED----YV--INNKRFPADFMFGVATSAYQVEGGWNEDGKGVNIWDYFTHRNSSVIRDKNNGDIACDSYHRIKEDIAILKDLG
GH1-14 ----------------GFL----DA--LNNKKFPPDFIFGCATAAFQIEGAWNEDGKGPSVWDTTNHKIPSVIEDNSTADVSCDSYHKYKEDVALLKELG
GH1-15 ----------------YST--------PDSLPFPEGFLFGVSTAAYQVEGGWNQDGKGENIWDHQLHTNSNITIDHSNGDVACDSYNRIKRDVDILNNLD
GH1-16 ----------------AED-----P--INNKKFPDDFLFGVASSAYQVEGAWNEDGKGESLWDHRVHTDPNFIEDNSTGDVACDSYHKIKEDVAILKELG
GH1-17 ----------------ADD----YQ--INNKRFPSNFMFGVGTSSLQIEGAPNEDGKTETMWERYIADHPGYVADGSDVSVACDSYHQYKRDVAMVKELG
GH1-18 ----------------TCG----EE--LNNKTFPGYFIFGTATASYQVEGAWNEDGKGQNIWDNFTHEDPSRIFGNANGDVACDSYHKYKEDVALLKDLG
GH1-19 ----------------SLA----TT--INVSKFPKDFMFGTATASYQVEGAWNEDGKGENTWDEVTHRVPSPIVDNSTGDVACDSYHKYKEDVAMLKHLG
GH1-20 ----------------KLV----EP--LNNRRFPAHFMIGAATASYQVEGAWNKDGKGENIWDRLTHSPNSPVLGNQNGNIACDSYHKYKEDVAMLKHLG
GH1-21 ----------------VCD----TE--LNKKRFPKDFMFGVATSSYQIEGAWNEDGKGESTWDHFVHQTPSPIVNNYTGDVACDSYHKMKEDVALLKYLG
GH1-22 ----------------SNA----IA--DDNTKFPEGFTFGVATSAYQIEGAWDEDGKGENNWDRVAHTIPNFIYHNDSGDVTCDSYHQWEEDIRILKELK
GH1-23 ----------------------------------------------------------------------------------------------------
GH1-24 -----------------MS----NS--EDQKRFPEGFLFGTASSAYQIEGGYNVDGKGESVYDYYTHKTPEKFHNGSNGDVACDSYHKWQEDVHILKDLG
GH1-25 ----------------------------------------------------------------------------------------------------
GH1-26 ----------------KTT----QS--LNTKKFPPKFMFGTATAAYQIEGGWNEDGKGENNWDHLSHSPLSPIYDHHTGDVACDSYHKYKEDVQLLKHLK
GH1-27 ----------------------------------------------------------------------------------------------------
GH1-28 ----------------------------------------------------------------------------------------------------

GH1-1  VQFYRFSISWSRVLPKGY-SNKVNPD--GLRYYNDLINELKKNGIEPMVTLYHNDLPMPLQE-IG-GWTNPYTAYYFEDYARYMFSYFGDRVKYWITM--
GH1-2  VSHYRFSIAWTRILPNGF-RNHVSED--GVKYYNYLINKLLENGIEPWVTIFHFDIPYGMFL-IG-GMFNEKFSEYLSDYADLLFNLYGDRVKTWVTINE
GH1-3  LDHYRLSIAWSRILPTGY-LDSLNQL--GVQYYKNVFQELKKNNIEPLVTLYHWDLPQPLQDQMN-GWINETIIDIFADYCKLCFELFGDDVKWWITINE
GH1-4  LDVYRLSISWARIYPTGYPTAEPNAA--GVQYYKNLLAECKQNGIQVMATLYHWDLPQQLQDDFG-GWLNETVVDLFAAFADVSFKLFGDDVTWWITINE
GH1-5  VDYYRFSIAWSRILPDGT-LDNVSQE--GVNYYVNLFKELRAKGIKSFVTLYHWDIPTALEK-QG-GWRNPKVVDWFQDYAEFCFKTFGEYVDSWVTINE
GH1-6  LTHYRFSISWSRLLPTGF-ISPVNLI--AISYYQDLIRELEKYKIEPVVTIYHWDLPQMLQE-LG-GWENSKIVDYFVDYADLVFASF-PSVKHWITFNE
GH1-7  VNFYRFSISWSRILPTGY-PSTVNKA--GLEYYKNLTKELVSRNITPVATIFHWDLPQPLSE-ID-GWSNPRLADLFVEYSRIVIQGL-PDVGVWITFNE
GH1-8  ADFYRFSISWPRILPTGY-TDNINQK--GIQYYQNLVKEILKYNMVPIATLYHWELPQTLVA-SNLDWVNPDIVDIFVNYTRVVIQNL-PDVGLWITVNE
GH1-9  IKLYRFSISWPRVLPTGK-ADNISEA--GLNYYKNLVKEIRNYGMIPVATIYHWDLPQKLYD-EGIHWSNVSLVPYIVDYARIVIKAL-PDVGYWVTINE
GH1-10 ITHYKLSISWSRMIPTGY-TDNIDDMSNGTTHYQALISELVKQKITPVVVLYNWDLPQPIQD-YG-GWTNESNIDAFVMYARKVFK-YCSEAKYFITFDN
GH1-11 VRHYVFSISWPRIIYDG--CGEVNPY--GIEHYRKLIKLLKLNHIEPIVVLYYGDLPQALQD-QG-GFLCPSFVEWYTEYARVCFECFGDDVKYWITFHK
GH1-12 LKVFRTSVAWTRILPQGN-EGKPNQI--AIDHYKNLFRLLKENDIEPLVTMSHFDLPQKLSL-YG-GFLNESLVEWFTNYARICFDNFGDDVKQWATFNE
GH1-13 VSHYRFSISWPRILPNGF-RDEVNQK--GIDYYKNLIKELKDNNIEPLVTLYHWDLPQFLQD-LG-GFFSSSLEVWFAEYARICFEAFGKDVKYWVTFNE
GH1-14 ATHYRFSISWPRILPNGF-NDYINPL--GLAYYKNLIAELKANNIEPLVTISHGDLPQVLGD-IG-GYYNESFPDWIRDFARVAFDEFGDDVKYWFTINE
GH1-15 VDHYRFSISWSRILPTGL-DDNINEA--GVNYYKKLINELKIVGIQPMVTLLHFDLPQKLQD-MG-GFLNESIVDWFGNYSRVAYRLFGDDVKFWITINE
GH1-16 VNHYRFSLSWTRILPTGL-ADEINVG--GVIYYKNLISELKANNIIPLVTIYHWDLPQVFQD-YG-GFLNSSIVDWYSNYARVVFSLFGDDVKHWITINE
GH1-17 VTHYRFSLAWTRILPNGV-NDYVNPL--GVQYYKNLIKELKANNIEPVVSIFHWDIPQVLQD-MD-GFLNDSIVDWYTDYARVCFEEFGDDVKYWATFNE
GH1-18 VDHYRFSISWSRVLPTGY-SNEVNEA--GVNYYKNLIKELNDNDIFPLVTMFHWDTPQPLED-IG-GWTNDTIIDRFVDYAQFLFETFGDDVQMWLTFNE
GH1-19 VTHYRFSLSWSRILPTGF-NNKVNSL--GIAYYKNLIKELRANNIEPLVTIFHWDTPQPLEN-LG-GWTNELIVDRFVDFAKVAFENFGDDVTYWLTFNE
GH1-20 VNHYRFSISWSRILPTGF-TNRINQA--GITYYKNLIKELKDNGIEPFVTIFHWDTPQPLED-IG-GWTNELIVDKFRDYAKVLFDNFGNDVKYWVTFNE
GH1-21 VKHYRFSLSWSRILPTGY-TNKINQA--AIKYYKSLIRELTDNGIEPVVTILHCDTPQALED-MG-GWTTDWIIDRYVEYAAVCFENFGADVKYWLTFNE
GH1-22 VTHYRFSISWSRLLPHGF-NFEVNQA--GVDYYTNLIKALKAANIEPYVTLFHWDLPQFLEA-LG-GWPEREIVQWYSEYARLCFELFGDDVKHWMTFNE
GH1-23 --------------------------------------------------------------------MNEWVVDRFVEYVEVCFKNFGEDVQHWITFNE
GH1-24 VDFYRFSISWSRILPTGY-SNKINEE--GVKYYENLIDGLLKNGIQPMITLYHWDLPMPIQE-LG-GWTNIKTAEYFEDYARIVFERFGNKVKYWITIN-
GH1-25 ----------------------------------------------------------------------------------------------------
GH1-26 VNHYRFSISWSRILPSGF-DNKINPL--GIQYYKNLIKELKDNGIEPLVTMHHWDTPQPLEN-IG-GWTNEKIVDMFADYARILYENFGDDIKYWTTFNE
GH1-27 ----------------------------------------------------------------------------------------------------
GH1-28 ----------------GY-TDNINQK--GIQYYQNLVKECLKYNLIPVATLYHWEIPQSLFA-AKLDWTNPAFIDIFVNYTRVVIQNL-PDVGLWITINE
```

```
GH1-1   --EMSSNGYGD-DKYPPFINQ-PGIANYICQHVMLLAHAKAYHLYDAEFRKNQQGKIGVAVHATWYIPATLS-KDVEAAERAREFKIGSIMNPIFHTDGN
GH1-2   PYSVCVSGTRVLGEYRP-----NGIVEYLCGHTFLKAHAAVYDLYQRKYKSQQNGKISMAFNLQCNYPETNSAEDIEAAERANQFALGWFVHPVV--FGN
GH1-3   PKQVCQAGYGS-GAFAPGIVS-NGIMDYVCTKNVLLAHAKAWHIYDEQFREKNKGQVAMVIDATWYEPGSDNQEDKDAAERAQQFDIGIYGNPLF--NGD
GH1-4   PKQVCHAGYGD-GYFAPGVVS-SGVGEYTCARNVLLAHAKAYHNYNDNYKASQGGKVSMVIDTLWFEPGSDSDADKDAAERLLQFGFGLYGNPIF--NGD
GH1-5   PKQICHAGYGV-GGYAPGVVS-PGIGEYVCARHVLLAHAKTWRLYDRSYRQQLKSRITIVIDSDWYEPETESEADETAAEHKRQFVYGMYANPIV--YGN
GH1-6   PKQVCRSGYGK-GNMAPGIAR-SGTADYMCSYHVIKAHAAAYHKYQDRYK-SLGGKITMALDGVWSIPYWDNEEDRQAAERQLNFEFGLYAHPIF--FGD
GH1-7   PKQICHYGYGV-GTVAPAIHS-SGLREYWCAYTVLNAHAKTYHMYKS---LNLKAPMGITIDCEWYEPLTDSVQDKQAAKRNLNFECGLYAHPLF--IGD
GH1-8   PKQICHHGYGE-GKFAPGRHN-SGVDDYQCDYVVLKAHAKAYHMYKEEF-PHYKALMSLTIDCEWYEPLTDSKEDMNAARRNIDFECGLYSNPVY--NGD
GH1-9   PKQVCRIGYGA-TMLAPALNS-SGLLEYDCAYVLVKSHAAIYRMYKKEF-PDYKAKMSIVIDCQWIEPQTNAPKDKDAAERQLQFECGLYFNPIFN-NGD
GH1-10  PRAICKNGYGD-GSLAPFSSN-SGLDDYECARVLLKAHAAVYKVFKEEFASKNGAKISIALDASWSIPASNNEADKEAAERRNLFEFGLYADPIFK-TGN
GH1-11  PTVICKDGYGQ-GTFAPGINDGPGNYEYICGHNLLKAHAAAYHLYDDYYRPCQNGKISITLNSDWYEPQSCCDTDLCASETKLQFQIGWFAHPIY--LGD
GH1-12  PYVWCVGAYDV-GYFAHSETR-PGFWGYTCGHNLLKAHAAVWHMYDKEFRPTQKGTVSLTIAGDWHIPKTSSIEDKEAAERQLQFKVGWFVHPLY--HGN
GH1-13  PSSICNNGYGK-GKFAPGIYN-PGIGEYLCIHNLLNAHAAAYHVYDKEFRSTYNGKVTTVHSISYCQPKTNTTEDIEAADQKMQFNLGWMADPIY--FGD
GH1-14  PHEVC-------------IYD-GAEKVYDCAANLLRAHAKIWHMYDEEYRSKQHGKVAMVLNLNWYEPETDKTDDIIAAETKMQFSWGLFGHALY--HGD
GH1-15  PLTICYYGYGT-GAVPPFIQS-EGILEYQCLRNLLKAHARAYHVYAEEFRNKQQGKLSMSFNTNAFLPGSDNPKDIEATERVQQFNLGVYAHPIF--VGD
GH1-16  PQQICSGGYGY-GYFPPTIKS-EDLLEFQCIHNLVKAHAKAWHIYDDEFRSTQKGVISITLDTSSYIPASNKSEDIEAADRMQHFTLGVYANPIY--VGD
GH1-17  PKTYCNGGYGY-AYNPPQIPS-QGLLEYVCSHNLLRAHAKTYRMYEQEFRPRQGGKIAIVLDTYWYRGATDSEEDKTAAATALHFDLGWYANPVF--NGD
GH1-18  PKQTCNGGYGN-GILAPGIQS-PGVGEYLCTHNVIKAHAAVWHLYDEKYRRRQNGHLGITLDCFFGEPDSDSQEDKDAAERMLQFNYGWYANAIV--NGN
GH1-19  PKQICNEGYGN-KQKAPLVDS-PGIGEYLCAHNLLKAHAKAWHLYDEQFRSTQKGFVGITIDTAWMEPDTDSTEDVDAAERLQQFNHGWYARPLL--LGD
GH1-20  PKQTCQEGYGS-GQKAPAIKS-HGIGEYLCAHNVIKSHAKVWHLYDRYYRKEQRGMVGIVLDTMWMEPDTESELDKVAAERKLQFIFGWYANPII--NGD
GH1-21  AFYMCTLGYGT-GTQAPGRKA-SGIGDYICGHNIIKAHAKAFHLYNDTYRPVQKGSVGISLVSIYAEPKSDSDEDKEAAERMIYFNFGWFASPIM--FGS
GH1-22  PKQTCTNGYGS-GSYAPAIKS-PGVGEYMCIHNLIKAHAAAYHIYDTEFRDKQNGKVGIVIDSAWWEP-YNATTDSDASERLLHFTFGMYANPIF--YGD
GH1-23  PVLMCIMGYGI-GVIAPGRKT-GGVGEYLCAHNVIRAHAKVFHKYQEAYNPTQKGKIGISLVTAWNDPRTDKPEDIEAANRLMLFTFGWFAHPLV--FGS
GH1-24  ---TALLGYAD-SEFPPYINQ-PGISNYMCIYVTLLAHAKAFRLYHNEF-KQYKGQVGIVVDGRWYQPGSDSDKDVEATNRALEFEIGIWLHPLLSPKGD
GH1-25  ----------------------SGIGEYLCTHNVLKAHAKTWHLYDKHFRSTQKGKVGITIDTAWLEPDTNSTRDVDAAERGQQFVHGWYTRPLT--LGD
GH1-26  PKQTCQQGYGS-GLRAPAIKS-HGIGEYLCTHNLLKAHAKAWHMYDDEYRQKQHGFVGITLDSMWMELDTDSELDKAAAERAIQFTFGWFANPII--KGD
GH1-27  ----------------------------------------------------------------------------------------------------
GH1-28  PKQICHQGYGE-GTLAPGLLL-SGLEDYQCDYIVLKAHAKTYNMYKKEF-PHYQAHMSLTIDCEWYQPYTNSKEDIDAARRNLDFECGMYSHPLY--YGD

GH1-1   YPALVKEMVDNRS-ISEGYLQSRMPTLTFQEADYIQGTYDFAGLNIFTSYRVKHDA-KGT--ANTTLEKDANVTLHQDTEWEKSGSDWLRVVPIGVRRVL
GH1-2   YPQVMIDSIANIS-KTQGFLESRLPVLTAEDQKLLKGSFDFIGLNNYDTYLVSD---DRKVDVDPSYINDQGVNFTYFPNWTVDYS--IEMRSKGLGIIL
GH1-3   WPEIVKERVAYRS-KLEGFTESRLPAFTEDEIKYIKGTSDYLGLNHYSTLLTNHTA-DAPI-GTPSFDNDKSVLLWHRDDWTQGSADWFFDVPWGLRKFL
GH1-4   WPKLVKDRVAMRS-EKEGFKQSRLPAWTQEEIDYIKGTGDFVALNHYATHMANGTN-EAPI-GNPSFGSDISAMEWARPEWPKGDGDWFSIVPWGLRKLL
GH1-5   WPQIMIDNVADFS-KKQGFKESRLPAFSEEEIELIKGTYDYLAVNHYTSLMVKARA-EPTL--IVSWDDDAGVDAYQKSTWETAAIGWFKKVPWGFGKLL
GH1-6   WPQVVKDRVNYRS-KMENYPESRLPEFTAEEMKYINRTADYVAFNFYNTKLIKDID-EASF-DITSFDNDLRVKDDVDPRWTI-AMDGNTIYPQGLRSYL
GH1-7   WPEDVKMRIYERS-MAENYNTSRLPELTLAQISYIYGTTDFFGLNYYVTFTVADGK-EGPT-NVTSYDNDVRIVLGNVSN-ADIDVTGFPIVPLGLTKVL
GH1-8   WPSSVKERVKLRS-QLQGYNRSRLPEFTTEEINYIKGTSDLYLLNVYFAYLVKDVP-EAPS-NVTSFRSDVKAELIRFPG-TSVGINGMPIVPWSVENVL
GH1-9   WPKVVKDRVDFRS-LKANLTTSRLRAFTEEEKIYNKGTYDFLGLNHYYTMLARDEAEEAPF-NETNYENDVGTVDSFDPKWVVESNGLFVIVPGGVFKML
GH1-10  WPQVVIDRTDLRS-KGEGAQKSRLPIFNQTEKDAIKGTFDFMAIEFFDTKLVADDT-EAKF-ETSSYDNDLRVKTSNDPNWTV-DADEHAIAPGSLRNLL
GH1-11  YPEVMIERIRLNC-DYNGYYNARLPVFSHAEIEYIKGTHDFFSLAYDSAFYVTACV-DNYW-GNICYESDVGVQLVGCEG---------EFLTCGIRSLL
GH1-12  YPSAMIETIKNISINEEGRNSSRLPEFTEEEIEYIKGTNDVFYLNTYHVYHVSNVI-KK---LSPSFDNDIRGTIEEIPDG----------AAWGIRSFI
GH1-13  YPEIMKTRIATRS-KLEGFIESRLPEFTEEQKERLKNTSDYFAVNSYDSYFAFAIP-EPAIVLPPSLDADSGVGGTKEG---------IGDGSFGMGKLL
GH1-14  WPPIMKSRIGLRS-KLEGYPKSRLPEFTKEEIEYIKGTNDFLAVNSYTTSIIRAID-EPEI-GDPTLEKDIGVYEYQRDDWDSSGSIWLKVVPWGMRKLL
GH1-15  WPEIVKTRVAIRS-KLEGRNESRLPAFTPEEIDYIKGTADFLGLNTYTAQLVLDTGEEPPLGDHPTKEHDALITTFQDDKWEDTNVPTVKVVPQSIKPLL
GH1-16  YPEIVKTRVAERS-KAEGREQSRLPTFTQEEIEYIKGTHDFFGLNVYTGSLVADLGYDPPVQNPPSKWQDVAVNNYYPPEWENTTILYTKVVPWSARYLV
GH1-17  YPEIMKSRIAARS-AAEGRAQSRLPEFTDAEKAALKGSADYFALNTYTGNLVKSIP-DPPIFNIPSRYQDVGVLTYTDPKWESSSLDWLKVAPWGIVDLL
GH1-18  YPKVMRNVIARRS-AAQGLSKSRLPDFTSKEIQFIKGSFDFLGLNYYTVNMVKARG-SEVP-VVSSWEKDMEVDIYLKEEWPKSSSSWLRIVPWGMRKML
GH1-19  YPEAMKKTIAERS-ALQGFSQSRLPEFTQDEIEYLNGTVDYLGLNYYTTVMATNAA-DKRI-DVVSWEADVEVNTYQKEEWPTSASSWLRVVPWGLRKTL
GH1-20  YPAQMKQFIAKRS-IAQGFKQSRLPQFTPEEVKYIKGSIDFLGVNQYTSMLVRNMD-DKYK-TEVAWEADSETYLYQPDNWESTATDWLKVTPWALRKLL
GH1-21  YPYQMEITINERS-VVQGFAQSRLPSFTEEEKAYIAGTVDYFGVNYYSSVVAENLP-DFKD-LDVSWEADVEVIPSQDPHWENTTTPQFSVYPDGLYKLL
GH1-22  YPEVVKSSVARRS-QSQGFPNSRLPEFTAEEQNTIKGTIDFLGLNQYSSFYVTDKT-DDK--DGMGYDADAEVKTWADESWQQGAADWLNKAPFGIRNML
GH1-23  YPQEMEKTVSNRS-DAQGYSKSRHTGFTEEESAYIKGTVDYFGINYYTSVLISHYD-DLHQ-NESGWAADSQVIQYQPPDWEDTVVNMFKVYPHGIYKLV
GH1-24  FPEIVKERIAAIS-MAEGYRQSRLPKFSTTDIDLLKGSVDFVGLNLYTSHLVEHVE-DEHF-KGTSTIRDEGIRLYQDASWKGSQSTWLKVHPEGARKVL
GH1-25  YPELMKKTIAKRS-SLQGFSQSRLPQFTKDEIEYLKGTLDYLGLNYYTTFMARDSD-DEKI-DDISFEADAQISAYQKDEWPKSATPWLRVVPWGLRK--
GH1-26  YPWQMKQFIARRS-LAQGFSESRLPEFTAEEIKYIKGTIDYLGVNQYTAGLVRHVE-DKNR-TKISWEADQETYNYQPEHWEKTAANWLKSVPWSMRKLV
GH1-27  --------------------------------------------------------------------------LIRFPG-TSVGINGFPIVPWSIENML
GH1-28  WPPSVKARVKFRS-ELEGYNKSRLPEFTTEEINYI-----------------------------------------------------------------
```

```
GH1-1  KWVKDKYD-DPEIFITENGFPD-KGE---INDVKRIQYLQKYLKEVSISIQEDGVKLKGYTVWSFLDSFQWT------EGYREKFGLYHVNFSDKNRKRT
GH1-2  NWIKKTYN-DPEIRILEQGFPE-TST--DLYDHNRHKYIVASLQQLWKSINEDNVNVTAYTFWSLMDNFEWD------KGYSVRYGLNYVDFNDPNRIRI
GH1-3  NWLKNTYG-NPEIVITENGFAD-TNG--TLEDDNRITYLEGHVSACLDAIYEDNVNLTAYTVWSIMDDWEWT------GGYTSFLGMYKVDFNDPNRARI
GH1-4  VWLKDTYN-DQEIIITENGLSD-NTG--VLEDDHRVDYFRDYISNCLDAIYEDNVNLSTYIAWSIIDDWEWG------GGYKSFLGMYKVDFNDPERPRI
GH1-5  RWIKKTYG-DIEIVISENGVSD-RSG--TLQDEHRIKFIQAYMSHMLDAIH-DGVNVTAYTLWSIIDNFEWT------QGFNGKLGIYYVNQSDPSLPRI
GH1-6  KWISENYN-SPEIIITENGIAD-NGT--SLEDSERISYLSDYLNAVLDSIYEDKVNVTGYTMWSLLDNFEWT------SGYSMRFGFYSVDFEDSNRTRA
GH1-7  NHIKTEYN-VPKILITEIGISD-NG---TLEDESRIKTLKNYFSAILDAVYDHDVNVIGVTIWSLMDNLEWL------KGYSAHFGLYSVDFTDDNRTRT
GH1-8  KFIKDEYK-DPIILISEVGTSE-DGT--SLEDDMRIDFYEKAFNAMLEAMYNYDVKVIGTTVWSLLDNLEWT------DGYAAHFGLYHVDFNHPNRTRT
GH1-9  KWIKQNYG-DQDIIITEIGMSD-NGT--YLNDKDRIDFYTEYFCNILEAM--DEVKVKGIILWSLMDNFEWN------SGYTAHFGVYHVDFQNPNRTRT
GH1-10 KYIRDNYN-NPEILITASGISD-DNG--TYNDDHRVAFLADQLSDVADAINEDKVNVFGYTYWSLLDSFEWT------KGMKPHFGLYAVNL-ELETSRV
GH1-11 GWISRNYY-NPSVFITNDGYAS-CSR--DCYDCDRIEYIKAILRNVRLSMIEDYTRVYGYTYYSYIDGFEWNLVDGRLGGYTLKYGLYDVEFDCPQKTRT
GH1-12 SWIRKNCN-NVPIFITENGKGSTDYT--GLQDDYRIDFMQKMFSSIRDSMEIDNATILGYTFWSLYDGFEVQ------YGYRKKMGLAYIDFNSNNKTRI
GH1-13 LWIKDRYN-NPDMIITENGLMT-QTE--TLEDDDRISMVKNCLSRFRDVM--DQVNLLGYTLWSVMDNFEWT------RGYTAKYGIYHVDFNNSNRTRT
GH1-14 NWFKQEYF-NPEILITENGYSD-FTG--QLNDPNRTHYLTEYLSAIKDAMDYDGVNVIGYNVWSLIDNFEWN------AGYTVKFGLVNVDMNDPNRTRT
GH1-15 KWIKDQYN-NPGVFITENGYPS-PAGDMYLKDDRRVHYIESYLRYIRESMEEDEVSMLGYTVWSLMDNFEWI------FGYTVKYGLYDVDFTSPDLTRA
GH1-16 NWIKDKYN-NPGIIVTENGYPS-YGD----KDDRRIYYIRGYLSALRDAMEKDNVSILGYTVWSIMDNFEWT------SGYGQKFGLYEVDFSSSNRTRT
GH1-17 NFIKNNYN-DPDIMVTENGLCD-KGG---LDDPRRVNYHKLYLSYIHDAMINDQVKVFGYTAWSLMDNFEWA------SGYTQKFGLFSVDFSSPNRTRT
GH1-18 NWMKKTYGDDTEIFITENGVSD-DGS--SLDDQIRIDYYQQHISGVRDALD-DGVNVIGYTAWSLMDNFEWI------RGYSECFGLYQVNFTDADRPRT
GH1-19 NWIKNTYG-DIPIIITENGISE-DGT--SLEDDTRIDYYQQHLSAVKDAMD-DGVNVFGFTAWSLMDNFEWL------RGYSEDFGLYHVDFSSPDRTRT
GH1-20 NWIKQTYG-NIPQIITENGYSD-NGT--TLEDDGRINYYKQHLSNIRDAID-DGVNVIGYTTWSLMDNFEWF------RGYSEKFGLYHVDFTSPQRTRT
GH1-21 KWISNNYN-NISIMITENGYPD-KGE--TLQDDERISYIQRHLSKVRDALD-EGVQVLGYTFWSLMDNFEWL------MGYDTKYGLFEVDFNSPDRTRT
GH1-22 KWIKKTYP-DIPVIITENGWSD-FDG--TLDDDPRIDYISSYLSNVKASMDEDKVNVVGYTVWSLIDNLEWL------FGFTNKFGLYRVDFNDPNRTRT
GH1-23 KWVSKTYN-NIPIIITENGYPD-DGS--TLDDNKRISYIKRHLSKLRDALD-EGVNLIGYTYWSFMDNFEWL------SGYSVKFGVIAVDFNSPNRTRT
GH1-24 NWVKETYN-NPVVFITENGFSD-DGE---IEDNNRIEYLQGYLTNILKAIYEDKVDVKGYAVWSLMDNF-------------------------------
GH1-25 ----------------------------------------------------------------------------------------------------
GH1-26 NWISKTYG-NIPQFITENGYAD-NGT--TLEDDRRIHYYQETLSNLRDAMD-DGANVIGYTAWSLMDNF-------------------------------
GH1-27 KFIKDEYK-DPVILVTEIGTSE-DGS--TLEDDMRIDYLEKTYNAILEAMYNYDVKVVGVTVWSLLDNLEWM------DGYTARFGLYHVDFNDPNRTRT
GH1-28 ----------------------------------------------------------------------------------------------------

GH1-1  PKLSATWYKKVIKERRVV-------------------------------------DWKEVTSNLEEDL*-------------
GH1-2  PRLSAITYNHVSTKKTLR------------------------------------------------------------GLL*
GH1-3  KRKSADYFTNVVKNRCLV-------------------------------------EESQCVD*-------------------
GH1-4  KRKSADYFTQIVKNRCLV-------------------------------------DADKCVD*-------------------
GH1-5  AKDSSKYYANVIKTKCLV---------------------------------------DSCVDN*------------------
GH1-6  AKTSVDYYSQIIKNRKIPE*--------------------------------------------------------------
GH1-7  AKASAEYYKNVIKTKSIEDNSHSD-------------------------------GGQSITKVNGYVVGFIIVSFYFFKLW*
GH1-8  PKKSATYYKNVINTRRLLRAD----------------------------------NGSNINKISGFMTIVLLFPILWNTLM*
GH1-9  PKESAGFVKKLSKDKTLSCGNPSKYRSVPMALPKWTPNANVSIWNNGTGNRNHTNPGTTPTPLRNNNNGNGNGNRGKNKDC*
GH1-10 EKKSAQYYALAIKERKPVDPADVETTTTSTTTEGH--------------------QGGAGNRYQPVVPVIFIISLISYVLQ*
GH1-11 ARSSAAYYQHLASTGCIDNPC----------------------------------PIPYCYDYNYDYYHY*-----------
GH1-12 PRKSAYYYSNVSRTGCISNSRCVYVSSCD--------------------------GYTFPMSSIMTAITVHILAILTLCRS*
GH1-13 PKKSAEWYKHVIQTRCLT---------------------------------------DICVD*-------------------
GH1-14 KKDSFYYYQKVCQTHCIV---------------------------------------DQCID*-------------------
GH1-15 AKPSAQYYKEVILTRCAT--------------------------------------IVGCFND*------------------
GH1-16 PRPSAMFYKNVIATRCLV---------------------------------------ETCDD*-------------------
GH1-17 IKQSGKFYQNVCHTRCLL-------------------------------------DEDQCADY*------------------
GH1-18 PKRSVEFLKKVMTTRCVT---------------------------------------DVCEDY*------------------
GH1-19 PKKSVEYLKNVIQYRCVL-------------------------------------GNSTCDD*-------------------
GH1-20 PKKSVSYFKHVAETKCIV-------------------------------------DVKDCVIDVDEIVRYIYTNVDKNVLA*
GH1-21 PKKSADYVKKVYESRCLLE------------------------------------DSSECYD*-------------------
GH1-22 AKKSAKWYTNVIENRCLG----------------------------------------TCE*--------------------
GH1-23 PRKSAKYLKNVYATRCIME------------------------------------DNSQCSD*-------------------
GH1-24 ----------------------------------------------------------------------------------
GH1-25 ----------------------------------------------------------------------------------
GH1-26 ----------------------------------------------------------------------------------
GH1-27 PKKSVGYYQYVASTKRLLSAD----------------------------------NGSNLINISGFMTIVLLFPILWNTLM*
GH1-28 ----------------------------------------------------------------------------------
```

B

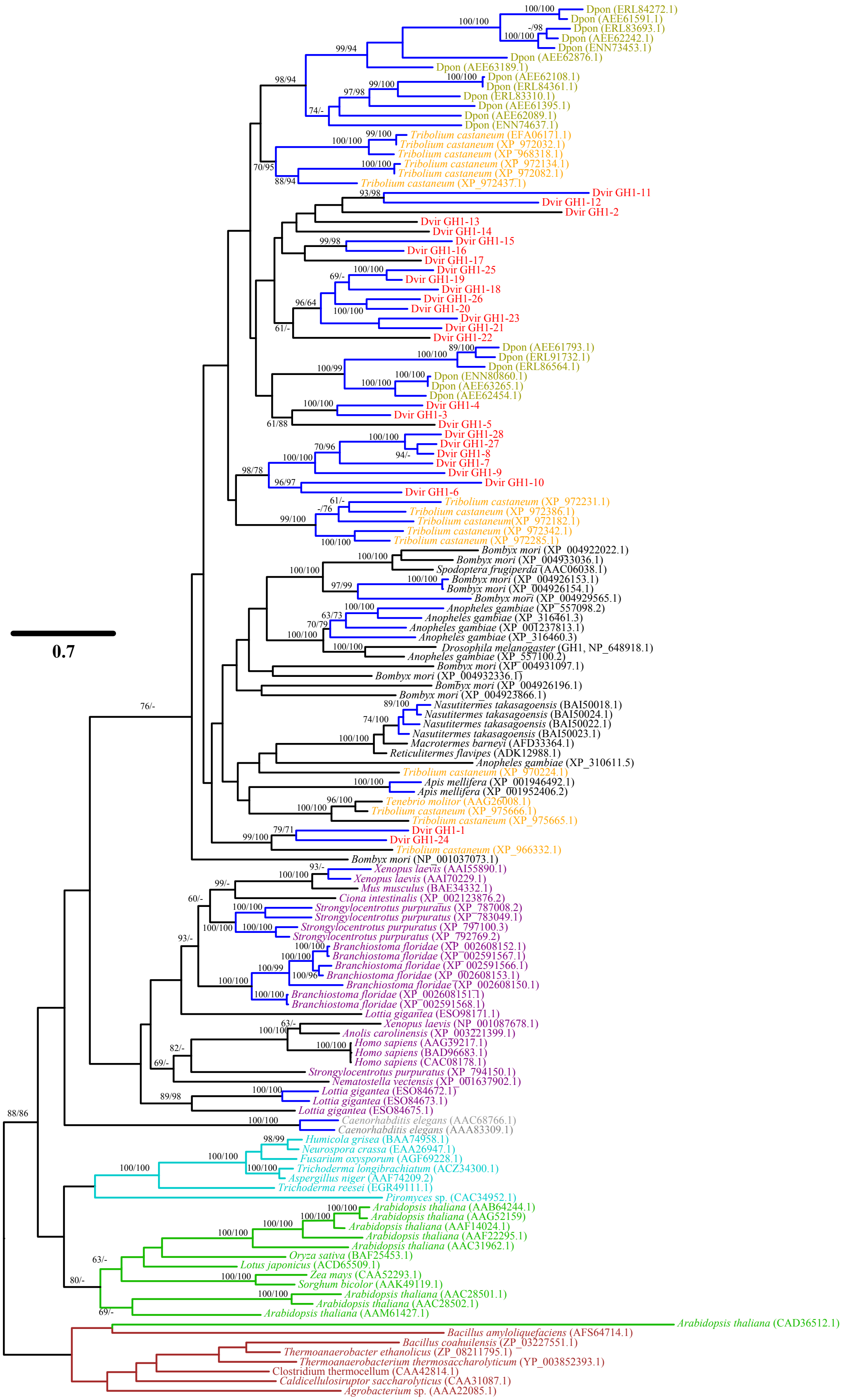
0.7
Dpon (ERL84272.1)
Dpon (AEE61591.1)
Dpon (ERL83693.1)
Dpon (AEE62242.1)
Dpon (ENN73453.1)
Dpon (AEE62876.1)
Dpon (AEE63189.1)
Dpon (AEE62108.1)
Dpon (ERL84361.1)
Dpon (ERL83310.1)
Dpon (AEE61395.1)
Dpon (AEE62089.1)
Dpon (ENN74637.1)
Tribolium castaneum (EFA06171.1)
Tribolium castaneum (XP_972032.1)
Tribolium castaneum (XP_968318.1)
Tribolium castaneum (XP_972134.1)
Tribolium castaneum (XP_972082.1)
Tribolium castaneum (XP_972437.1)
Dvir GH1-11
Dvir GH1-12
Dvir GH1-2
Dvir GH1-13
Dvir GH1-14
Dvir GH1-15
Dvir GH1-16
Dvir GH1-17
Dvir GH1-25
Dvir GH1-19
Dvir GH1-18
Dvir GH1-26
Dvir GH1-20
Dvir GH1-23
Dvir GH1-21
Dvir GH1-22
Dpon (AEE61793.1)
Dpon (ERL91732.1)
Dpon (ERL86564.1)
Dpon (ENN80860.1)
Dpon (AEE63265.1)
Dpon (AEE62454.1)
Dvir GH1-4
Dvir GH1-3
Dvir GH1-5
Dvir GH1-28
Dvir GH1-27
Dvir GH1-8
Dvir GH1-7
Dvir GH1-9
Dvir GH1-10
Dvir GH1-6
Tribolium castaneum (XP_972231.1)
Tribolium castaneum (XP_972386.1)
Tribolium castaneum(XP_972182.1)
Tribolium castaneum (XP_972342.1)
Tribolium castaneum (XP_972285.1)
Bombyx mori (XP_004922022.1)
Bombyx mori (XP_004933036.1)
Spodoptera frugiperda (AAC06038.1)
Bombyx mori (XP_004926153.1)
Bombyx mori (XP_004926154.1)
Bombyx mori (XP_004929565.1)
Anopheles gambiae (XP_557098.2)
Anopheles gambiae (XP_316461.3)
Anopheles gambiae (XP_001237813.1)
Anopheles gambiae (XP_316460.3)
Drosophila melanogaster (GH1, NP_648918.1)
Anopheles gambiae (XP_557100.2)
Bombyx mori (XP_004931097.1)
Bombyx mori (XP_004932336.1)
Bombyx mori (XP_004926196.1)
Bombyx mori (XP_004923866.1)
Nasutitermes takasagoensis (BAI50018.1)
Nasutitermes takasagoensis (BAI50024.1)
Nasutitermes takasagoensis (BAI50022.1)
Nasutitermes takasagoensis (BAI50023.1)
Macrotermes barneyi (AFD33364.1)
Reticulitermes flavipes (ADK12988.1)
Anopheles gambiae (XP_310611.5)
Tribolium castaneum (XP_970224.1)
Apis mellifera (XP_001946492.1)
Apis mellifera (XP_001952406.2)
Tenebrio molitor (AAG26008.1)
Tribolium castaneum (XP_975666.1)
Tribolium castaneum (XP_975665.1)
Dvir GH1-1
Dvir GH1-24
Tribolium castaneum (XP_966332.1)
Bombyx mori (NP_001037073.1)
Xenopus laevis (AAI55890.1)
Xenopus laevis (AAI70229.1)
Mus musculus (BAE34332.1)
Ciona intestinalis (XP_002123876.2)
Strongylocentrotus purpuratus (XP_787008.2)
Strongylocentrotus purpuratus (XP_783049.1)
Strongylocentrotus purpuratus (XP_797100.3)
Strongylocentrotus purpuratus (XP_792769.2)
Branchiostoma floridae (XP_002608152.1)
Branchiostoma floridae (XP_002591567.1)
Branchiostoma floridae (XP_002591566.1)
Branchiostoma floridae (XP_002608153.1)
Branchiostoma floridae (XP_002608150.1)
Branchiostoma floridae (XP_002608151.1)
Branchiostoma floridae (XP_002591568.1)
Lottia gigantea (ESO98171.1)
Xenopus laevis (NP_001087678.1)
Anolis carolinensis (XP_003221399.1)
Homo sapiens (AAG39217.1)
Homo sapiens (BAD96683.1)
Homo sapiens (CAC08178.1)
Strongylocentrotus purpuratus (XP_794150.1)
Nematostella vectensis (XP_001637902.1)
Lottia gigantea (ESO84672.1)
Lottia gigantea (ESO84673.1)
Lottia gigantea (ESO84675.1)
Caenorhabditis elegans (AAC68766.1)
Caenorhabditis elegans (AAA83309.1)
Humicola grisea (BAA74958.1)
Neurospora crassa (EAA26947.1)
Fusarium oxysporum (AGF69228.1)
Trichoderma longibrachiatum (ACZ34300.1)
Aspergillus niger (AAF74209.2)
Trichoderma reesei (EGR49111.1)
Piromyces sp. (CAC34952.1)
Arabidopsis thaliana (AAB64244.1)
Arabidopsis thaliana (AAG52159)
Arabidopsis thaliana (AAF14024.1)
Arabidopsis thaliana (AAF22295.1)
Arabidopsis thaliana (AAC31962.1)
Oryza sativa (BAF25453.1)
Lotus japonicus (ACD65509.1)
Zea mays (CAA52293.1)
Sorghum bicolor (AAK49119.1)
Arabidopsis thaliana (AAC28501.1)
Arabidopsis thaliana (AAC28502.1)
Arabidopsis thaliana (AAM61427.1)
Arabidopsis thaliana (CAD36512.1)
Bacillus amyloliquefaciens (AFS64714.1)
Bacillus coahuilensis (ZP_03227551.1)
Thermoanaerobacter ethanolicus (ZP_08211795.1)
Thermoanaerobacterium thermosaccharolyticum (YP_003852393.1)
Clostridium thermocellum (CAA42814.1)
Caldicellulosiruptor saccharolyticus (CAA31087.1)
Agrobacterium sp. (AAA22085.1)

Figure S5. GH1 family gene sequences identified from the *D. v. virgifera* transcriptome (A) and the maximum-likelihood phylogeny of GH1 family proteins (B). In the alignment, the labels for partial sequences are shown in italics. Potential residues for the catalytic nucleophile and the proton are highlighted with magenta and green, respectively (based on Marana *et al*., 2001, *Biochim Biophys Acta* **1545**: 41; Scharf *et al.* 2010, *Insect Biochem Mol Biol* **40**: 611). In the phylogeny, labels for the coleopteran species belonging to the superfamily Curculionoidea, *D. v. virgifera*, and other beetle sequences are shown in olive, red, and orange, respectively. Their species abbreviations are found in Table S5. Arthropod, other metazoan, nematode, fungal, plant, and bacterial sequences are indicated by black, purple, grey, cyan, green, and brown, respectively. The numbers at internal branches show the bootstrap support values (%) for the maximum-likelihood and neighbor-joining phylogenies in this order. Supporting values are shown only when higher than 60%. The scale bar represents the number of amino acid substitutions per site.
