# Supplementary material for: Molecular Evolution of Glycoside Hydrolase Genes in the Western Corn Rootworm (Diabrotica virgifera virgifera)
Source: PLoS One. 2014 Apr 9;9(4):e94052. doi: 10.1371/journal.pone.0094052 (PMC3981738; doi:10.1371/journal.pone.0094052)
Supplement: Figure S6 — GH31 family gene sequences identified from the D. v. virgifera transcriptome (A) and the maximum-likelihood phylogeny of GH31 family proteins (B). In the alignment, the labels for partial sequences are shown in italics. In the phylogeny, labels for the coleopteran species belonging to the superfamily Curculionoidea, D. v. virgifera, and other beetle sequences are shown in olive, red, and orange, respectively. Their species abbreviations are found in Table S5. Arthropod, other metazoan, nematode, fungal, plant, and bacterial sequences are indicated by black, purple, grey, cyan, green, and brown, respectively. The accession numbers shown in parenthesis are from NCBI except for: BGIBMGA012077-PA and BGIBMGA013995 from SilkDB (http://www.silkdb.org), DPOGS202361 from MonarchBase (http://monarchbase.umassmed.edu), and Px016165 from Diamondback moth Genome Database (http://59.79.254.1/DBM/). The numbers at internal branches show the bootstrap support values (%) for the maximum-likelihood and neighbor-joining phylogenies in this order. Supporting values are shown only when higher than 60%. The scale bar represents the number of amino acid substitutions per site. (PDF) [file pone.0094052.s006.pdf]

A

```
GH31-1  MKIRDFIKSFGIRKGRQISTTNLVSFEAF--------------------------------------------------------------YDDRGNKFP
GH31-2  MA--------------------------------------------------------------------------------------------------
GH31-3  MERSD-----------------------------------------------------------------------------------------------
GH31-4  MA--------------------------------------------------------------------------------------------------
GH31-5  MDRKKYRNATKYNKVPQEIPKIVLAAPSTENLSLLDSEMDSKSRNDNNSSPDKTDDVIQRDTVRPPMRRKNSISMPNLDDLKVFIEQEGDGYDTSTEKIN
GH31-6  ME--------------------------------------------------------------------------------------------------
GH31-7  M---------------------------------------------------------------------------------------------------
GH31-8  MK--------------------------------------------------------------------------------------------------
GH31-9  MS--------------------------------------------------------------------------------------------------
GH31-10 MT--------------------------------------------------------------------------------------------TFIQQQ

GH31-1  DIKESCTKSNPSQNEEYIGGYVRFKETSEAKPVFRP-------------------------------------------------SLPIILVIFILISVF
GH31-2  -------------------------------QN----------------------------------------------------LLYISLILFIVLYAS
GH31-3  -------------------------------KM----------------------------------------------------LRPLLLLALIASYVS
GH31-4  -------------------------------EM----------------------------------------------------IRYAILLVVLATTAA
GH31-5  TTIQEEPEDPEFQNISSYPRSVRRKSVLSPRMLKPPEDTELGGSPSNSITSVNSIASLLKEKIQNLPQTFRKKKSPEYKTKVFVGLLFTTIIVLIVTAYF
GH31-6  ----------------------------------------------------------------------------------------LKICFNVLTL-L
GH31-7  ----------------------ILK-------Y----------------------------------------------------LLAIAIAINFATAKY
GH31-8  ------------CTTTSSAVYSIIKGMNCWRAI----------------------------------------------------LIAILLVGVNQV--L
GH31-9  --------------------------KGCHKLF----------------------------------------------------TFLIFLTTLHSIRGI
GH31-10 NELREDEDETATASVKWTVWTILCKTTIVGWFF----------------------------------------------------TFAFALIVPVLTYYL

GH31-1  IILPIIYLVNCFRFTFDNQDFYHRNAPAIYSDTSTKEPKDQTTKRPWKLHDLPVPPAPDYNQCKLLTDLDKFDCFPEDGANAEKCEARGCCWIPRNKSVL
GH31-2  AV---------------------------------------------------------------------------DKNNFKTCEQSSFCRRLRS-VKP
GH31-3  CA---------------------------------------------------------------------------NHTIYKDCSRIPFCKKLR--TIE
GH31-4  AI---------------------------------------------------------------------------DKTVFKSCSERPFCNRLRNYVVD
GH31-5  LY--------------------------------------------------------------------------------------------QQKVLA
GH31-6  VF--------------------------------------------------------------------------------------------------
GH31-7  TY--------------------------------------------------------------------------------------------------
GH31-8  VQ--------------------------------------------------------------------------------------------------
GH31-9  VV--------------------------------------------------------------------------------------------------
GH31-10 VV--------------------------------------------------NLGTYKEVDTFTCFVNSTFRVPCGKVNISEHECVKLACCYDKPTDSCY

GH31-1  ----SQAPLDVPYCFYPPNYNTYKVVNVTETSFGLIAFWRRQYRTAYPDDVDELK-MIVKYETETRLR-VKFVDPSS-----------FRFEPPYPEV--
GH31-2  GE--SKYELDLESLEITDNSVESKLLN---------------------TEAGILFKFSLTAISGNIFR-LQVDEASPLYARYRPQF-ALRGEPQVAKLKL
GH31-3  NH--SKFTAQLDKLSQTGNVISIPLTN---------------------SDSQELI-LQISLLVHETAR-IKIAEKGS--KRYQLAD-VLAEEPQTLEISA
GH31-4  DK--SKYSADVYSASIKDNILTVPLKN---------------------ENGNSLN-LLVSMFEGNKIR-VKVEDPDS--KRFELGDIVIIEELKELPLVS
GH31-5  KVYFGNMKLNKAK-------RTVKIFN---------------------DEGEDVVKLHLGTTLNYDNVLNCLPADNR-----------------------
GH31-6  --SFGEAKIDLKP---TTNGLSISVFN---------------------DDNEVTLQGTIGFGIDFTVS-DCQNHYTQ-----------------------
GH31-7  --KLGDTEIDFTA---TSNGIEIYIRD---------------------AEIGSQLYGTIGVG---KNA-SCIDFPR------------------------
GH31-8  --CSTVNEIILKP---SSNGLAIEVNQ---------------------KE-EKKLKGTIGVGIDFTNI-NCYGQES------------------------
GH31-9  --KNGNITLQLEP---KSNGISFAAYD---------------------GK-IKKLEGILGRGINFSNV-DCIGTQV------------------------
GH31-10 HYLPSRYSYDLAGSSYKASRSSSPFNT---------------------TAVEEMQ-ISVNEISINKVS-IILHKPS------------------------

GH31-1  ----------PIVDKASLALSYIIQIN---------TFKSGFKVLRKSDN------------------VTIFDSNNFLNLI------------YSNQ---
GH31-2  LE---RNKEYVSI-----EYNNNKVIV---------HARP-FKIEVFDKDELISVVNGRGLFKFEHFRKKPEQNNEGEQQQEVAEDPGTWEENFKSHHDS
GH31-3  KT---SERE-VSIAPTNASYSYKVVVT---------TGTS-FKVEFYYDD------------------NTKEIALNGEQLV------------FDKEDN-
GH31-4  DTLDGTDGS-LLVTPKDESLPYKVVID---------AGPP-LNITFYY-G------------------GLKQVVLDGERLV------------YESTDES
GH31-5  -------------DDGSLCLEWMHRARLYMNFFDLGSDVKCYNVQWIALS-------------------------------------------ESL----
GH31-6  ----------CAFNGSSLSIN---------------EVDSGYQIVWNSVY-------------------------------------------TDG----
GH31-7  ----------CKVGDNDVAFRF--------------LPGTSLSVISTTNN-------------------------------------------TQE----
GH31-8  ----------CQVGDADFSVK---------------QSEDGFHIKWETNN-------------------------------------------LTS----
GH31-9  ----------CVINDSNFSVE---------------PISAGFRLKWKTFN-------------------------------------------TRR----
GH31-10 ----------TSIAENTVNVREYVVKK---------AAEK-LMVEIFRPN------------------GDRIFSTAKGPLI------------ASEN---

GH31-1  ------MLQISSKL----PSKYIYGIGEHRSNL-LLSTE--------WSKFTLFNHDMVP----------TDNKNLYGSHPFYLMIENSTKSHGVFLLNS
GH31-2  KPKGPSAVGLDISF---PGALRVYGLPEHADRL-PLRTTGPGG----VDPYRLYNLDVFE-------YELDSTMAIYGAIPV-VYAHSPKRTIGAFWLNA
GH31-3  ------SFAFATEF---INAKRLYGLHHHPVKL-ELPSTRDVDGKVLMDPYRNRNADNDH-------YEVGSPMAVYGSVPV-VYGHSENQTSGIFLHNA
GH31-4  K-----AFTFKADF---PEANRLYGLLDHAWSL-ALGDTNNGTAES-GDPLRLRNSDSWG-------YEANSPMALYGARPV-VYGHSANKTSGIFLHNA
GH31-5  ------APTDCFDM----SHSHWYGGGQTAENAWPLEKGSHF-----YQPFITGNIETHEWGNVLKRYFINS------------------KGAAIVVDNE
GH31-6  ------EFVDKFQL--NSGNSYWFGGPERYLQN-PLEKLVLNG----NDPYVIKKADNFA---VAERYWLNS------------------KGTFIFIDDK
GH31-7  ------VLKDCFFTAFPSASTQWYGGPERRVQTWPLQHMRISQ----SSPYISRKDDNHA---VAERYWLNS------------------LGIYIYLSEE
GH31-8  ------VFQDCFDF---EEGVHWYGGPERKKQSWPIEKLEIES----YQAYVLHQLDNFA---VAERYWLNS------------------KGLYIYLNSK
GH31-9  ------AFQDCYDL---TEGVQWYGGPTIFSLIWPIQTNEIDG----SSPYVLDNGKGMP---YAERYWLNS------------------KGSYIFLDDD
GH31-10 ------YWEWTVHL----TDHSLFGLDKT-----LLQRHKNST----ISKVFYKNKNDHS------------------NFPV-IWAYHRGQFHGLTVKHD

GH31-1  NAMDVILQ---------------------PTPAITFRTIGGI--FDFYFFLGPTPS--------DVISQYTDLIGRPYMP-----PYWGL--GFHLCRFG
GH31-2  AETWVDINNSKDGNVMSSIVNLVSGSKSENNVDVHFMSESGV--ADLFILLGPTPK--------AAVKQYASLTGVHPLP-----QYYTL--AYHQCRWN
GH31-3  AEQWVDIWYDEES----------------TSGWSYFMVESGT--LDLFILLGNQPK--------NVVRQFTKLTGVAHMP-----QLWTL--GYHQCRWS
GH31-4  AEQWIDASSGSEG----------------KDPSVYFIVERAA--FDLFILLGPTPK--------EIVRQFTGLTGKAHLP-----QIWAL--GYHQCRFS
GH31-5  TPLYISIRDSP-KKEFCLRAQ--------YDNFAFVNKFTSTAQLNYSICTSANMSQLHVFLSEHTLWDGLKKEDSNIIDYFLTEPVWEM-PDMKEALTQ
GH31-6  VPLFIDQNNNE-NGALLLIAKCI------NP---YVNRQRNI--LKYDLVTKKNAKEAHL----HAVNNYLGKPTGYPNENMIRKPIWTTWPKYKQPVDQ
GH31-7  NPLFVSQNEDDSKDQVCFISKVQ------SP---YINRDQNI--LEYVIYFSDDPKTAHL----NAVNTYLGKPNGYPNPKMIKEPIWTTWAKYKAPITD
GH31-8  VPLYVDQNISN-KNRVCFIAKLE------GP---YINRHKNF--LEYDIVIKDDPREAHV----HAVKTFLGKPSGYPDERMITEPIWTTWAKYKTKISD
GH31-9  VPLYVDQNKLR-NNSVCFKSYIR------DPFHAYFNRTRNV--LQYYLIFKDDVKQAHL----HAVNKFLGKPKDHPNEAMVAEPIWTTWAKYKTKIND
GH31-10 GPLEVSILT--------------------SNLIILKGLLGET--IELVLYTGPTPA--------DLHQQHLEDSEQLDV------PEWLL--KTHMCRKN
```

```
GH31-1  YKTLNNTRHV---MQRNIDAGIPLDTQWNDLDYMNDSNDFTYNQVNFKD-LPSFVRELHSRGMHYIPLIDPGVSASEKPGTYPPYDVGIEMDIFIKNSSG
GH31-2  YNDEDDVTTV---VQTFDANDMPVDFMWLDIEYTNGKKYFTWDPIKFPH-PEIMVNNLTATGRKLIVIIDPHYKRESG---YFVHEDCLANDYYVKTKDN
GH31-3  YKTQEDTKFV---VAEMNANNFPMDAIWLDIDYTDGKRYFTWDPETYSD-PEELQRNLSSTNKKLVVILDPHIKVDED---YPVYTGA-KGKYFVKKPNG
GH31-4  YKSQDDVKDV---VARLDENDFPWDAIWLDGDHTDDYKWFHWNHTTYTD-PVELQQNISATGRVCVSISDPHIKVDDD---YDVYAGA-KGKYFTKWANG
GH31-5  DVIDNVTTKITNSVAVLKQGHILINEFW-----QEQIGDFELDQSRFPD-FDKLIEKLKRRGFRVVFTIQPFISTESF----NFAEV-VRKKLLVSERFS
GH31-6  NKVNEFVKSIVDH--NFLGGQIEIDENW-----EVCFGSHVFNKDKFPD-PNGLIDSIKSQGFKTALWINPFVNNDCQ----NYSNEGLDKGYFVKDVNG
GH31-7  DIVIEFAKSIREN--GFENGQLEIDDAW-----EKCYGAQEFSEEKFSK-ISDTVKQLKDMDFRVTLWVHPFVNEDCK----YRSDEGIQGDFFVKGVNG
GH31-8  EIVLDFAKDIRDN--GYEKGQIEIDDYW-----EKCYGAQEFTPTTFPD-ITNTIKTLKSWSYRVTLWIHPFVNSDCQ----DNSKIGLEKGYFVLDQNG
GH31-9  SVVLKFAKDIRNH--GYEKGQIEIDDNW-----ERCYGAEQFS-HNFTN-IKDTVKILKSINFRVTLWIHPFVNDNCN----NNSQIGLEKGYFVIDKYG
GH31-10 EKFLNVSTLVSNFVLDSEADSFCIDENL-----LMGILAERANDTSYQDAVQALITPLREKGKKFLLSVPPQVLTNSD-----FYNNASSLDLLYTYLNK

GH31-1  Q---PFVGKVWNRV-STVWPDFTNPNVVDYWTLM---LKNLHKE-----------IQFDGAWIDMNEPSNFLSGAFNGCPNSTLETPPYFPDIDGGVINY
GH31-2  N---VYEGWCWPGS-SS-YIDFYKPEAAEYYKNLYRLENFKGTS-----------HDVY-IWNDMNEPSVF--------NGP-EITMPK-----------
GH31-3  E---DFKGDCWPGL-SS-YIDFLDPEARDYYASWYSYEKFKKST-----------PVLAGVWNDMNEPSVF--------DDELEKTLPF-----------
GH31-4  S---EYVADCWPGA-SS-WIDYLNPEAADYYSTWFSFEKFNGST-----------PTLAGIWNDMNEPAVF--------DDSTEKTMPW-----------
GH31-5  DKRIPALTRYKSLQ-SAGVLDITNVQTISWLLHK---LKKVMNT-----------YKIDSFYLDIGVA----------------YNMPH-----------
GH31-6  D---T-RAIWWESA-NAHQVDFTNPDAAKWFSDK---IKALADD-----------PGLDGFKFDAGETDY--------------AIPPH-----------
GH31-7  L---N-NATWWNGNGNGHQIDFTNPKAAEWYTDR---LKKLQTN-----------PGIDSFKFDAGEADY--------------AEQPA-----------
GH31-8  R---A-NGSWWNGN-DSYQIDFTNPEAAEWWSAR---LKKLQQN-----------PGIDSFKFDAGETDY--------------SPQPS-----------
GH31-9  R---H-TGWWWNSN-DAYVIDFTNPQATEWWLQR---VKKLQKD-----------FGFDSFKFDAGETDF--------------CPQPA-----------
GH31-10 T---IYQGKYLDQD-VS-YIDYSHENIQKYMEVFGTFLEDFFNHTIDGLVLTDNWPANEEFKMDNDSFPYF--------TKALQDAMSY-----------

GH31-1  KTVCMSAKQYAGLHYNVHNLYGLTEAIVTNFALAEIRG--KRPMVISRSSFSGLGHYAGHWSGDVWSSW---DDLKFSVPTILSFSLYGVPLMGADICGF
GH31-2  -DTI---HHGGWEHRDVHNEYALAHTYATFQGLLQRTPN-KRPFILTRGHFPGSQRYTVVWTGDNAAEW---SHLAVSYPMCLSEALGGMSFCGADVGGF
GH31-3  -DVL---HHGNVPHRDIHNIYGFLQTMATHQGLMQRDNGTKRPFILTRSHFAGSQRYTAMWTGDNTADW---PFLQVSYSECILSNIMGLVFCGADVGGF
GH31-4  -EVQ---HYGGVEHGDIHNIYGLLHVKSTHKGLMARDQNAKRPFVLTRSNFAGSQRYAAMWTGDNSATW---EHFANSISECMNANMLGMVFCGADIGGF
GH31-5  -YYQ------CEKALLNPDEYKTIFTNNLQG---------EVPLFGVNSAIERPRSPSFVVLPEFEFSW---DGLRKIIPTILTYGILGYPFLIPGAVGG
GH31-6  -QFD---HVE--DQEEVPNIFTKKYVETCAQ---------FGDLIEVRSAWRTQNLPVFLRMIDKDSLWTMDDGLESLVTTLIQMNLNGYPLVLPDMVGG
GH31-7  -VFP---YVSRAEQNLLPNLLTTEYIKTCAN---------FGDLIEVRSAFRNQNSSNFVRMLDKDSRWDMQNGLPTLVTTLFQLNLNGYPLVLPDMIGG
GH31-8  -VYH---GV---NQEDVPNILSESYVRTCAK---------FGPLVEVRSGSRTQDLPIFIRMIDKDSNWGDSNGLYTLITTLLQMNINGYTLVLPDMIGG
GH31-9  -VYK---TV---DQELIPNIFSGKYLRTCAT---------FGNLIEVRTGWRTQDLPIFTRMLDKTSNWAYRNGLKSLVTTLIQMNMVGYTMVLPDMIGG
GH31-10 -TIQWNTTANDILHIQKHNSYGGYQYKSLKDYFKS-----KEIFILSATKSISQVEP--MIIENVETSW---TNLRKYLDSVLFDSIIGNHLVSIPVCGD

GH31-1  ---------------NGNSTAPLCNRWMQLGAFYPFSRNHNTDDGIDQDPVAFGP---QVVASSVKAL-TARYYLLPYLYTLFHKAHVTGETVARPLFFE
GH31-2  ---------------FNNPDTELLQRWYQAAVWLPFFRAHAHIDTRRREPYLFPD---DVKNRIRTAL-RLRYAHIPLFYTLFWQHEISGEPVIRPLFYQ
GH31-3  ---------------FHNPETELVQRWYQAGVWLPFYRGHAENNTDRREPYIYST---EVQDVIRNAL-RLRYKHIPVWYTLFYEHTRTGDPVVRPIFYD
GH31-4  ---------------IGDPSDELLQRWYQGAVWTPFFRGHSSRESKRREPYLFSK---DVQDVIRNAL-RLRYQHIPAIYTLFYEHTVTGDPIISPLYYQ
GH31-5  DYAVPETMLVTNGTENVLADPELYIRWLQLATFLPVVRYRHL-------PNSYSQN--NIAGLAKELAKKRHESITPKLKKFARVSLNLGLPIIRPLWML
GH31-6  -----------NSY-RQAPTAELIIRWTQANALMPALQFGFL-------PWDYPSDKFDTTEIVRKYT-NFHEEYGDKIIEAMKASMDKGTPVNPPIWWV
GH31-7  -----------NGYGGNVPTAELIVRWTQANTFMPSMQFSYL-------PWEFNSDEFNTLEIVKKYV-SLHEQYSDVIIKAMERSVSDGSPVNPPIWWI
GH31-8  -----------NGYAGKLPDAELLVRWTQANTFMPAMQFSYL-------PWEITSTKFNVAKIVKKFV-DLHEKYADHIIRAMKNSVEKGSPVNPPIWWI
GH31-9  -----------NGY-GVQPDAELFVRWTQANTFMPAMQFSFL-------PWDFNETKFDVPAIAKSCV-KLHKEYSHIIIDAMKKSVLYGSPVNPPIWWI
GH31-10 -----------TNVFDTSIQMKLCLRWYLIAATMPMFRISAPK------PWRNPD---DLSAKYDQQTAQSALDTRNMLLAYYNGLISSNEPVIRPMFYD

GH31-1  FPQDKNTYGIDTHFLWGSDLLIVPVLKENDVNVTAYIPKGN------------WYNFYTLEG-FYSKGEN---F-TLDAPLDTIPLLVRGGAIVPLQ---
GH31-2  YPNDENVVDIDNEILVGDSVLAAMIAEPGASSVGVYLPGGANEY---------WYDIED--FRLYQGTGN---Y-NIPVSLDKSLAFYRGGSIIPRKD--
GH31-3  YPG---SVDQDYHILLGSNILARPVMEKGARSVTVNFPGSEDNQ---------WYRVDDGSFKIYNGQSS---A-ILPVTILTSPYFYKAGSIITRRD--
GH31-4  YPR---VNDRDTQTMIGADIMSVAVTKPGVKSVEVYFPGGYSDTDPFDKQTDFWYRADQKQFSQYRAGLV---E-QIEVDITSSPVFYRAGSIVFVLE--
GH31-5  DSDDPACHLAVDEFSIGDEIIVAPVLHSGAREREIYLPAGV------------WKDGID--GSLRKGSRW---IHDYRVEEDNVAYFERMP---------
GH31-6  DPTDSFSYTIYDEFLLGEDVLVAPVLVEGATSRNIYLPKGN------------WKDGNS--GETFVGPLN---VTNYDAPIDVLPYFVKTD---------
GH31-7  DPTDPVALACNDEYLVGEDILVAPVLEEKSRSRNVYLPRGR------------WRDGQN--GTVYEGPLS---F-DYPADIDILPYFIKED---------
GH31-8  APKDVQALGCDDEYLVGEEILVAPVIKEGATSRDVYLPAGK------------WVDGNN--GDIYQGPVT---V-DYDAGIDILPFFILSQ---------
GH31-9  DPTDQKALKCDDEFLVGEKILVAPVLTQGATERDVYLPKGK------------WKDGNN--GTYYQGPIT---L-DYTADITTIPFFILEQ---------
GH31-10 FYENTTTFSLYEQYMVGKNMLVVHPLTADRTKMNVYLPPAIDV----------WYEFWG--GDIYKPTKTNPWV-SISVTEADFVAFIPKGSVIPLIDGN

GH31-1  ---------------------KPKNTTTDSRKSKLELL-------IANDERKSASGDLYWDDGDSLNSYKEKQ---YSSLMFELEENTLRSY----ATFI
GH31-2  ---------------------RPRRASPLMKNDPFTLY-------IALDSNKKASGYLYVDDNESFE-YKNQK-YLYLHFSF--EDNTF--N----SVLV
GH31-3  ---------------------EERSSTAEMQDDPFVLY-------VNVDKENTASGNLYVDDYTSFE-YQNNQRYFYANLAY--QSNQI--D----ISIV
GH31-4  ---------------------TPRKSAVEAKDDFYSIY-------VNVDRRNSAKGRLYKDDLYSFN-YQKDN-YFYSEAKWIPGSEKM--Y----MTTI
GH31-5  ----------------------------------------------------------------------------------------------------
GH31-6  ----------------------------------------------------------------------------------------------------
GH31-7  ----------------------------------------------------------------------------------------------------
GH31-8  ----------------------------------------------------------------------------------------------------
GH31-9  ----------------------------------------------------------------------------------------------------
GH31-10 TVNLIVALNFTSSKRTRSFIESSGSATGFMKTDGISITFTANIKSVDISSSSDKKWSLHLGYVKVYQYLNSNKPAYYDVNRSIDQDTQNMPYEPPPTTTT

GH31-1  GSE----IPPNLDRVAVLGIEDSVKEVVVNGAS----HKNFDYNSKNKYLNIDKLDIPLQSPL------VISWK*
GH31-2  DKT-DYPTKEWVERLVILGPPKGIKGAKLTSKSLGTVELDTSYSHEDRVLVVRKPGVSIREKF-----TIKL-Y*
GH31-3  DGN-FSGMTVTLGQVIVHQ--QSDKDEKRYTRTVYTHLADGTPLKS----------INIVEKL-NGNNMVSLKL*
GH31-4  SGTPDQSIRVFYVKVVVHELLESETGELQIKKSMFTKTSGGIPLHK----------IDIAKALREGNGTLELSF*
GH31-5  ---------------------------------------------------------------------DDTRF*
GH31-6  --------------------------------------------------------------------------*
GH31-7  -------------------------------------------------------------------------S*
GH31-8  --------------------------------------------------------------------------*
GH31-9  --------------------------------------------------------------------------*
GH31-10 ESTTSSTTTSTTTASTVTESTTEDTGTTISSTPTMESTTGSTGNTGSSSLISEITTADTGTTSSSTPTRESTTEE
```

B

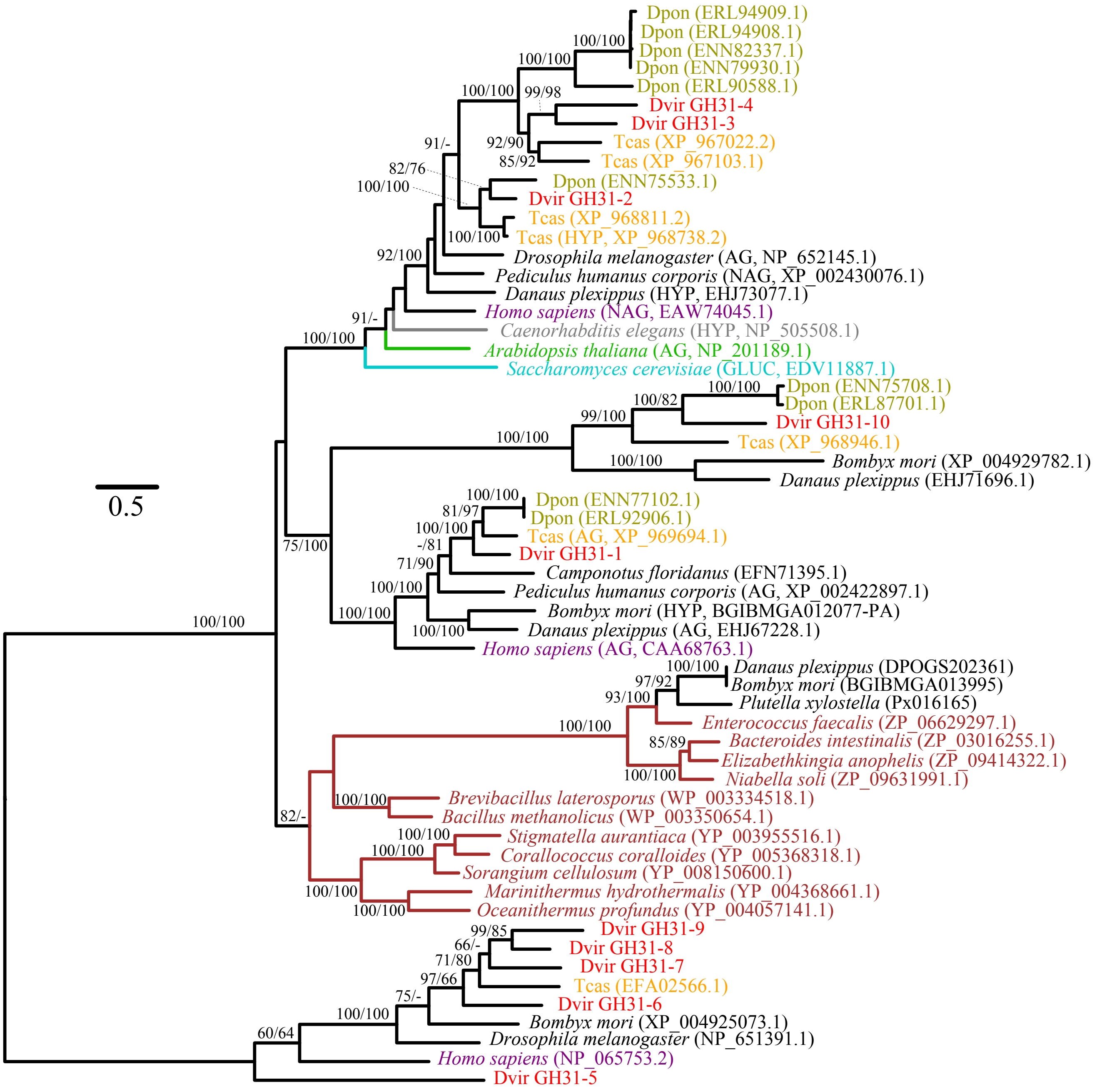

Figure S6. GH31 family gene sequences identified from the *D. v. virgifera* transcriptome (A) and the maximum-likelihood phylogeny of GH31 family proteins (B). In the alignment, the labels for partial sequences are shown in italics. In the phylogeny, labels for the coleopteran species belonging to the superfamily Curculionoidea, *D. v. virgifera*, and other beetle sequences are shown in olive, red, and orange, respectively. Their species abbreviations are found in Table S5. Arthropod, other metazoan, nematode, fungal, plant, and bacterial sequences are indicated by black, purple, grey, cyan, green, and brown, respectively. The accession numbers shown in parenthesis are from NCBI except for: BGIBMGA012077-PA and BGIBMGA013995 from SilkDB (http://www.silkdb.org), DPOGS202361 from MonarchBase (http://monarchbase.umassmed.edu), and Px016165 from Diamondback moth Genome Database (http://59.79.254.1/DBM/). The numbers at internal branches show the bootstrap support values (%) for the maximum-likelihood and neighbor-joining phylogenies in this order. Supporting values are shown only when higher than 60%. The scale bar represents the number of amino acid substitutions per site.
