# Supplementary material for: Molecular Evolution of Glycoside Hydrolase Genes in the Western Corn Rootworm (Diabrotica virgifera virgifera)
Source: PLoS One. 2014 Apr 9;9(4):e94052. doi: 10.1371/journal.pone.0094052 (PMC3981738; doi:10.1371/journal.pone.0094052)
Supplement: Figure S7 — GH27 family gene sequences identified from the D. v. virgifera transcriptome (A) and the maximum-likelihood phylogeny including representative GH27 family proteins (B). Labels for the coleopteran species belonging to the superfamily Curculionoidea, D. v. virgifera, and other beetle sequences are shown in olive, red, and orange, respectively. Their species abbreviations are found in Table S5. Arthropod, other metazoan, nematode, fungal, plant, and bacterial sequences are indicated by black, purple, grey, cyan, green, and brown, respectively. Bacterial sequences were used as outgroups. The numbers at internal branches show the bootstrap support values (%) for the maximum-likelihood and neighbor-joining phylogenies in this order. Supporting values are shown only when higher than 60%. The scale bar represents the number of amino acid substitutions per site. (PDF) [file pone.0094052.s007.pdf]

A

```
GH27-1 MVS---RLVLALVGLISTVNGLDNGLARTPPMGWMDWQRFRCLTNCTLYPDECISEKLFRDMADRMAADGYLAAGYEYIMIDDCWSSKER
GH27-2 MYKIWFVLAVVVFYLGIDVTPLENGLARTPPMGWLAWERFRCNTDCKNDPENCISENLFRTMADILVNEGYASVGYEYINVDDCWLEKDR

GH27-1 DSKGRLVPDPDRFPSGIKNLSDYIHSKGLKFGIYADYGTLTCAGYPGSKEYLKIDADRFAEWEVDYLKFDGCNSDWIFIDKGYIEMGKHL
GH27-2 SVYGELVPDRVRFPRGMKSLADYVHSKGLKFGIYEDYGNYTCAGYPGVLGSLQRDAETFASWDVDYVKLDGCYAHPRDMDRGYPEFGFHL

GH27-1 NATGRPIVYSCSWPAYQEPNKMQSNYTALAETCNLWRNWDDIDDSWESVTSIIEWFSDNQDRIGPFSAPGHWNDPDMLVIGNFGLSFEQS
GH27-2 NRTGRAMIYSCSWPVYQIYAGMSPNFSAIIEHCNMWRNFDDIQDSWTSVESIIDYYGNNQDVLIANAGPGHWNDPDMLIIGNFGLSYEQS

GH27-1 KGQMSVWSVMAAPLIMSVDLRTIEPKFRAILLNKDAIAVNQDPLGEMGRLVLKKNNIYIWTKKLTAKADGRQPHAIAVLSQRTDGYKYRM
GH27-2 KTQMAIWAILAAPLLMSVDLRTIRPEYKAILQNRKIIAVDQDPLGIQGRRIYKHKGIEIWSRPITPLYQSYFSYAIAFVNRRTDGTPSDV

GH27-1 EFTLKDLNITGPNGFLIKDIFDEDKSVASIADDEPFVLRMAPTGGTLLVATPKK-------------------------------------
GH27-2 AVTLKELGLTSPTGYRVEDLY-EDVDYGVLSPQTKIKVKVNPSGVVILRADVQADFNRRIPFFTTQRPFSSSPLNQVFRVRENGFKPFFT

GH27-1 ----------------------
GH27-2 TQRPFSSSPLNQVFRVRENGFK
```

B

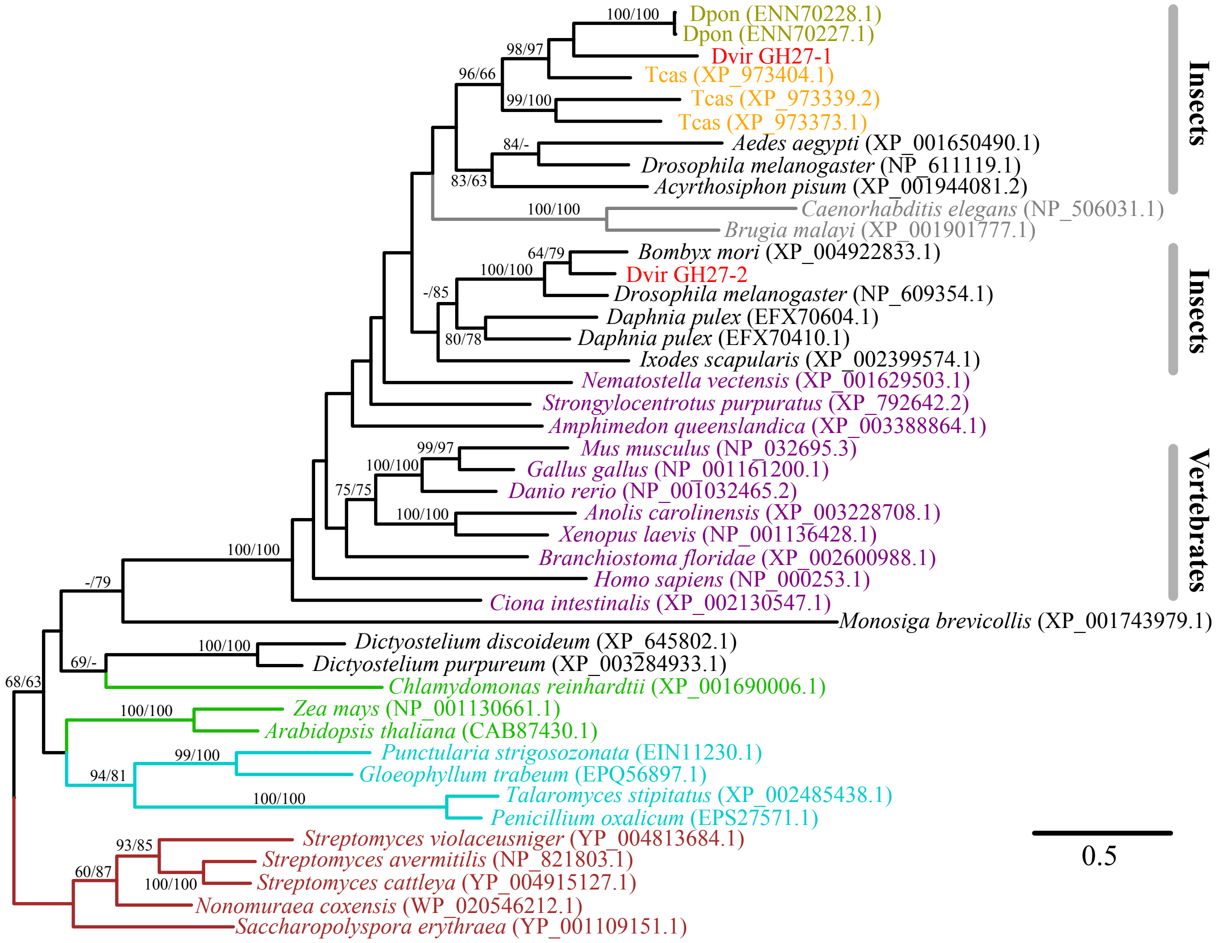

Figure S7. GH27 family gene sequences identified from the *D. v. virgifera* transcriptome (A) and the maximum-likelihood phylogeny including representative GH27 family proteins (B). Labels for the coleopteran species belonging to the superfamily Curculionoidea, *D. v. virgifera*, and other beetle sequences are shown in olive, red, and orange, respectively. Their species abbreviations are found in Table S5. Arthropod, other metazoan, nematode, fungal, plant, and bacterial sequences are indicated by black, purple, grey, cyan, green, and brown, respectively. Bacterial sequences were used as outgroups. The numbers at internal branches show the bootstrap support values (%) for the maximum-likelihood and neighbor-joining phylogenies in this order. Supporting values are shown only when higher than 60%. The scale bar represents the number of amino acid substitutions per site.
