# Supplementary material for: Molecular Evolution of Glycoside Hydrolase Genes in the Western Corn Rootworm (Diabrotica virgifera virgifera)
Source: PLoS One. 2014 Apr 9;9(4):e94052. doi: 10.1371/journal.pone.0094052 (PMC3981738; doi:10.1371/journal.pone.0094052)
Supplement: Figure S8 — The distribution of E-values obtained from blastx similarity search against the UniProt protein database using the assemblies generated by three programs using the D. v. virgifera egg samples. The numbers of contigs are 18,173 in Mira (blue), 11,035 in Trinity (red), and 9843 in Velvet/Oasis (green). E-values are shown as −log10 (E-value) except for E-value = 0. Note that there is no significant difference between Trinity and Mira (t-test P>0.5 for both Evalue ≤10−100 and for all E-values). (PDF) [file pone.0094052.s008.pdf]

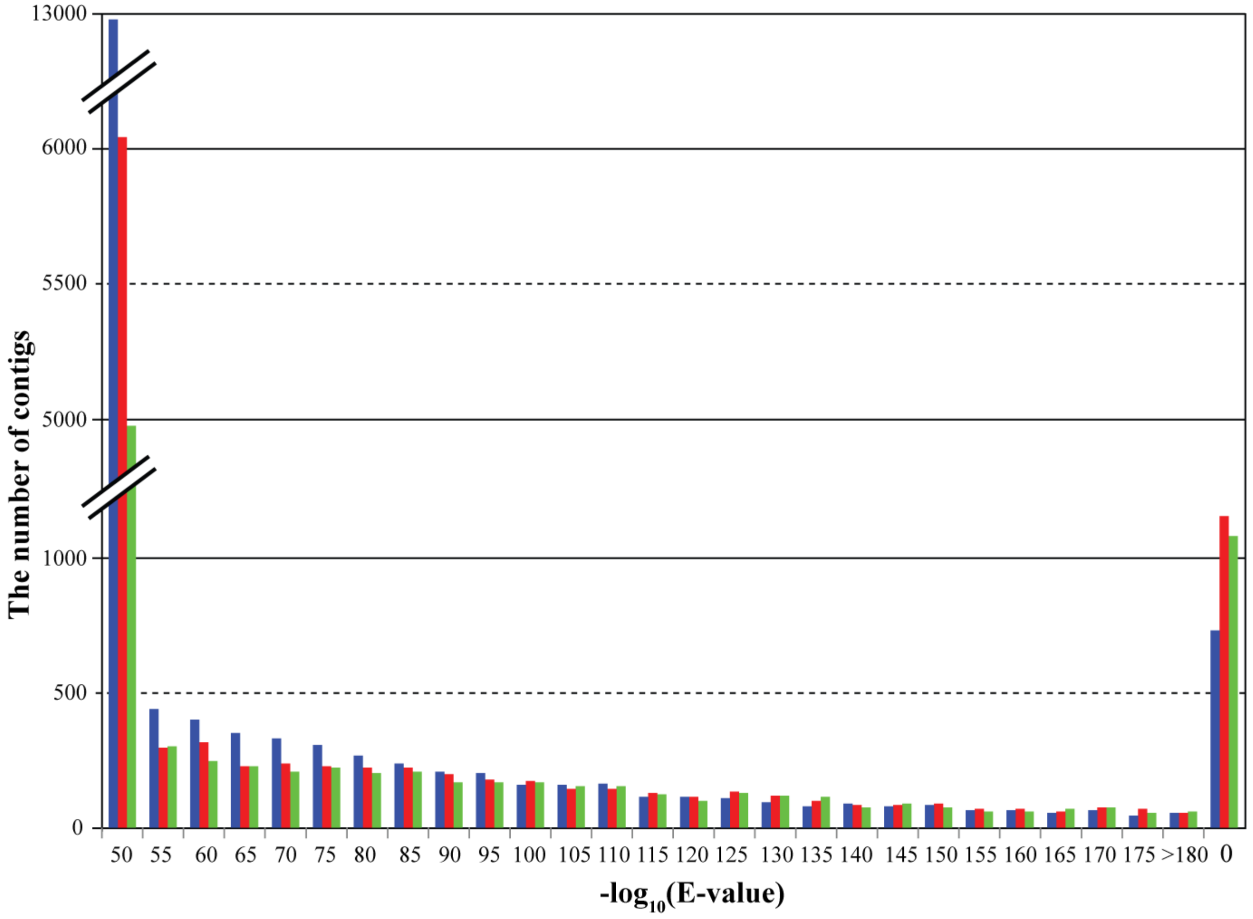

Figure S8 The distribution of E-values obtained from blastx similarity search against the UniProt protein database using the assemblies generated by three programs using the *D*. *v*. *virgifera* egg samples. The numbers of contigs are 18,173 in Mira (blue), 11,035 in Trinity (red), and 9843 in Velvet/Oasis (green). E-values are shown as $-\log_{10}$(E-value) except for E-value=0. Note that there is no significant difference between Trinity and Mira (*t*-test $P > 0.5$ for both E-value $\leq 10^{-100}$ and for all E-values).
