# Supplementary material for: Molecular Evolution of Glycoside Hydrolase Genes in the Western Corn Rootworm (Diabrotica virgifera virgifera)
Source: PLoS One. 2014 Apr 9;9(4):e94052. doi: 10.1371/journal.pone.0094052 (PMC3981738; doi:10.1371/journal.pone.0094052)
Supplement: Table S1 — Summary statistics for D. v. virgifera transcriptome sequencing and assembly. (PDF) [file pone.0094052.s009.pdf]

Table S1. Summary statistics for *D. v. virgifera* transcriptome sequencing and assembly.

**egg 0-10 day total RNA (Illumina, paired-end)**	
Total number of paired-end reads before filtering (length)	38,657,737 (2,899,330,275 bp)
Number of paired-end reads that entered assembly after > Q20 filtering (length)	15,162,017 (1,137,151,275 bp)
Assembly program used	Trinity (2013-02-25)
Total number of contigs	72,638
Average contig length (range)	825 bp (201 – 13,911 bp)
N50 length	1,357 bp
Assembly program used	Velvet/Oasis (ver. 1.2.03)
Total number of contigs	56,135
Average contig length (range)	583 bp (100 – 10,434 bp)
N50 length	850 bp
Assembly program used	Mira (ver. 3.4.0)
Total number of contigs	69,815
Average contig length (range)	520 bp (100 – 13,526 bp)
N50 length	850 bp
**Third larval midgut RNA (Illumina, paired-end)**	
Total number of paired-end reads before filtering (length)	76,202,715 (5,715,203,625 bp)
Number of paired-end reads that entered assembly after > Q20 filtering (length)	44,852,488 (3,363,936,600 bp)
Assembly program used	Trinity (2013-02-25)
Total number of contigs	72,325
Average contig length (range)	859 bp (201 – 17,831 bp)
N50 length	1,435 bp
Assembly program used	Velvet/Oasis (ver. 1.2.03)
Total number of contigs	96,215
Average contig length (range)	635 bp (100 – 17,673 bp)
N50 length	1,180 bp
**Third larval midgut RNA (Roche 454)**	
Total number of reads before filtering (length)	664,431 (361,187,777 bp)
Number of reads that entered assembly after filtering (removing the adapters and > Q20)	415,742 (210,423,467 bp)
Assembly program used	Trinity (2013-02-25)
Total number of contigs	37,181
Average contig length (range)	614 bp (201 – 5,044 bp)
N50 length	743 bp

Assembly program used	Newbler (ver. 2.5)
Total number of contigs	45,994
Average contig length (range)	535 bp (51 – 4,098)
N50 length	595 bp
Assembly program used	Velvet/Oasis (ver. 1.2.03)
Total number of contigs	165,361
Average contig length (range)	322 bp (100 – 5,807 bp)
N50 length	481 bp
Assembly program used	Mira (ver. 3.4.0)
Total number of contigs	57,923
Average contig length (range)	762 bp (100 – 3,032 bp)
N50 length	853 bp
**Neonates RNA (Illumina Hi-seq, paired-end)**	
Total number of paired-end reads before filtering (length)	1,347,291,731 (136,076,464,831 bp)
Number of paired-end reads that entered assembly after > Q30 filtering (length)	721,697,288 (72,891,426,088 bp)
Assembly program used	Trinity
Total number of contigs	155,787
Average contig length (range)	937 bp (201 – 25,737 bp)
N50 length	1,817 bp
