# Supplementary material for: Molecular Evolution of Glycoside Hydrolase Genes in the Western Corn Rootworm (Diabrotica virgifera virgifera)
Source: PLoS One. 2014 Apr 9;9(4):e94052. doi: 10.1371/journal.pone.0094052 (PMC3981738; doi:10.1371/journal.pone.0094052)
Supplement: Table S2 — Summary of D . v . virgifera transcriptome sequencing and assemblies. (PDF) [file pone.0094052.s010.pdf]

**Table S2. Summary of *D. v. virgifera* transcriptome sequencing and assemblies.**

| | | Egg | Larval midgut | Larval midgut | Neonates |
|---|---|---|---|---|---|
| Sequencing platform | | Illumina Genome Analyzer II | Illumina Genome Analyzer II | 454 Titanium | Illumina HiSeq2000 |
| Read length | | 75 bp | 75 bp | NA | 101 bp |
| Total reads[a] | | 15.1 M | 44.8 M | 415,742 | 721 M |
| Total number of contigs (average length) | Trinity | 72,638 (825 bp) | 72,325 (859 bp) | 37,181 (614 bp) | 155,787 (914 bp) |
| | Newbler | NA | NA | 45,994 (535 bp) | NA |
| | Velvet/Oasis | 56,135 (520 bp) | 96,215 (635 bp) | 165,361 (322 bp) | NA |
| | Mira | 69,815 (520 bp) | NA | 57,923 (762 bp) | NA |

[a]Numbers of reads used for assembly after filtering. M: million paired-end.
