# Supplementary material for: Molecular Evolution of Glycoside Hydrolase Genes in the Western Corn Rootworm (Diabrotica virgifera virgifera)
Source: PLoS One. 2014 Apr 9;9(4):e94052. doi: 10.1371/journal.pone.0094052 (PMC3981738; doi:10.1371/journal.pone.0094052)
Supplement: Table S3 — Summary statistics for hybrid and pooled-data assembly of D. v. virgifera transcriptome. (PDF) [file pone.0094052.s011.pdf]

Table S3. Summary statistics for hybrid and pooled-data assembly of *D. v. virgifera* transcriptome.

**Hybrid (454 + Illumina) assembly of third larval midgut**	
Assembly program used	Trinity (2013-02-25)
Total number of contigs	81,858
Average contig length (range)	862 bp (201 – 17,831 bp)
N50 length	1,396 bp
Assembly program used	Velvet/Oasis (ver. 1.2.03)
Total number of contigs	133,276
Average contig length (range)	425 bp (100 - 16,733 bp)
N50 length	675 bp
**Hybrid (454 + Illumina) assembly of egg and third larval midgut**	
Assembly program used	Trinity (2012-03-17)
Total number of contigs	101,915
Average contig length (range)	662 bp (201 – 13,611 bp)
N50 length	1,006 bp
**The pooled read dataset (egg + neonates + third larval midgut)**	
Assembly program used	Trinity (2013-02-25)
Total number of contigs	163,871
Average contig length (range)	914 bp (201 – 31,064 bp)
N50 length	1,396 bp
