# Supplementary material for: Molecular Evolution of Glycoside Hydrolase Genes in the Western Corn Rootworm (Diabrotica virgifera virgifera)
Source: PLoS One. 2014 Apr 9;9(4):e94052. doi: 10.1371/journal.pone.0094052 (PMC3981738; doi:10.1371/journal.pone.0094052)
Supplement: Figure S2 — The maximum-likelihood phylogeny of GH45 family proteins. Labels for the coleopteran species belonging to the superfamily Curculionoidea are olive-colored and all other coleopteran sequences colored in black belong to the superfamily Chrysomeloidea. D. v. virgifera sequences are shown in red. Their species name abbreviations are found in Table S5. Other sequences are colored as follows: mollusks (purple), Cryptopygus antarcticus (Collembola, black), Hypsibius dujardini (Tardigrada, black), protists (dark green), plant-parasitic nematodes (grey), fungi (cyan), and bacteria (brown). The scale bar represents the number of amino acid substitutions per site. (PDF) [file pone.0094052.s002.pdf]

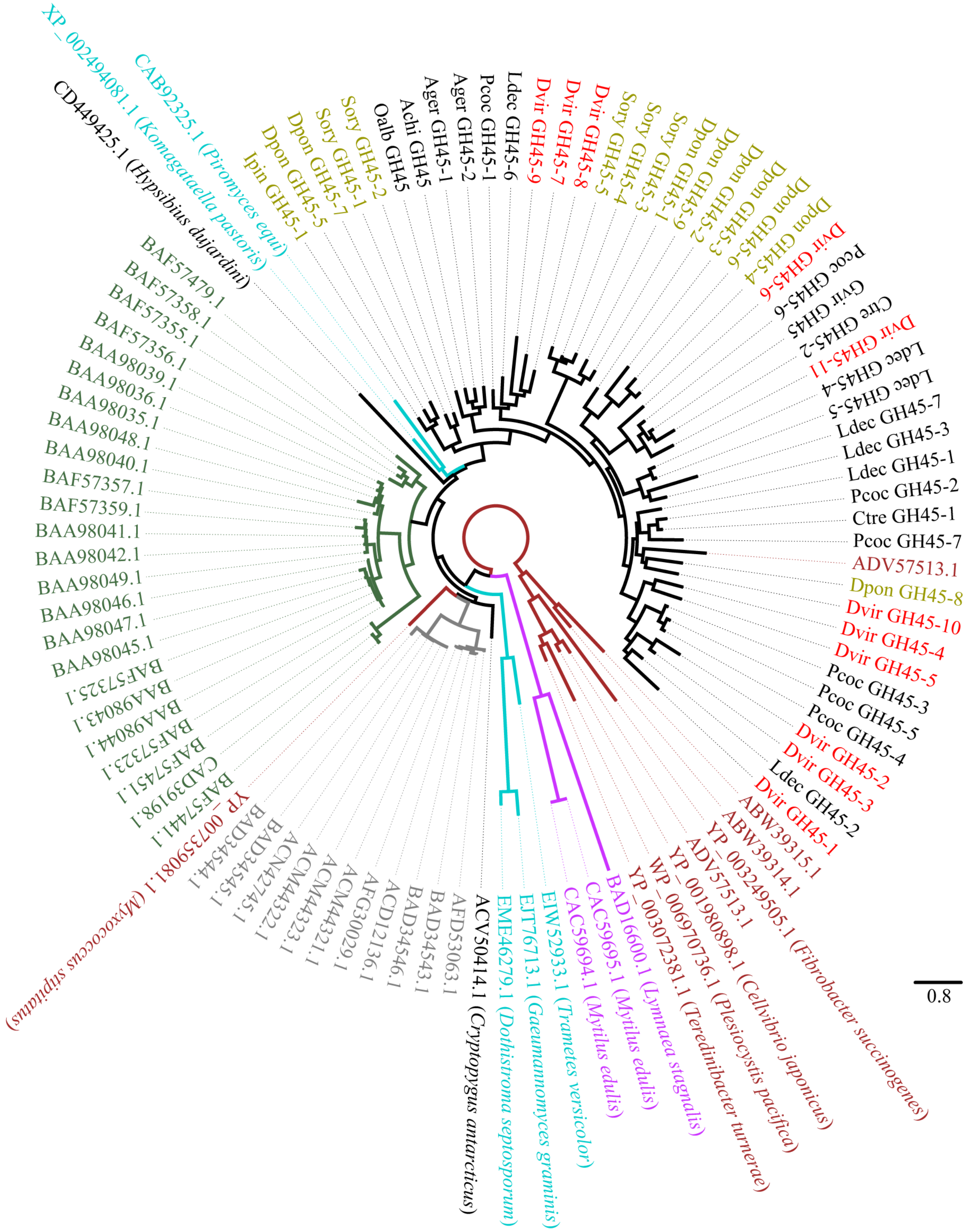

Figure S2. The maximum-likelihood phylogeny of GH45 family proteins. Labels for the coleopteran species belonging to the superfamily Curculionoidea are olive-colored and all other coleopteran sequences colored in black belong to the superfamily Chrysomeloidea. *D. v. virgifera* sequences are shown in red. Their species name abbreviations are found in Table S5. Other sequences are colored as follows: mollusks (purple), *Cryptopygus antarcticus* (Collembola, black), *Hypsibius dujardini* (Tardigrada, black), protists (dark green), plant-parasitic nematodes (grey), fungi (cyan), and bacteria (brown). The scale bar represents the number of amino acid substitutions per site.
