# Supplementary material for: Molecular Evolution of Glycoside Hydrolase Genes in the Western Corn Rootworm (Diabrotica virgifera virgifera)
Source: PLoS One. 2014 Apr 9;9(4):e94052. doi: 10.1371/journal.pone.0094052 (PMC3981738; doi:10.1371/journal.pone.0094052)
Supplement: Table S5 — Coleopteran GH family gene sequences used in this study. (PDF) [file pone.0094052.s013.pdf]

Table S5. Coleopteran species and GH protein sequences used in this study.

Species	GH9	GH5	GH45	GH48	GH28	GH16	GH1	GH31	GH27	GH11
**[Chrysomeloidea]**										
Chrysomela tremulae	None	None	ADU33285.1 Ctre_GH45-1<br>ADU33286.1 Ctre_GH45-2	ADU33283.1 Ctre_GH48_1<br>ADU33284.1 Ctre_GH48_2	ACP18831.1 Ctre_GH28-1<br>ADU33275.1 Ctre_GH28-2<br>ADU33276.1 Ctre_GH28-3<br>ADU33277.1 Ctre_GH28-4<br>ADU33278.1 Ctre_GH28-5<br>ADU33279.1 Ctre_GH28-6<br>ADU33280.1 Ctre_GH28-7<br>ADU33281.1 Ctre_GH28-8<br>ADU33282.1 Ctre_GH28-9	-	-	-	-	-
Gastrophysa viridula	None	ADU33333.1 Gvir_GH5	ADU33334.1 Gvir_GH45	ADU33335.1 Gvir_GH48_1<br>ADU33336.1 Gvir_GH48_2<br>ADU33337.1 Gvir_GH48_3	ADU33338.1 Gvir_GH28-1<br>ADU33339.1 Gvir_GH28-2<br>ADU33340.1 Gvir_GH28-3<br>ADU33341.1 Gvir_GH28-4<br>ADU33342.1 Gvir_GH28-5<br>ADU33343.1 Gvir_GH28-6<br>ADU33344.1 Gvir_GH28-7	-	-	-	-	-
Leptinotarsa decemlineata	None	None	ADU33345.1 Ldec_GH45-1<br>ADU33346.1 Ldec_GH45-2<br>ADU33347.1 Ldec_GH45-3<br>ADU33348.1 Ldec_GH45-4<br>ADU33349.1 Ldec_GH45-5<br>ADU33350.1 Ldec_GH45-6<br>ADU33351.1 Ldec_GH45-7	ADU33352.1 Ldec_GH48_1<br>ADU33353.1 Ldec_GH48_2<br>ADU33354.1 Ldec_GH48_3	ADU33355.1 Ldec_GH28-1<br>ADU33356.1 Ldec_GH28-2<br>ADU33357.1 Ldec_GH28-3<br>ADU33358.1 Ldec_GH28-4<br>ADU33359.1 Ldec_GH28-5<br>ADU33360.1 Ldec_GH28-6<br>ADU33361.1 Ldec_GH28-7<br>ADU33362.1 Ldec_GH28-8<br>ADU33363.1 Ldec_GH28-9<br>ADU33364.1 Ldec_GH28-10<br>AEX93414.1 Ldec_GH28-11**	-	-	-	-	-
Phaedon cochleariae	-	-	CCJ09450.1 Pcoc_GH45-1<br>CCJ09451.1 Pcoc_GH45-2<br>CCJ09452.1 Pcoc_GH45-3<br>CCJ09453.1 Pcoc_GH45-4<br>CCJ09454.1 Pcoc_GH45-5<br>CCJ09455.1 Pcoc_GH45-6<br>CCJ09456.1 Pcoc_GH45-7	-	CCJ09441.1 Pcoc_GH28-1<br>CCJ09442.1 Pcoc_GH28-2<br>CCJ09443.1 Pcoc_GH28-3<br>CCJ09444.1 Pcoc_GH28-4<br>CCJ09445.1 Pcoc_GH28-5<br>CCJ09446.1 Pcoc_GH28-6<br>CCJ09447.1 Pcoc_GH28-7<br>CCJ09448.1 Pcoc_GH28-8<br>CCJ09449.1 Pcoc_GH28-9	-	-	-	-	AGK45631.1<br>AGK45632.1
Gastrophysa atrocyanea	-	-	-	BAE94320.1 Gatr_GH48-1<br>BAE94321.1 Gatr_GH48-2	-	-	-	-	-	-
Callosobruchus maculatus	None	ADU33271.1 Cmac_GH5-1<br>ADU33272.1 Cmac_GH5-2<br>ADU33273.1 Cmac_GH5-3<br>ADU33274.1 Cmac_GH5-4	None	None	ADU33264.1 Cmac_GH28-1<br>ADU33265.1 Cmac_GH28-2<br>ADU33266.1 Cmac_GH28-3<br>ADU33267.1 Cmac_GH28-4<br>ADU33268.1 Cmac_GH28-5<br>ADU33269.1 Cmac_GH28-6<br>ADU33270.1 Cmac_GH28-7	-	-	-	-	-
Apriona germari	-	AAX18655.1 Ager_GH5	AAU44973.1 Ager_GH45-1<br>AAR22385.1 Ager_GH45-2	-	-	-	-	-	-	-
Psacothea hilaris	-	BAB86867.1 Phil_GH5	-	-	-	-	-	-	-	-
Anoplophora chinensis	-	AFN89566.1 Achi_GH5	AFN89565.1 Achi_GH45	-	-	-	-	-	-	-
Oncideres albomarginata chamela	-	ADI24131.1 Oalb_GH5	ADI24132.1 Oalb_GH45	-	-	-	-	-	-	-

**[Curculionoidea]**										
Dendroctonus ponderosae	None	None	ADU33287.1 Dpon_GH45-1<br>ADU33288.1 Dpon_GH45-2<br>ADU33289.1 Dpon_GH45-3<br>ADU33290.1 Dpon_GH45-4<br>ADU33291.1 Dpon_GH45-5<br>ADU33292.1 Dpon_GH45-6<br>ADU33293.1 Dpon_GH45-7<br>ADU33294.1 Dpon_GH45-8<br>ADU33295.1 Dpon_GH45-9	ADU33296.1 Dpon_GH48_1<br>ADU33297.1 Dpon_GH48_2<br>ADU33298.1 Dpon_GH48_3<br>ADU33299.1 Dpon_GH48_4<br>ADU33300.1 Dpon_GH48_5<br>ADU33301.1 Dpon_GH48_6	ADU33302.1 Dpon_GH28-1<br>ADU33303.1 Dpon_GH28-2<br>ADU33304.1 Dpon_GH28-3<br>ADU33305.1 Dpon_GH28-4<br>ADU33306.1 Dpon_GH28-5<br>ADU33307.1 Dpon_GH28-6<br>ADU33308.1 Dpon_GH28-7<br>ADU33309.1 Dpon_GH28-8<br>ADU33310.1 Dpon_GH28-9<br>ADU33311.1 Dpon_GH28-10<br>ADU33312.1 Dpon_GH28-11<br>ADU33313.1 Dpon_GH28-12<br>ADU33314.1 Dpon_GH28-13<br>ADU33315.1 Dpon_GH28-14<br>ADU33316.1 Dpon_GH28-15<br>ADU33317.1 Dpon_GH28-16<br>ADU33318.1 Dpon_GH28-17<br>ADU33319.1 Dpon_GH28-18<br>ADU33320.1 Dpon_GH28-19	ENN78831.1<br>ENN78830.1<br>ENN83076.1<br>ENN83093.1<br>ENN72429.1	AEE63189.1<br>AEE62454.1<br>AEE61591.1<br>AEE63265.1<br>ERL86564.1<br>AEE62108.1<br>AEE61793.1<br>AEE62242.1<br>ERL84361.1<br>ERL83310.1<br>ERL83693.1<br>ERL84272.1<br>ERL91732.1<br>ENN80860.1<br>AEE62089.1<br>AEE62876.1<br>ENN74637.1<br>ENN73453.1<br>AEE61395.1	ERL94909.1<br>ERL94908.1<br>ENN82337.1<br>ENN79930.1<br>ERL90588.1<br>ENN75533.1<br>ENN75708.1<br>ERL87701.1<br>ENN77102.1<br>ERL92906.1	XP_970010.1<br>ENN70227.1	-
Ips pini	-	None	CB408544 Ipin_GH45_1	-	-	-	-	-	-	-
Hypothenemus hampei	-	ACU52526.1 Hham_GH5-1<br>ACU52527.1 Hham_GH5-2	-	-	-	-	-	-	-	-
Sitophilus oryzae	None	None	ADU33246.1 Sory_GH45-1<br>ADU33247.1 Sory_GH45-2<br>ADU33248.1 Sory_GH45-3<br>ADU33249.1 Sory_GH45-4<br>ADU33250.1 Sory_GH45-5	ADU33251.1 Sory_GH48_1<br>ADU33252.1 Sory_GH48_2	ADU33253.1 Sory_GH28-1<br>ADU33254.1 Sory_GH28-2<br>ADU33255.1 Sory_GH28-3<br>ADU33256.1 Sory_GH28-4<br>ADU33257.1 Sory_GH28-5<br>ADU33258.1 Sory_GH28-6	-		-		-
Otiorhynchus sulcatus	-	-	-	CAH25542.1 Osul_GH48	-	-	-	-	-	-
**[Tenebrionoidea]**										
Tribolium castaneum	XP_001810693.1	None	None	None	None	XP_970010.1	XP_975666.<br>XP_975665.<br>XP_972437.<br>XP_972082.<br>XP_972032.<br>XP_972134.<br>XP_968318.<br>XP_970224.1<br>XP_972386.1<br>EFA06171.1<br>XP_972182.1<br>XP_972285.1<br>XP_966332.1<br>XP_972231.1<br>XP_972342.1	XP_967022.2<br>XP_967103.1<br>XP_968811.2<br>XP_968738.2<br>XP_968946.1<br>XP_969694.1<br>EFA02566.1	XP_973404.1<br>XP_973339.2<br>XP_973373.1	-
**[Diptera]**										
Drosophila melanogaster	None	None	None*	None	None*	-	NP_648918.	NP_652145.1<br>NP_651391.1	NP_611119.1<br>NP_609354.1	-

NOTE:
For each sequence, its accession number (NCBI) and the ID used in this study is listed. For GH families that were not included in our study '-' is shown.

*In Calderón-Cortés *et al.* [12], *Drosophila melanogaster* is shown to have two GH45 (EC068056 and CO334668) and one GH28 (CO335003). The nucleotide sequences of these two GH45s are 100% identical, and there are only three nucleotide differences between these *D. melanogaster* GH45s and *L. decemlineata* GH45-7 (ADU33351.1) (100% identical in amino acid sequences). The *D. melanogaster* GH28 and *L. decemlineata* GH28-9 (ADU33363.1) are also 100% identical at the nucleotide level. Furtheremore, BLAST similarity search against the *D. melanogaster* genome (ver. 5.51; http://flybase.org) failed to find any similar sequences. Therefore, we concluded that these are likely misidentifications. GH45 and GH28 are absent in *D. melanogaster*.

**Pauchet *et al.* [22] showed ten GH28 genes (ADU33355.1-ADU33364.1) in *L. decemlineata*. However, we found one more GH28 gene (AEX93414.1, Ldec_GH28-11) in the NCBI NR database.
