# Supplementary material for: Molecular Evolution of Glycoside Hydrolase Genes in the Western Corn Rootworm (Diabrotica virgifera virgifera)
Source: PLoS One. 2014 Apr 9;9(4):e94052. doi: 10.1371/journal.pone.0094052 (PMC3981738; doi:10.1371/journal.pone.0094052)
Supplement: Table S4 — Expression analysis of the D . v . virgifera GH family genes identified in this study. (PDF) [file pone.0094052.s012.pdf]

**Table S4. Expression analysis of *D. v. virgifera* GH family genes identified in this study.[a]**

| Genes | Length (AA)[b] | Egg (38,657,737)[c] | | Neonate (21,864,095)[c] | | 3rd-instar midgut (40,096,158)[c] | |
|---|---|---|---|---|---|---|---|
| | | Read[d] | RPKM | Read[d] | RPKM | Read[d] | RPKM |
| GH45-1 | 720 (239) | 1 | 0.04 | 4053 | 258.59 | 888 | 30.89 |
| GH45-2 | 741 (246) | 2 | 0.07 | 343 | 21.26 | 485 | 16.39 |
| GH45-3 | 729 (242) | 3 | 0.11 | 9 | 0.57 | 142 | 4.88 |
| GH45-4 | 741 (246) | 5 | 0.18 | 2135 | 132.30 | 26621 | 899.63 |
| GH45-5 | 729 (242) | 0 | 0 | 12 | 0.76 | 79 | 2.71 |
| GH45-6* | 615 (204) | 1 | 0.04 | 15 | 1.12 | 538 | 21.92 |
| GH45-7 | 720 (239) | 2 | 0.07 | 61252 | 3907.24 | 228852 | 7960.36 |
| GH45-8 | 717 (238) | 6 | 0.19 | 14 | 0.89 | 86 | 3.00 |
| GH45-9 | 720 (239) | 0 | 0 | 796 | 50.78 | 2473 | 86.02 |
| GH45-10 | 717 (238) | 4 | 0.14 | 11641 | 745.75 | 67084 | 2343.23 |
| GH45-11 | 735 (244) | 2 | 0.07 | 31 | 1.94 | 656 | 22.35 |
| | | | | | | | |
| GH48-1 | 1926 (641) | 1 | 0.01 | 3 | 0.07 | 26 | 0.33 |
| GH48-2* | 374 (124) | 1 | 0.06 | 0 | 0 | 2 | 0.13 |
| GH48-3 | 1926 (641) | 2 | 0.02 | 302 | 7.18 | 965 | 12.51 |
| | | | | | | | |
| GH28-1 | 1098 (365) | 0 | 0 | 171 | 7.14 | 319 | 7.27 |
| GH28-2 | 1098 (365) | 0 | 0 | 384 | 16.04 | 1098 | 25.01 |
| GH28-3 | 1116 (371) | 0 | 0 | 36 | 1.48 | 175 | 3.92 |
| GH28-4* | 1097 (365) | 21 | 0.50 | 3 | 0.13 | 221 | 5.03 |
| GH28-5 | 1062 (353) | 0 | 0 | 55 | 2.38 | 294 | 6.92 |
| GH28-6 | 1092 (363) | 2 | 0.05 | 707 | 29.69 | 11750 | 269.09 |
| GH28-7 | 1071 (356) | 1 | 0.02 | 18 | 0.77 | 192 | 4.48 |
| GH28-8* | 855 (284) | 5 | 0.15 | 1 | 0.05 | 3 | 0.08 |
| GH28-9 | 1101 (366) | 6 | 0.14 | 184 | 7.66 | 1105 | 25.10 |
| GH28-10* | 813 (270) | 22 | 0.70 | 1 | 0.06 | 4 | 0.12 |
| GH28-11 | 1062 (353) | 4 | 0.09 | 2 | 0.09 | 8 | 0.18 |
| GH28-12 | 1068 (355) | 1 | 0.02 | 3 | 0.13 | 18 | 0.42 |
| GH28-13 | 1104 (367) | 1 | 0.02 | 28 | 1.16 | 15 | 0.33 |
| GH28-14* | 366 (122) | 0 | 0 | 3 | 0.37 | 3 | 0.20 |
| | | | | | | | |
| GH16-1 | 1125 (374) | 3 | 0.07 | 36861 | 1502.60 | 39623 | 880.75 |
| GH16-2 | 1116 (371) | 668 | 15.53 | 497 | 20.42 | 1297 | 29.06 |
| GH16-3 | 1119 (372) | 614 | 14.23 | 501 | 20.53 | 1425 | 31.85 |
| GH16-4 | 1113 (370) | 573 | 13.35 | 262 | 10.80 | 31 | 0.70 |
| GH16-5 | 1149 (382) | 69 | 1.56 | 5 | 0.20 | 331 | 7.20 |
| GH16-6* | 696 (232) | 0 | 0 | 0 | 0 | 1071 | 38.38 |
| GH16-7* | 448 (149) | 1 | 0.06 | 6 | 0.61 | 2 | 0.11 |
| GH16-8* | 925 (307) | 69 | 1.93 | 506 | 25.02 | 31 | 0.84 |
| GH16-9* | 842 (280) | 10 | 0.31 | 4 | 0.22 | 13 | 0.39 |
| | | | | | | | |
| GNBP-1 | 1353 (450) | 40 | 0.76 | 173 | 5.85 | 34 | 0.63 |
| GNBP-2 | 1500 (499) | 301 | 5.19 | 53 | 1.62 | 48 | 0.80 |
| GNBP-3 | 1671 (556) | 408 | 6.32 | 0 | 0 | 0 | 0 |
| | | | | | | | |
| GH5* | 317 (105) | 6 | 0.49 | 0 | 0 | 0 | 0 |

| | | | | | | | |
|---|---|---|---|---|---|---|---|
| GH1-1 | 1491 (496) | 1 | 0.02 | 27 | 0.83 | 5 | 0.08 |
| GH1-2 | 1458 (485) | 1 | 0.02 | 558 | 17.50 | 2637 | 45.11 |
| GH1-3 | 1497 (498) | 1 | 0.02 | 158 | 4.83 | 533 | 8.88 |
| GH1-4 | 1494 (497) | 2 | 0.03 | 1561 | 47.79 | 303 | 5.06 |
| GH1-5 | 1509 (502) | 5 | 0.09 | 2787 | 84.47 | 7932 | 131.10 |
| GH1-6 | 1458 (485) | 0 | 0 | 56 | 1.76 | 1117 | 19.11 |
| GH1-7 | 1542 (513) | 4 | 0.07 | 193 | 5.72 | 4099 | 66.30 |
| GH1-8 | 1584 (527) | 5 | 0.08 | 501 | 14.47 | 76 | 1.20 |
| GH1-9 | 1686 (561) | 3 | 0.05 | 2016 | 54.69 | 53 | 0.78 |
| GH1-10 | 1461 (486) | 3 | 0.05 | 64 | 2.00 | 429 | 7.32 |
| GH1-11 | 2073 (690) | 95 | 1.19 | 3 | 0.07 | 0 | 0 |
| GH1-12 | 1551 (516) | 357 | 5.95 | 1 | 0.03 | 11 | 0.18 |
| GH1-13 | 1464 (487) | 29 | 0.51 | 202 | 6.31 | 1232 | 20.99 |
| GH1-14 | 1452 (483) | 1 | 0.02 | 338 | 10.65 | 1735 | 29.80 |
| GH1-15 | 1494 (497) | 2 | 0.03 | 321 | 9.83 | 4033 | 67.32 |
| GH1-16 | 1479 (492) | 0 | 0 | 314 | 9.71 | 2494 | 42.06 |
| GH1-17 | 1524 (507) | 2 | 0.03 | 2137 | 64.13 | 6239 | 102.10 |
| GH1-18 | 1491 (496) | 5 | 0.09 | 5590 | 171.48 | 15378 | 257.23 |
| GH1-19 | 1488 (495) | 0 | 0 | 1855 | 57.02 | 8355 | 140.04 |
| GH1-20 | 1539 (512) | 19 | 0.32 | 68 | 2.02 | 13 | 0.21 |
| GH1-21 | 1494 (497) | 0 | 0 | 162 | 4.96 | 2340 | 39.06 |
| GH1-22 | 1530 (509) | 0 | 0 | 309 | 9.24 | 521 | 8.49 |
| GH1-23 | 1024 (340) | 0 | 0 | 1 | 0.04 | 2 | 0.05 |
| GH1-24* | 1280 (426) | 12 | 0.24 | 185 | 6.61 | 339 | 6.61 |
| GH1-25* | 515 (171) | 0 | 0 | 0 | 0 | 1 | 0.05 |
| GH1-26* | 1345 (448) | 5 | 0.10 | 0 | 0 | 0 | 0 |
| GH1-27* | 491 (162) | 2 | 0.11 | 23 | 2.14 | 67 | 3.40 |
| GH1-28* | 625 (208) | 8 | 0.33 | 62 | 4.54 | 135 | 5.39 |
| | | | | | | | |
| GH31-1 | 3033 (1010) | 239 | 2.04 | 64 | 0.97 | 171 | 1.41 |
| GH31-2 | 2727 (908) | 9758 | 92.56 | 2607 | 43.72 | 11733 | 107.31 |
| GH31-3 | 2574 (857) | 2926 | 29.41 | 2823 | 50.16 | 9145 | 88.61 |
| GH31-4 | 2625 (874) | 829 | 8.17 | 6016 | 104.82 | 7971 | 75.73 |
| GH31-5 | 2559 (852) | 424 | 4.29 | 258 | 4.61 | 2352 | 22.92 |
| GH31-6 | 1881 (626) | 17 | 0.23 | 328 | 7.98 | 4535 | 60.13 |
| GH31-7 | 1917 (638) | 27 | 0.36 | 1280 | 30.54 | 30747 | 400.02 |
| GH31-8 | 1941 (646) | 19 | 0.25 | 73 | 1.72 | 397 | 5.10 |
| GH31-9 | 1908 (635) | 361 | 4.89 | 493 | 11.82 | 1175 | 15.36 |
| GH31-10* | 2781 (927) | 56 | 0.52 | 566 | 9.31 | 7182 | 64.41 |
| | | | | | | | |
| GH27-1 | 1236 (411) | 1870 | 39.11 | 1396 | 51.62 | 12256 | 247.10 |
| GH27-2 | 1338 (445) | 94 | 1.82 | 70 | 2.39 | 217 | 4.04 |

[a]Genes considered as not expressed (RPKM < 0.3 or the number of reads < 10) are marked with grey shade.
[b]Lengths of the transcript (bp) as well as its translated protein in parentheses (amino acids) are shown.
[c]The total numbers of paired-end reads before filtering are shown in parentheses. Note that although the reads were not filtered, we used 0 mismatch to map the reads to the assembled transcriptome. Thus reads that included any ambiguity including unknown nucleotide 'N' were not counted.
[d]The number of paired-end reads mapped.
*The partial ORFs, not including from start to stop codons.
